# Transcriptional landscape of pulmonary artery endothelium reveals subpopulation- and disease-specific remodeling signatures

**DOI:** 10.1038/s42003-026-10204-0

**Published:** 2026-05-11

**Authors:** Thomas Lins, Francesco Valzano, Elisabeth Fließer, Izabela Borek, Slaven Crnkovic, Mike Morley, Maria C. Basil, Jeremy Katzen, Edward Cantu, Jonas C. Schupp, Nikolaus Kneidinger, Jörg Lindenmann, Clemens Aigner, Alberto Benazzo, Edward E. Morrisey, Marek Bartkuhn, Leigh M. Marsh, Anna Birnhuber, Grazyna Kwapiszewska

**Affiliations:** 1 Otto Loewi Research Center, Lung Research Cluster, Medical University of Graz, Graz, Austria; 2 Division of Respiratory Medicine, Department of Internal Medicine, Lung Research Cluster, Medical University of Graz, Graz, Austria; 3 Institute for Lung Health, Cardiopulmonary Institute, German Lung Center, Justus-Liebig University, Giessen, Germany; 4 Penn-Chop Lung Biology Institute, University of Pennsylvania, Philadelphia, PA USA; 5 Department of Surgery, University of Pennsylvania, Philadelphia, PA USA; 6 Section of Pulmonary, Critical Care, and Sleep Medicine, Yale University School of Medicine, New Haven, CT USA; 7 Department of Pulmonary and Infectious Diseases, Hannover Medical School, Hannover, Germany; 8 Biomedical Research in End-Stage and Obstructive Lung Disease (BREATH), German Center for Lung Research BREATH, Hannover, Germany; 9 Department of Medicine V, University Hospital, LMU Munich, Comprehensive Pneumology Center (CPC), Member of the German Center for Lung Research (DZL), Munich, Germany; 10 Division of Thoracic and Hyperbaric Surgery, Medical University of Graz, Graz, Austria; 11 Division of Thoracic Surgery, Department of Surgery, Medical University of Vienna, Vienna, Austria; 12 Biomedical Informatics and Systems Medicine Science Unit for Basic and Clinical Medicine, German Lung Center, Giessen, Germany

**Keywords:** Bioinformatics, Disease genetics, Preclinical research, RNA

## Abstract

The physiological state of endothelial cells (ECs) is a central determinant of organ health. Distinct endothelial phenotypes and transcriptional programs are linked to vessel size and anatomical location, but it remains unclear how this intrinsic heterogeneity is organized within a particular niche. We performed compartment-specific single-cell profiling of human pulmonary artery (PA) ECs from healthy donors and from patients with two clinically divergent forms of pulmonary hypertension (PH): pulmonary arterial hypertension (PAH) and pulmonary hypertension associated with pulmonary fibrosis (PH-PF). We identified and localized three major EC subsets in healthy and remodeled PAs termed immuno-, vascular tone- and vascular plasticity modulatory, reflecting their enrichment for distinct biological processes. Expression signatures defining each subset were shared with other arterial beds, including the aorta and coronary arteries, and murine PA. Both PAH and PH-PF altered PAEC subset compositions, with increased immunomodulatory and decreased vascular plasticity modulatory PAECs. PH-PF PAECs were generally defined by dysregulated angiogenesis and antigen presentation, whereas PAH PAECs exhibited lipid metabolic dysfunction and enhanced vasoregulatory signaling. By uncovering endothelial heterogeneity and pathology-specific transcriptional programs in PAs, this study underscores the need of disease-specific therapeutic targeting.

## Abstract (web summary)

Integrative single-cell, spatial, and ligand–receptor analyses define PAEC heterogeneity across pulmonary hypertension entities, revealing functionally distinct endothelial states and disease-associated shifts in transcriptional programs.

## Introduction

Pulmonary arterial endothelial cells (PAECs) form a stable, low-turnover monolayer lining the interior of pulmonary arteries (PAs), where they regulate vascular tone, barrier integrity and maintain an anti-inflammatory and anti-proliferative surface^1–3^. Disruption of this homeostatic role—for example, due to injury or disease—can trigger endothelial dysfunction, a central driver in the pathogenesis of pulmonary hypertension (PH)^2,4–7^. PH is a progressive cardiopulmonary disorder defined by a mean pulmonary arterial pressure (mPAP) > 20 mm Hg and is associated with a 5-year mortality rate of 33–46%^5,8–11^. It is characterized by remodeling of PAs^12,13^, leading to luminal narrowing and vessel obliteration^14^. The remodeling cascade generally begins with endothelial injury, first leading to apoptosis and subsequently to a hyperproliferative, apoptosis-resistant, and pro-inflammatory endothelial state^15–17^. This shift is accompanied by reduced nitric oxide (NO) availability and elevated endothelin-1, further promoting vasoconstriction and pathological remodeling^14,18–20^. Similarly, endothelial activation and barrier disruption are also evident in pulmonary fibrosis (PF), particularly when complicated by PH^20–22^. Experimental lineage tracing in PH models demonstrated that ECs are enriched in antigen presentation pathways and adopt aberrant pro-angiogenic behavior^23,24^, showing their direct involvement in disease progression.

The advent of single-cell RNA sequencing (scRNA-seq) has revolutionized our understanding of EC heterogeneity and implicated endothelial dysfunction as a critical initiating factor in pulmonary vascular diseases^25,26^. Notably, in bleomycin-induced PF and Sugen5419/hypoxia-induced PH dynamic shifts in the EC composition were evidenced, pointing towards an emergence of pro-proliferative, pro-fibrotic, and metabolically deregulated EC subpopulations^19,27–30^. In line, RNA-seq studies using cultured PH patient-derived PAECs displayed cancer-like gene expression patterns, metabolic reprogramming, and a hyperproliferative, pro-inflammatory endothelial phenotype^31,32^. Recent efforts have further refined the spatial and functional classification of ECs by categorizing them either by vascular segment (arterial, venous, capillary, lymphatic)^33^ or by functionally distinct subsets, such as immune ECs, developmental ECs, and general capillary (cap1/gCap) versus alveolar capillary (cap2/aCap) ECs^34^. Despite the accumulating knowledge on transcriptional and functional changes of the endothelium in diseases involving the pulmonary vasculature^23,29,31–35^, and growing interest in endothelial heterogeneity, PAECs have not been examined at high resolution, particularly in clinically distinct subtypes such as PAH and PH associated with pulmonary fibrosis (PH-PF). While previous studies have described EC heterogeneity within venous (pulmonary venous and systemic venous) and capillary compartments (cap1/gCap vs. cap2/aCap), no such diversity has been reported within the PAEC population^36^. Given that arterial ECs constitute a relatively small fraction of lung cells, identifying distinct PAEC subpopulations is inherently challenging, and to date, there is no evidence whether specified PAEC subpopulations exist and contribute to the development of pulmonary vascular disease.

To address this, we here employed a compartment-specific approach to investigate PAEC heterogeneity in isolated medium-to-small PAs from donors and patients with either PAH or PH-PF. Our findings revealed a previously unrecognized heterogeneity within the PA endothelium, composed of immune-, vascular tone- and vascular plasticity-modulatory PAECs, which is conserved between different vascular beds, including the coronary arteries (CA) and aorta (AO), and in murine lungs. Moreover, distinct molecular PAEC signatures are associated with different forms of PH. This endothelial diversity may underlie the divergent vascular remodeling and treatment responses seen in PAH and PH-PF, underscoring the need for subtype- and disease-specific therapeutic strategies.

## Results

### Human PAECs partition into three distinct subpopulations enriched with specific transcriptional pathways

**Fig. 1:**
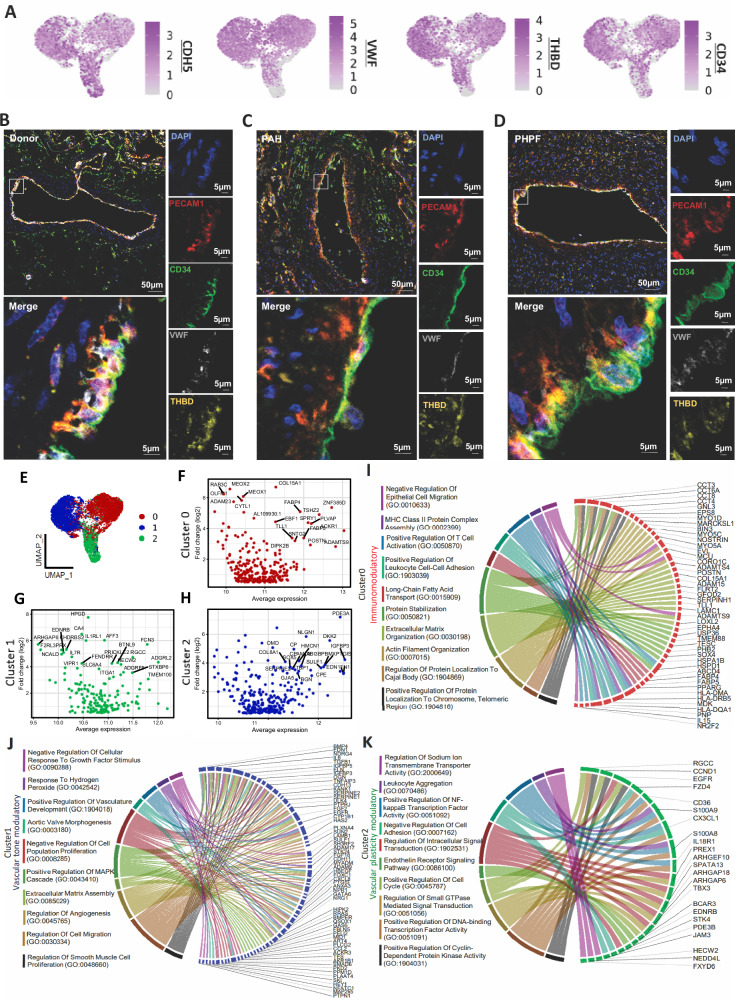
Human PAECs partition into three distinct subpopulations enriched with specific transcriptional pathways. **A** Uniform manifold approximation and projection (UMAP) expression plot of endothelial marker genes *CDH5*, *VWF*, *THBD*, and *CD34* within a subset of pulmonary arterial endothelial cells (PAECs). Color gradient represents normalized expression within each cell. **B** Immunofluorescence staining of human donor lung tissue sections (*n* = 5) depicting a pulmonary artery (PA) stained with the endothelial cell (EC) markers PECAM1 (red), CD34 (green), VWF (gray), and THBD (yellow). DAPI was used as a nuclear counterstain. The visualization includes an overview with a marked zoom area used for the subsequent images [white box], a merged image of all markers overlayed and images of each marker separately. The same visualization was chosen for **C** human pulmonary arterial hypertension (PAH) lung tissue samples (*n* = 3) and for **D** pulmonary hypertension with pulmonary fibrosis (PH-PF) lung tissue samples (*n* = 3). **E** UMAP of PAECs split into three distinct sub-clusters 0, 1, 2 determined by unbiased clustering. **F** MA plot for cluster0-specific upregulated differentially expressed genes (DEGs). **G** MA plot for cluster1-specific upregulated DEGs. **H** MA plot for cluster2-specific upregulated DEGs. Log2-foldchange (l2fc) values were calculated by MAST analysis, average expression is calculated from normalized mean data. Chord diagram showing the top 10 gene ontologies and their involved DEGs for the **I** immunomodulatory cluster, **J** the vascular tone modulatory cluster and **K** the vascular plasticity modulatory cluster. GOs are analyzed through enrichR and manually curated to exclude redundancy. Chord connection thickness is in relative correspondence to l2fc.

EC heterogeneity in PAs was evaluated using a single-cell transcriptomics approach. To achieve enrichment for the arterial compartment, we dissected and processed medium-to-small caliber arteries (2–5 mm) from lungs of healthy individuals (downsized donor tissue, *n* = 5) and end-stage explant lungs from patients with pulmonary vascular disease (*n* = 3 PAH and *n* = 3 PH-PF) (Table 1 and Supplementary Fig. 1A)^37,38^. No significant differences in demographic or clinical characteristics were observed between the PAH and PH-PF groups, with the sole exception of the mPAP. As expected, PAH patients had a significantly higher mPAP (PAH: 72 ± 4.6 mmHg vs. PH-PF: 34 ± 5.9 mmHg), reflecting the fact that vascular remodeling is the primary driver of disease in PAH, rather than a secondary consequence of some underlying pathology. Major cell population lineages were identified by *PTPRC*, *ACTA2*, *EPCAM, PDGFRA*, and *LYVE1* (10.5281/zenodo.17793613 Tables 1 and 2) for immune, smooth muscle, epithelial, fibroblast, and lymphatic ECs, respectively (Supplementary Fig. 1B, C). The EC population, identified by expression of *PECAM1* (Supplementary Fig. 1B, C), *CDH5*, *VWF, THBD*, and *CD34* (Fig. 1A–D and Supplementary Fig. 1D) was extracted and reclustered revealing 3 subpopulations (Fig. 1E), detectable in all cohorts (Supplementary Fig. 2A, B). Each cluster presented a unique set of enriched genes (Fig. 1F–H).

**Table 1:** Demographic data and clinical characteristics of the scRNAseq patient cohort

ID	Initial diagnosis	Age range [years]	Sex	Refined diagnosis	NYHA class	Smoking status	Pack years	mPAP [mmHG]	CI [L/min/m^2^]	PVR [dyn]	Comorbidities	Medication	Oxygen
Donor 1	Donor	71–80	f	*–*	*–*	Not active	<1	*na*	*na*	*na*	*na*	*na*	*–*
Donor 2	Donor	51–60	f	*–*	*–*	Not active	<5	*na*	*na*	*na*	*na*	*na*	*–*
Donor 3	Donor	51–60	f	*–*	*–*	Active	22.5	*na*	*na*	*na*	*na*	*na*	*–*
Donor 4	Donor	31–40	m	*–*	*–*	Not active		*na*	*na*	*na*	*na*	*na*	*–*
Donor 5	Donor	51–60	m	*–*	*–*	Not active	37	*na*	*na*	*na*	*na*	*na*	*–*
PAH 1	IPAH	31–40	m	IPAH	III	Never	0	71	*na*	*na*	Allergies: dust mite and animal dander	Treprostinil, Macitentan, Riociguat	Yes
PAH 2	IPAH	51–60	f	IPAH	III	Never	0	77	*na*	*na*	None	Treprostinil, Sildenafil, Bosentan	Yes
PAH 3	IPAH	61–70	m	PAH with CLD	III	Not active	50	68	*na*	*na*	COPD stage III with emphysema; Atherosclerotic heart disease; Hypercholesterolemia; Sigmoid diverticulosis; Colonic polyps/tubular adenoma (low-grade dysplasia)	Treprostinil, Sildenafil, Tiotropium/Olodaterol for inhalation	Yes
PH-PF 1	Fibrosis	61–70	m	HP	III	Not active	30	38	2.79	222	Type 2 diabetes mellitus; Kidney stones	Tiotropium and Ciclesonide for inhalation	Yes
PH-PF 2	Fibrosis	61–70	m	IPF	III	Not active	35	36	*na*	670	Stroke with global aphasia, right hemiplegia, and incomplete visual field defect; Pulmonary embolism with systemic thrombolysis and intracranial embolectomy; Patent foramen ovale (PFO)	Nintedanib, Prednisolone, Pantoprazole	Yes
PH-PF 3	Fibrosis	51–60	m	HP	III	Not active	5	27	2.66	320	Occupational exposure: possible isocyanate	Azathioprine, Prednisolone, Trimethoprim/Sulfamethoxazole (prophylaxis), Budesonide, Pantoprazole	Yes
*p* value	*–*	0.201	*–*	*–*	*–*	*–*	0.744	**0.001**	*–*	*–*	*–*	*–*	*–*

*p* value compares PAH and PH-PF groups. Part of this data has already been published previously^37^.

*BMI* body mass index, *NYHA Class* New York Heart Association class, *mPAP* mean pulmonary arterial pressure, *CI* cardiac index, *PVR* pulmonary vascular resistance, *IPAH* idiopathic pulmonary hypertension, *CLD* chronic lung disease, COPD chronic obstructive pulmonary disease, *HP* hypersensitivity pneumonitis, *IPF* idiopathic pulmonary fibrosis.

**Table 2:** Utilized antibodies for tissue staining

Antibody	Producer	Host	Dilution	ID
CD31	Abcam	Rabbit	1:100	Abcam Cat# ab32457, RRID:AB_726369
CD34	BioRad	Mouse	1:500	Bio-Rad Cat# MCA547G, RRID:AB_1125255
THBD	Invitrogen	Rabbit	1:100	(Thermo Fisher Scientific Cat# PA5-21924, RRID:AB_11152826)
VE-CAD	R&D	Goat	1:100	R and D Systems Cat# AF938, RRID:AB_355726
VWF	Agilent	Rabbit	1:300	Agilent Code Num.# GA527
COL15A1	Invitrogen	Rabbit	1:100	(Thermo Fisher Scientific Cat# PA5-53667, RRID:AB_2640011)
ACKR1	Atlas Antibodies	Rabbit	1:100	Atlas Antibodies Cat# HPA017258, RRID:AB_1845997
PRX	Sigma-Aldrich	Rabbit	1:100	Sigma-Aldrich Cat# HPA001868, RRID:AB_2172440
HPGD	Sigma-Aldrich	Rabbit	1:100	Sigma-Aldrich Cat# HPA004919, RRID:AB_1079081
PTGIS	Atlas Antibodies	Rabbit	1:100	Sigma-Aldrich Cat# HPA014193, RRID:AB_1855746
SULF1	abcam	Rabbit	1:100	Abcam Cat# ab32763, RRID:AB_882749

Genes enriched in cluster 0 (Fig. 1F and Supplementary Fig. 3A) represented gene ontologies (GOs) linked to immune cell regulation (MHC class II protein complex assembly (GO:0002399), positive regulation of T-cell activation (GO:0050870) and positive regulation of leukocyte cell-cell adhesion (GO:1903039), e.g., *IL15, PNP, MDK, HLA-DQA1*, *HLA-DRB5, HSPD1*. Further, pathways such as protein stabilization (GO:0050821), localization (GO:1904869) and positive regulation of protein localization to chromosome, telomeric region (GO:1904816), e.g., *CCT3*, *CCT6A*, *CCT8*, *CCT4*, and *GNL3* as well as extracellular matrix (ECM) organization (GO:0030198) e.g., *ADAMTS4, ADAM15*, *FLRT2, GFOD2*, and *POSTN* were enriched (Fig. 1I, 10.5281/zenodo.17793613 Tables 3 and 4).

Cluster 1 enriched genes (Fig. 1G and Supplementary Fig. 3B) were involved in vascular development and maintenance (positive regulation of vasculature development (GO:1904018) and regulation of angiogenesis (GO:0045765)) e.g., *NRG1, GATA6, NPR1, ANXA3, PTGIS, CXCL8* and *FOXC1*, regulation of smooth muscle cell (SMC) proliferation (GO:0048660) e.g., *BMP4, EDN1, NDRG4, IL6* and ECM assembly (GO:0085029) e.g., *GAS6, BMPER, FGF2, FGF18* and *HAS2*, as well as in biological processes such as regulation of cell migration (GO:0030334) e.g., *HBEGF, ADAM9, MYADM, CDH11, FGF18, STK24, EGFR, SULF1* and *PLXNA4* or negative regulation of cell population proliferation (GO:0008285), e.g., *HEY1, SKI, PLAAT4, PKD2, SMAD6, AKR1B1* and *ACKR3* (Fig. 1J, 10.5281/zenodo.17793613 Tables 5 and 6).

Cluster 2 featured enriched genes (Fig. 1H and Supplementary Fig. 3C) primarily connected to biological processes including cell cycle regulation (positive regulation of cell cycle (GO:0045787) and positive regulation of cyclin-dependent protein kinase activity (GO:1904031) e.g., *TBX3*, *RGCC*, *CCND1*, *EGFR*, *JAM3*, *PDE3B*, *CX3CL1* and *FZD4*, regulation of intracellular signal transduction (GO:1902531), regulation of small GTPase–mediated signal transduction (GO:0051056), including regulators of Rho/Rac family GTPase activity e.g., *ARHGAP6*, *ARHGAP18*, *SPATA13*, *ARHGEF10* and *PREX1*, as well as endothelin receptor signaling pathway (GO:0086100), positive regulation of DNA-binding transcription factor activity (GO:0051091) and NF-κB signaling (GO:0051092) e.g., *CD36*, *S100A8*, *S100A9*, *CX3CL1*, *IL18R1* (Fig. 1K, 10.5281/zenodo.17793613 Tables 7 and 8).

Based on these enriched genes and the associated GOs, we termed cluster 0 “immunomodulatory”, cluster 1 “vascular tone modulatory” and cluster 2 “vascular plasticity modulatory” PAECs (Fig. 1I–K).

### The pulmonary artery endothelium is constituted by a mosaic of EC subpopulations

**Fig. 2:**
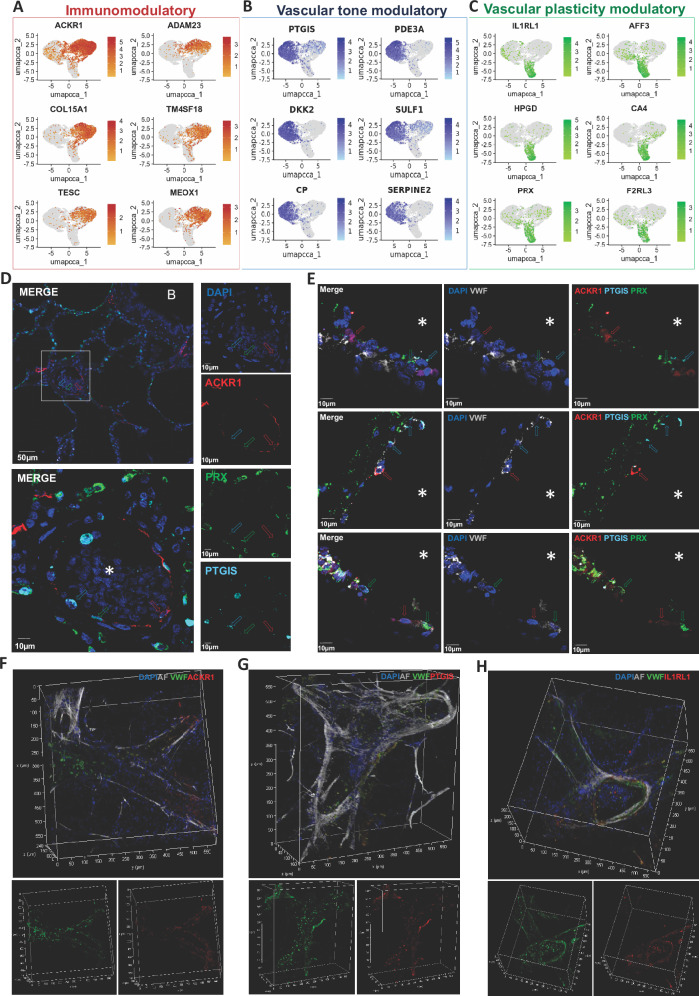
PAEC subpopulations are marked by gene expression profiles enriched in non-arterial pulmonary vascular compartments. **A** Uniform manifold Projection (UMAP) expression plots of the pulmonary arterial endothelial cell (PAEC) subset (5 Donor, 3 PAH, 3 PH-PF) depicting marker genes with high specificity to the immunomodulatory cluster (*ACKR1, ADAM23, COL15A1, TM4SF18, TESC, MEOX1*), **B** the vascular tone modulatory cluster (*PTGIS, PDE3A, DKK2, SULF1, CP, SERPINE2*) and **C** the vascular plasticity modulatory cluster (*IL1RL1, AFF3, HPGD, CA4, PRX, F2RL3*), **D** Formalin-fixed paraffin-embedded FFPE donor lung tissue sections (*n* = 3) stained for ACKR1 (red), PRX (green), PTGIS (cyan) and nuclear counterstain DAPI (blue). The merged overview image (top left) shows the analyzed region, with the white box indicating the area displayed at higher magnification below. The merged zoom is shown together with the corresponding single-channel images. The asterisk marks the vessel lumen and “B” denotes a bronchus. Arrows indicate cells expressing the respective marker (color-coded). **E** Representative images of FFPE pulmonary arteries (2–5 mm diameter). The left panel shows the full merged image, the middle panel shows VWF and DAPI staining, and the right panel displays endothelial subpopulation markers (ACKR1 (red), PTGIS (cyan), PRX (green)). Asterisks denote the vessel lumen. Arrows highlight marker-positive endothelial cells (ECs) with minimal overlap between marker signals. Representative 3-dimensional (3D) immunofluorescence images of cleared precision cut lung slices (PCLS, thickness = 300 µm) from control patients stained against vWF (green) and **F** ACKR1 (red, **F**), PTGIS (red, **G**) and IL1RL1 (red, **H**). Auto florescence (AF, gray) was used to visualize the vessel via elastin and collagen.

Immunomodulatory PAECs-defining markers were *ACKR1, ADAM23*, *COL15A1*, *TM4SF18*, *TESC*, and *MEOX1* (Fig. 2A). Vascular tone modulatory PAECs were enriched in *PTGIS*, *PDE3A*, *DKK2*, *SULF1*, *CP*, and *SERPINE2* (Fig. 2B) and vascular plasticity modulatory PAECs were defined by expression of *IL1RL1*, *AFF3*, *HPGD*, *CA4*, *PRX*, and *F2RL3* (Fig. 2C). As several PAEC subpopulation-enriched genes, such as *ACKR1* and *PRX*, have previously been reported as compartment-defining markers distinguishing venous/vasa vasorum and capillary ECs in the lung^25,39^, we sought to exclude a major contribution from capillary ECs derived from parenchymal contaminations. First, we used the epithelial cell population as a quantitative indicator of parenchymal contamination. Epithelial cell fractions were low across samples and did not correlate with endothelial subcluster distribution (Supplementary Fig. 4A–C). Second, we filtered *VWF*-positive cells, as *VWF* distinguishes macrovascular from capillary ECs in the lung, where its expression is usually low or absent^40,41^. Restricting the analysis to *VWF*-positive cells preserved the same pattern of subpopulation-enriched gene expression (Supplementary Fig. 5A–D). Together, these results indicate that arterial endothelial heterogeneity was not driven by capillary contaminations.

To validate the presence of these markers in PAECs on the protein level, we performed immunofluorescence imaging on donor lung transplant samples. Stainings confirmed COL15A1⁺ and ACKR1⁺ cells, corresponding to the immunomodulatory PAEC population, within the endothelial layer of VWF^+^ PAs (Supplementary Fig. 6A). Of note, collagen type XV alpha 1 (COL15A1) was predominantly expressed in peribronchial vessels and regions of ECM deposition (Supplementary Fig. 7A–C), whereas atypical chemokine receptor 1 (ACKR1) was primarily detected in pulmonary veins (Supplementary Fig. 7D–G), consistent with their known localization^36^. Immunostaining for periaxin (PRX) and 15-hydroxyprostaglandin dehydrogenase (HPGD), previously described as capillary endothelial markers, showed a strong signal in capillary networks as expected; however, individual PRX⁺ and HPGD⁺ cells, representing the vascular plasticity modulatory cluster, were also detectable within the endothelial layer of PAs (Supplementary Figs. 8A–C and 6B). In parallel, PTGIS⁺ and SULF1⁺ ECs corresponding to the vascular tone–modulatory cluster were observed within the pulmonary arterial vasculature (Supplementary Fig. 6C).

Importantly, multiplex staining on donor lung tissue (Fig. 2D) and isolated PAs (Fig. 2E) showed the simultaneous presence of all PAEC subpopulation markers within the PA endothelium, further confirming the heterogeneity of endothelial subpopulations as suggested by our scRNA-seq data. Additionally, three-dimensional (3D) tissue reconstruction verified spatial expression of ACKR1^+^, PTGIS^+^, and interleukin-1 receptor-like 1 (IL1RL1^+^) subpopulations within the PA tree (Fig. 2F–H). Together, these images corroborate the presence of three EC subpopulations within the endothelium of PAs.

**Fig. 3:**
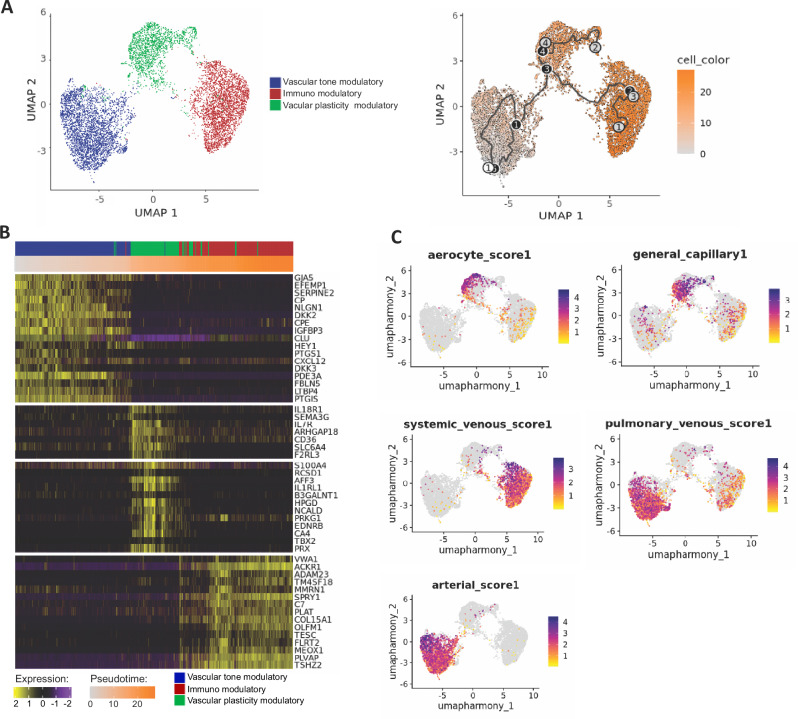
The pulmonary artery endothelium gradually shifts between subpopulations. **A** Uniform manifold projection (UMAP) embedding of pulmonary arterial endothelial cells (PAECs) with trajectory inference performed using Monocle3 colored by the three PAEC subpopulations (left) and as inferred principal graph overlaid on the same UMAP, showing the proposed “developmental” trajectory (right). Black numbered nodes indicate branch points (graph nodes), and gray numbered circles indicate terminal states (leaves). Cells are colored by pseudotime, which was calculated as the geodesic distance along the principal graph from a manually specified root node located at the bottom left. Pseudotime increases along the trajectory away from this root. **B** Heatmap of marker genes from EC subpopulations as described in literature^36^, as well as markers from identified PAEC subpopulations, correlating expression and pseudotime. **C** UMAPs depicting similarity scores of common EC subtype markers calculated with Seurat.

To better understand the potential origin and trajectory of the identified PAEC subpopulations, we performed pseudo-time analyses outlining potential cell state transitions (Fig. 3A). Analysis of marker gene expression with pseudotime progression revealed gradual transcriptional shifts among the PAEC subpopulations, with intermediate cell states showing expression of markers from two clusters with decreased expression strength (Fig. 3B).

Taken together, we here reveal a mosaic, gradient-like composition of ECs within PAs composed of three major subsets. This observation raises the question of whether distinct expression profiles better reflect functional than anatomical features. Indeed, transcriptional similarities of PAEC subpopulations with ECs from different pulmonary vascular beds could be observed (Fig. 3C, 10.5281/zenodo.17793613 Table 9).

### Shared arterial EC heterogeneity between different human vascular beds and murine lungs

**Fig. 4:**
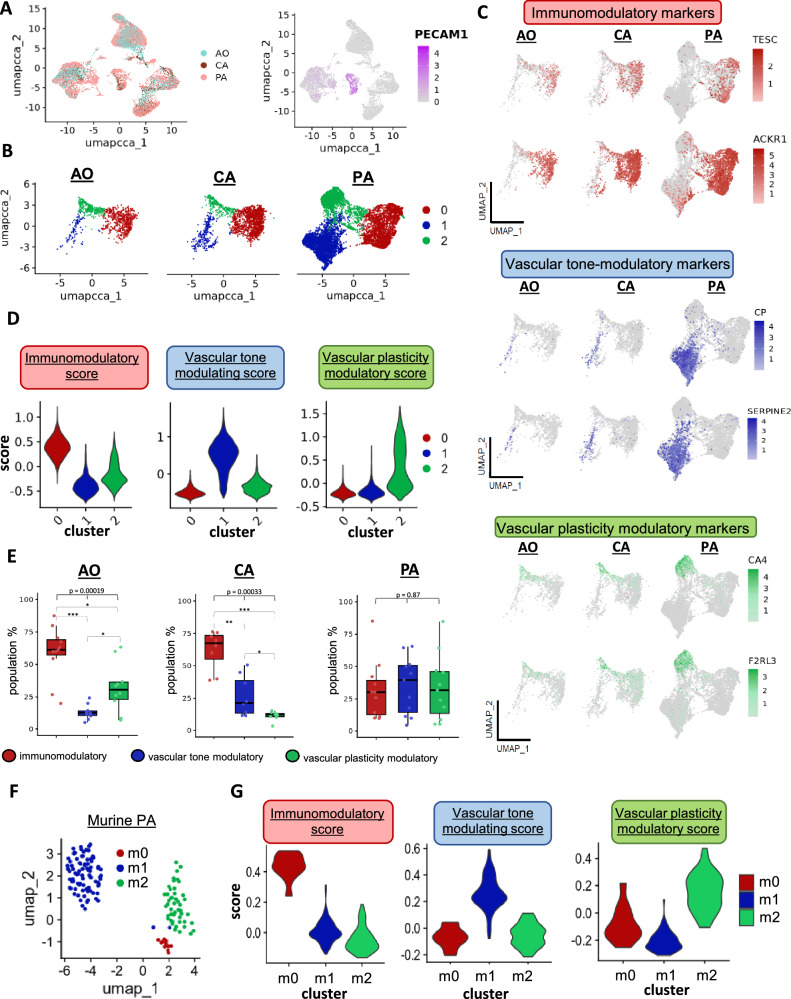
Endothelial heterogeneity and distribution across different human vascular beds and across different organisms. **A** Uniform manifold approximation and projection (UMAP) plot of a concatenated dataset of aorta (AO, *n* = 11), coronary artery (CA, *n* = 8) and pulmonary artery (PA, *n* = 12), differentiating the three vascular beds (left panel) and highlighting endothelial cells (ECs) via PECAM1 (right panel). **B** UMAP visualization of endothelial subcluster 0, 1 and 2 in AO, CA and PA. **C** UMAP expression plots of immunomodulatory EC markers TESC and ACKR1 (top), vascular tone modulatory EC markers CP and SERPINE2 (middle) and vascular plasticity modulatory EC markers CA4 and F2RL3 (bottom) split with regards to vessel compartments AO (left), CA (middle) and PA (right). **D** Violin plots of EC subpopulation correlation scores calculated from the top 30 differentiating marker genes per cluster, within the combined AO, CA and PA endothelial compartment. **E** Boxplots show the per-sample percentage distribution (median, interquartile range, and 1.5× IQR whiskers) of PAEC subpopulations in AO, CA and PA, respectively, with individual samples overlaid. Statistical significance was assessed using Kruskal–Wallis tests followed by pairwise Wilcoxon rank-sum tests with Holm correction. **F** UMAP of murine wild-type PAEC split into three distinct clusters (m0, m1, m2) based on unbiased clustering. **G** Violin plots of murine PAEC correlation scores calculated for the immunomodulatory, vascular tone modulatory and vascular plasticity modulatory clusters. Correlation scores were calculated from the murine genes equivalent to the top 30 cluster-defining genes of the human immunomodulatory, vascular tone modulatory and vascular plasticity modulatory EC populations.

To investigate whether the same specialized EC subtypes can be found in the systemic circulation, such as aorta (AO) or coronary arteries (CA), we analyzed publicly available datasets GSE155468 (AO; *n* = 8)^42^ and GSE131778 (CA; *n* = 11)^43^. Integration of these datasets resulted in a homogenous distribution of cells of all three datasets (Fig. 4A). Major clusters in the combined dataset (Fig. 4B) were maintained in CA and AO, as indicated by expression of *TESC* and *ACKR1* (immunomodulatory), *CP* and *SERPINE2* (vascular tone modulatory) and *CA4* and *F2RL3* (vascular plasticity modulatory) (Fig. 4C). Furthermore, cluster-specific correlation scores calculated based on the top 30 differentially expressed genes (DEGs) of each PA sub cluster, confirmed that EC sub clusters from a concatenated dataset of AO, CA and PA datasets correspond to the identified human PAEC subpopulations (Fig. 4D, 10.5281/zenodo.17793613 Table 10). Interestingly, while EC subpopulations identified in the human PAs are conserved throughout different vascular beds, their proportions vary, with a pronounced increase of the immunomodulatory EC cluster in AO and CA as compared to PAs (Fig. 4E, 10.5281/zenodo.17793613 Tables 11–14).

Next, we assessed whether similar EC subpopulations exist in murine PAs. Clustering of extracted PAECs from our murine PA scRNA-seq dataset (GSE 210248)^37^ again revealed three distinct populations (cluster m0-m2; Fig. 4F). Based on correlation scores with murine gene homologs (10.5281/zenodo.17793613 Table 15), these indeed correspond to the identified immunomodulatory, vascular tone modulatory and vascular plasticity modulatory subpopulations (Fig. 4G, 10.5281/zenodo.17793613 Table 16). These results indicate that endothelial transcriptional heterogeneity is conserved not only across different vascular beds but also across species.

### Pulmonary vascular disease alters the PAEC transcriptome

**Fig. 5:**
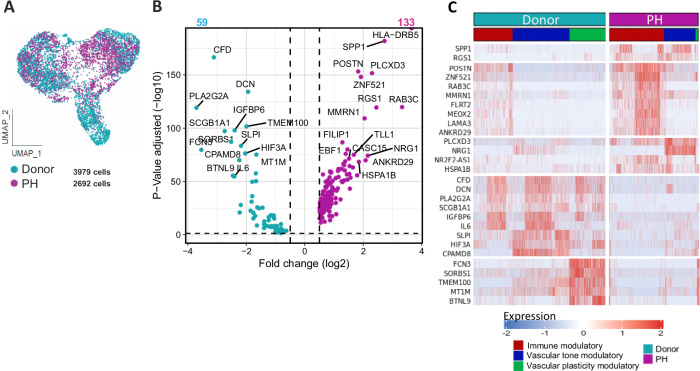
PAEC subpopulations undergo distinct changes during PH disease progression. **A** Uniform manifold approximation and projection (UMAP) plot of donor and pulmonary hypertension (PH) pulmonary arterial endothelial cells (PAEC). **B** Volcano plot showing differentially expressed genes (DEGs) between donor and PH PAECs. The applied filtering approves genes with adjusted *p* value of <0.05 and log2-fold change (l2fc) > 0.5 (magenta) or <−0.5 (cyan). **C** Heatmap of the top 15 up and top 15 down regulated genes in donor vs. PH PAECs ordered according to the identified endothelial subpopulations.

To understand the effects of PH on distinct PAEC sub clusters, we analyzed transcriptional changes in PAs of donor versus PH lungs (Fig. 5A). Diseased PAECs displayed significant transcriptional reprograming (Fig. 5B, 10.5281/zenodo.17793613 Table 17) with both global and subset-specific changes (Fig. 5C). For example, *SPP1* or *RGS1* were increased in all three clusters, *HIF3A1* was upregulated in the immunomodulatory and in the vascular plasticity modulatory cluster and *DCN* was downregulated in all three subpopulations (Fig. 5C), prompting a deeper investigation of PH-induced transcriptomic signatures within each cluster.

### PH induces distinct transcriptional reprogramming in each PAEC subpopulation

**Fig. 6:**
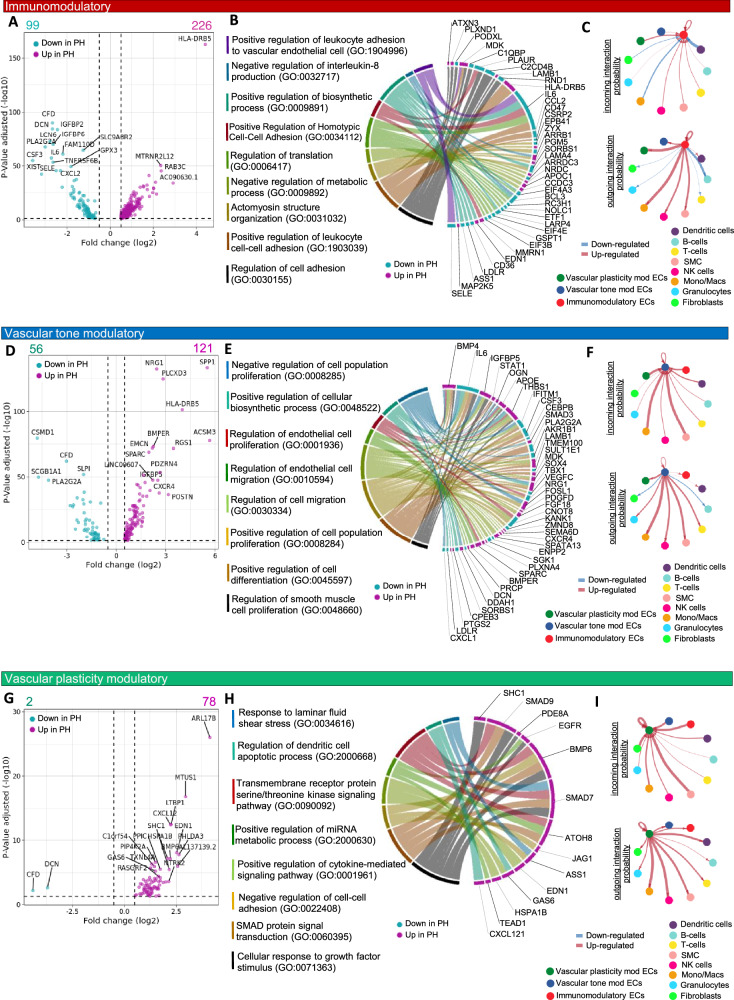
Vascular remodeling induces distinct transcriptional reprogramming in each PAEC subpopulation. **A** Volcano plot showing differentially expressed genes between the donor and pulmonary hypertension (PH) immunomodulatory endothelial cluster. The applied filtering approves genes with adjusted *p* value of <0.05 and log2-fold change (l2fc) > 0.5 (magenta) or <−0.5 (cyan). **B** Chord diagram showing the most significantly regulated gene ontologies (GOs) within the immunomodulatory population in PH. Chord connection thickness is in relative correspondence to l2fc. **C** Circle plots showing the incoming and outgoing interaction probabilities between the immunomodulatory endothelial subpopulation and other cell types based on ligand receptor interactions. Line thickness corresponds to how strongly the interaction probability is affected, while color indicates up- or down regulation. **D** Volcano plot showing DEGs between the donor and PH vascular tone modulatory endothelial cluster (calculated as described in (**A**)). **E** Chord diagram showing the most significantly regulated GOs within the vascular tone modulatory cluster in PH. Chord connection thickness is in relative correspondence to l2fc. **F** Circle plots showing the incoming and outgoing interaction probabilities between the vascular tone modulatory endothelial subpopulation and other cell types based on ligand receptor interactions. **G** Volcano plot showing DEGs between the donor and PH vascular plasticity modulatory endothelial cluster. **H** Chord diagram showing the most significantly regulated GOs within the vascular plasticity modulatory cluster in PH. **I** Circle plots showing the incoming and outgoing interaction probabilities between vascular plasticity modulatory endothelial subpopulation and other cell types based on ligand receptor interactions. Line thickness corresponds to how strongly the interaction probability is affected, while color indicates up- or down-regulation.

Genes regulated in the immunomodulatory subpopulation in PH (Fig. 6A, 10.5281/zenodo.17793613 Table 18) were enriched in GO pathways related to immune cell/leukocyte adhesion (GO:1904996 positive regulation of leukocyte adhesion to vascular EC) as well as metabolic and biosynthetic processes (GO:0009892 positive regulation of biosynthetic process, GO:0009892 negative regulation of metabolic process; Fig. 6B 10.5281/zenodo.17793613 Table 19), indicated by dysregulated expression of e.g., *HLA-DRB5*, *MMRN1, PDE4B, PODXL, LDLR*, and *CD36* (Supplementary Fig. 9A). Cell-cell communication analysis suggested that immunomodulatory PAECs received increased signaling from NK cells, T cells, B cells, SMCs, and vascular plasticity modulatory PAECs, while their outgoing signals primarily targeted monocytes/macrophages and NK cells (Fig. 6C, 10.5281/zenodo.17793613 Table 20).

PH-regulated genes in the vascular tone modulatory PAEC population (Fig. 6D, 10.5281/zenodo.17793613 Table 21) related to the regulation of PAEC and SMC proliferation, migration- and differentiation (e.g., regulation of endothelial cell proliferation (GO:0001936) and migration (GO:0010594) or regulation of SMC proliferation (GO:0048660); Fig. 6E, 10.5281/zenodo.17793613 Table 22). Upregulated genes included *BMP4*, *SOX4* and *SPARC*, all involved in vascular development, while *TMEM100*, *CSF3* and *PTGS2*—involved in EC injury, regeneration and repair—were downregulated (Supplementary Fig. 9B). In accordance with the identified GO pathways, PAECs involved in vascular tone modulation exhibited markedly increased interaction probabilities with SMCs and enhanced cross-talk with immune cells including granulocytes, monocytes/macrophages, and T-cells (Fig. 6F, 10.5281/zenodo.17793613 Table 20).

Genes enriched in the vascular plasticity modulatory cluster in PH (e.g., *SMAD9, EGFR, BMP6, DCN* and *CFD*; Fig. 6G and Supplementary Fig. 9C, 10.5281/zenodo.17793613 Table 23) were related to cellular response and signal transduction pathways (e.g., cellular response to growth factor stimulus and signal transduction (GO:0071363), SMAD protein signal transduction (GO:0060395), transmembrane receptor protein serine/threonine kinase signaling pathway (GO:0090092), and response to laminar fluid shear stress (GO:0034616)) (Fig. 6H, 10.5281/zenodo.17793613 Table 24). All incoming (in particular from SMCs and immunomodulatory ECs) and outgoing (in particular to monocytes) interaction probabilities of vascular plasticity modulatory PAECs were increased (Fig. 6I, 10.5281/zenodo.17793613 Table 20).

In mice, hypoxia induced a shift in PAEC heterogeneity, resulting in two different endothelial clusters corresponding to vascular plasticity—and vascular tone modulatory PAECs, while immunomodulatory PAECs were absent. Notably, the vascular tone modulatory cluster decreased and the vascular plasticity modulatory cluster increased, although analysis was limited by the low number of PAECs (Supplementary Fig. 10A–C).

### PAH and PH-PF have distinct effects on PAEC subpopulation distribution

**Fig. 7:**
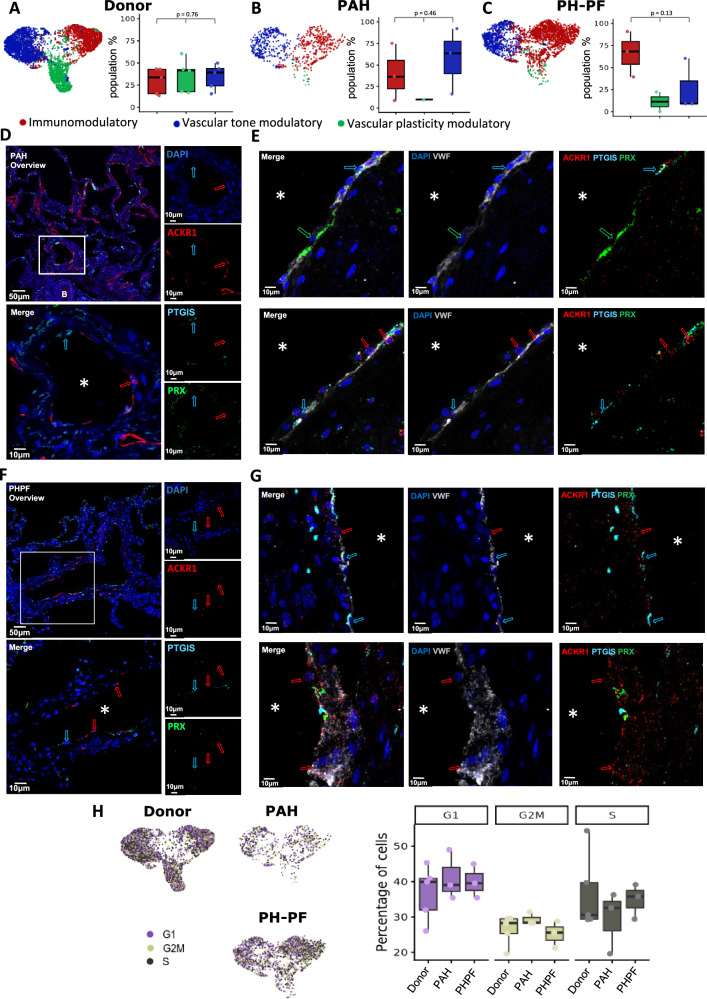
PAH and PH-PF show differing PAEC sub-populational distribution. **A**–**C** UMAP projections (left) and corresponding boxplots (right) showing pulmonary arterial endothelial cell (PAEC) subpopulation distributions in **A** donor, **B** pulmonary arterial hypertension (PAH), and **C** pulmonary hypertension with pulmonary fibrosis (PH-PF) samples. Subpopulation proportions are presented as medians with interquartile range (IQR) derived from sample-level percentages. Statistical significance was assessed using Kruskal–Wallis tests followed by pairwise Wilcoxon rank-sum tests with Holm correction. **D** Formalin-fixed paraffin-embedded (FFPE) PAH lung tissue sections (*n* = 2) stained for ACKR1 (red), PTGIS (cyan), PRX (green), VWF (white) and nuclear counterstain DAPI (blue). Overview images (top left) indicate the analyzed region (white box), with higher magnification views shown below, including merged and single-channel images. The asterisk (*) marks the vessel lumen and “B” denotes a bronchus. Arrows indicate cells expressing the specified marker. **E** FFPE PH-PF lung tissue sections (*n* = 2) stained for ACKR1 (red), PTGIS (cyan), PRX (green), VWF (white) and nuclear counterstain DAPI (blue). Overview images (top left) indicate the analyzed region (white box), with higher magnification views shown below, including merged and single-channel images. The asterisk (*) marks the vessel lumen and “B” denotes a bronchus. Arrows indicate cells expressing the specified marker. **F** Representative pulmonary artery (2–5 mm diameter) images from three distinct regions of PAH patient samples. Panels display merged images (left), DAPI/VWF staining (middle) and endothelial subpopulation markers (ACKR1, PTGIS, PRX; right). Asterisks indicate the lumen; arrows highlight marker-positive endothelial cells with limited signal overlap. **G** Representative pulmonary artery (2–5 mm diameter) images from three distinct regions of PH-PF patient samples. Panels display merged images (left), DAPI/VWF staining (middle) and endothelial subpopulation markers (ACKR1, PTGIS, PRX; right). Asterisks indicate the lumen; arrows highlight marker-positive endothelial cells with limited signal overlap, **H** UMAPs (left) and boxplots (right) illustrating endothelial cell cycle state distributions across donor, PAH, and PH-PF samples. Statistical significance was assessed using Kruskal–Wallis tests followed by pairwise Wilcoxon rank-sum tests with Holm correction. No significance was observed.

Next, we aimed to identify PH-PF and PAH specific changes in the three PAEC clusters. All subpopulations were present in PAH and PH-PF (Fig. 7A–C). In both PH-PF and PAH, a marked reduction of vascular plasticity modulatory PAECs with concomitant increase of immunomodulatory PAECs was observed compared to donors (Fig. 7A–C, 10.5281/zenodo.17793613 Table 25). Additionally, the proportion of vascular tone modulatory PAECs in PAH (Fig. 7B) and PH-PF shows a decreasing trend (Fig. 7C).

To determine whether endothelial subpopulation marker expression is preserved on the protein level in PH - associated vascular remodeling, we performed in situ validation using multiplex immunofluorescence stainings of FFPE lung tissue sections and isolated PAs from patients with PAH and PH-PF (Fig. 7D–G). By doing so, we indeed identified ECs expressing markers of the immunomodulatory (ACKR1, red arrows), vascular tone modulatory (PTGIS, blue arrows), and vascular plasticity modulatory (PRX, green arrows) clusters within the vascular endothelium of PAH and PH-PF patients. While ACKR1⁺ and PTGIS⁺ ECs were readily detectable in pulmonary vessels, PRX-expressing cells were less frequent (Fig. 7D, E). Analysis of isolated PAs from the same disease groups confirmed the presence of the ACKR1, PTGIS and PRX-positive ECs along the intimal layer of remodeled arteries (Fig. 7F, G). Consistently, dual immunofluorescence staining on patient-derived lung tissue sections further supported the presence of immunomodulatory (COL15A1/ACKR1), vascular tone modulatory (SULF1/PTGIS), and vascular plasticity modulatory (PRX/HPGD) PAEC populations in both PAH and PH-PF samples (Supplementary Fig. 11A–F). These stainings highlight selective marker expression and support the notion of endothelial mosaicism in PAs.

Additionally, we examined whether the shifts in PAEC subset proportions in PAH and PH-PF lungs reflected an altered cell cycle progression. However, no apparent changes in cell cycle, apoptosis or senescence gene expression signatures compared to donors were detected (Fig. 7H, Supplementary Fig. 12A, B; 10.5281/zenodo.17793613 Table 26). In addition, our analysis does not point towards EndoMT in any of the three subpopulations (Supplementary Fig. 13A–C). Thus, the proportional shifts of EC subpopulations within medium-to-small caliber PAs (2–5 mm) likely result from dynamic transcriptional reprogramming rather than differences in proliferation or cell survival.

### EC subpopulations show diverging regulatory effects between PAH and PH-PF

**Fig. 8:**
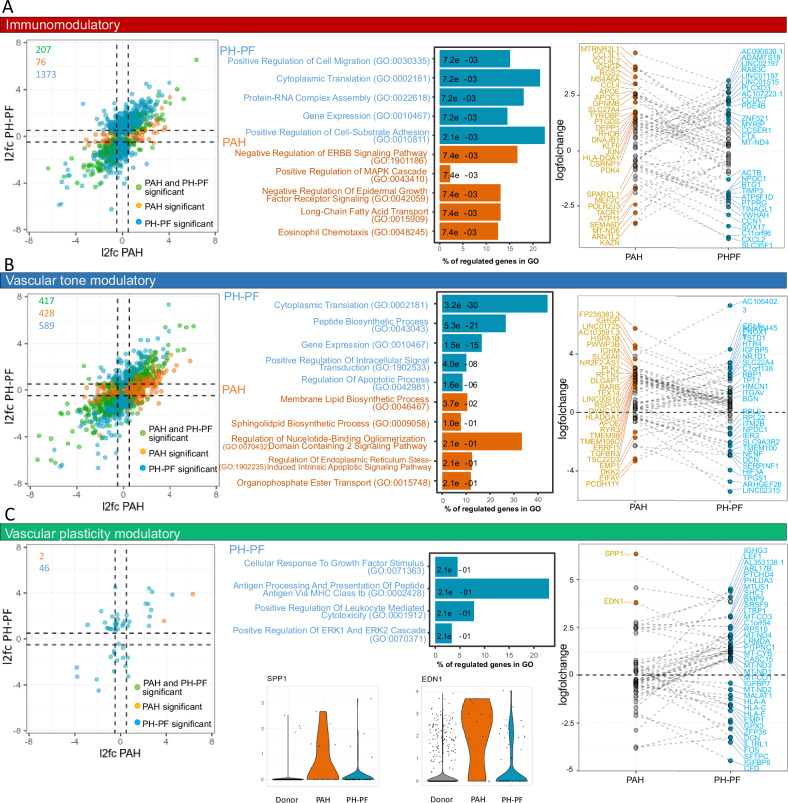
EC subtypes show diverging regulatory effects between PAH and PH-PF. **A** Scatterplot (left) of differentially expressed genes (DEGs) in immunomodulatory pulmonary arterial endothelial cells (PAEC) plotting log2-fold change (l2fc) of pulmonary arterial hypertension (PAH) on the *x*-axis and l2fc of pulmonary hypertension with pulmonary fibrosis (PH-PF) on the *y*-axis. Bar graph (middle) of top 5 gene ontologies (GOs) from PAH and PH-PF of disease-specific significant DEGs from immunomodulatory PAECs. *p* values are included in the bars. Pairwise comparison (right) of the top 30 DEGs from PH-PF and PAH in immunomodulatory DEGs. **B** Scatterplot (left) of DEGs in vascular tone modulatory PAECs plotting l2fc PAH on the *x*-axis and l2fc PH-PF on the *y*-axis. Bar graph (middle) of top 5 GOs of disease-specific significant DEGs from vascular tone modulatory PAECs. *p* values are included in the bars. Pairwise comparison (right) of the top 30 DEGs from PH-PF and PAH in vascular tone modulatory PAECs. **C** Scatterplot (left) of DEGs in vascular plasticity modulatory PAECs plotting l2fc PAH on the *x*-axis and l2fc PH-PF on the *y*-axis. Bar graph (middle) of top 5 GOs of disease-specific significant DEGs from vascular plasticity modulatory PAECs in PH-PF. *p* values are included in the bars. Violin plots below show the only two genes specifically upregulated in PAH in vascular plasticity modulatory PAECs (SPP1 and EDN1). Pairwise comparison (right) of the top 30 DEGs from PH-PF and PAH in vascular plasticity modulatory PAECs.

Subsequently, we explored whether the presence of PAH or PH-PF elicits distinct transcriptomic fingerprints within the different endothelial subpopulations. Genes regulated specifically in the immunomodulatory cluster of PH-PF were associated with pathways related to gene expression, cytoplasmic translation, e.g., *RPLP0, RPS3A, RPL23A* and protein-RNA complex assembly, e.g., *RPLP0, RPF2, RPS15, SNRPD1*. PAH-specific genes in this cluster contributed to pathways such as long-chain fatty acid transport, e.g., *FABP5, APOE, SLC27A4* and regulation of diverse signaling pathways, such as ERBB signaling, EGFR signaling and MAPK cascade signaling, e.g., *CCL3L1, GPNMB, CCL4, APOE* (Fig. 8A, 10.5281/zenodo.17793613 Tables 27–29). PH-PF specific DEGs in the vascular tone modulatory cluster also related to gene expression and cytoplasmic translation as well as to positive regulation of intracellular signal transduction, e.g., *SEMA5A, HHIP, AKAP13, C1QBP, GPER1, RACK1* and regulation of apoptotic process, e.g., *HHIP, CLU, SH3RF1, MYC, C1QBP, GPER1, CFL1*. In PAH, DEGs were linked to membrane lipid biosynthesis and sphingolipid biosynthetic process e.g., *HACD1, CERS6, DEGS1, PLPP3*  and regulation of nucleotide binding oligomerization (*HSPA1B*) (Fig. 8B, 10.5281/zenodo.17793613 Tables 30–32). In the vascular plasticity modulatory cluster, only two genes were upregulated in PAH, *SPP1* and *EDN1*. PH-PF-specific genes of this cluster were associated with signaling pathways linked to growth factor response and cell proliferation/differentiation (*FOS*), as well as immune cell activation (e.g., *IGHG*, *HLA*-*A/C/E*, *IL1RL1*, *CFD*, *LEF1*, *GPX3*) and oxidative stress (e.g., *GPX3*, *SHC1*) (Fig. 8C, 10.5281/zenodo.17793613 Tables 33–35).

**Fig. 9:**
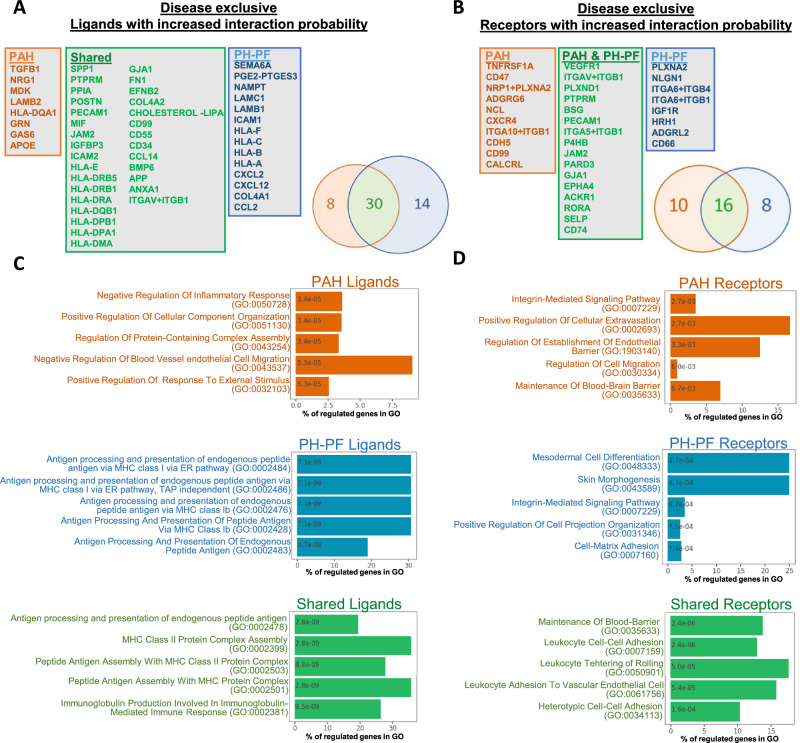
Ligand-receptor interactions in PAH and PH-PF have distinct fingerprints. **A** Venn diagram of ligands with increased interaction probability in pulmonary arterial hypertension (PAH, orange) and pulmonary hypertension with pulmonary fibrosis (PH-PF, blue) with the overlapping region showing shared ligands (green). **B** Venn diagram of receptors with increased interaction probability in PAH (orange) and PH-PF (blue) with the overlapping region showing shared receptors (green). **C** Bar graphs of top 5 gene ontologies (GOs) of upregulated ligands in PAH (top), PH-PF (middle) or PAH and PH-PF combined (bottom). *p* values are included in the bars. **D** Bar graphs of top 5 gene ontologies of up regulated receptors in PAH (up), PH-PF (middle) or shared (bottom). *p* values are included in the bars.

As these entity-driven transcriptomic changes could be highly relevant for targeted treatments, we subsequently analyzed disease-specific ligand–receptor interactions. Several PAEC ligands and receptors (10.5281/zenodo.17793613 Tables 36 and 37) exhibited disease-specific expression, pointing to divergent endothelial signaling programs in PAH and PH-PF. In PAH, upregulated genes encoding ligands, e.g., *TGFB1*, *NRG1*, *MDK*, *LAMB2* (Fig. 9A) and receptors, e.g., *TNFRSF1A*, *CD47*, *NCL*, *CXCR4*, *CDH5* (Fig. 9B) were linked to processes such as regulation of inflammatory responses and extravasation, EC migration, and barrier maintenance (Fig. 9C, D). In contrast, PH-PF showed unique gene expression upregulation of ligands, e.g., *SEMA6A*, *LAMC1*, *ICAM1*, *CXCL2*, *COL4A1* (Fig. 9A) and receptors, e.g., *PLXNA2*, *NLGN1*, *IGF1R, HRH1*, *CD66* (Fig. 9B) associated with antigen presentation and processing, immune cell-mediated cytotoxicity and cell-matrix interactions (Fig. 9C, D). The receptors and ligands regulated in both PAH and PH-PF, e.g., *MIF*, *CCL14, ICAM2, HLA-DRA, HLA-DRB1, HLA-DRB5* (Fig. 9A, B), were mostly involved in the regulation of inflammation (Fig. 9C, D, 10.5281/zenodo.17793613 Table 38). These differences indicate that endothelial communication networks are differentially altered across PH and may reflect disease-specific pathomechanisms depending on the underlying PH etiology.

## Discussion

Our study provides a comprehensive characterization of PAEC heterogeneity in health and disease (PAH and PH-PF), revealing three transcriptionally distinct subpopulations: immunomodulatory, vascular tone modulatory, and vascular plasticity modulatory PAECs. The identified subpopulations exhibit distinct functional implications and have been identified across multiple vascular beds, including the AO and CA, as well as in murine PAs. Our findings align with previous reports underscoring the high degree of EC heterogeneity in CAs and AOs^44,45^ and extend our understanding of the contribution of EC subtypes to pulmonary arterial health and disease.

Despite the three identified EC clusters being present within different arterial vascular beds, their relative abundance varies according to local physiological needs. In the pulmonary arterial endothelium, the clusters are present in roughly equal proportions under steady-state conditions, suggesting a stable functional equilibrium among the PAEC subtypes. In contrast, immunomodulatory ECs dominate in AO and CA, likely reflecting the heightened immune surveillance required in systemic circulation^46^. The proportion of vascular plasticity modulatory ECs (characterized by enrichment of genes involved in signal responsiveness, cytoskeletal remodeling, and environmental signal responsiveness) was independent of anatomical localization. The higher proportion of vascular tone modulatory ECs in the PAs may reflect the physiological need to adjust pulmonary blood flow to potential ventilation inhomogeneities. These patterns reveal that PAEC subpopulations are conserved yet adaptable, supporting variable needs for distinct vascular functions across different bodily contexts. This finding is further supported by our observation that transcripts previously associated with capillary or venous EC identity are also present in PAECs, suggesting that endothelial heterogeneity is driven by functional requirements.

For example, immunomodulatory PAECs express genes previously associated with venous endothelium (e.g., ACKR1), while vascular plasticity modulatory PAECs overlap with aCap and gCap markers (e.g., PRX)^36^. While we cannot completely exclude the presence of “contaminating” ECs of the capillary bed or vasa vasorum, particularly since vasa vasorum ECs express both VWF and ACKR1^47,48^, our immunofluorescence stainings clearly show the presence of, e.g., PRX-positive, ACKR1-positive or COL15A1-positive cells within ECs lining the PAs, indicating that there is indeed a spectrum of marker expression within the various EC compartments. Notably, immunomodulatory PAECs expressed PLVAP, a protein classically associated with endothelial fenestrations. While fenestrations are typically not observed in arterial endothelium, PLVAP expression in our dataset is consistent with publicly available human lung single-cell datasets demonstrating PLVAP expression across arterial, venous, and peribronchial/systemic endothelial populations^33^. In addition, PLVAP has been described not only as a structural component of fenestrated endothelia, but also as a regulator of vascular permeability and endothelial responses during inflammatory conditions, suggesting functional roles beyond classical fenestration-associated biology^49^.

As a consequence of their plasticity, endothelial phenotypes might be strongly prone to maladaptive reprogramming under pathological conditions (e.g., hypoxia, fibrosis, inflammation). Indeed, in PH, this transcriptional and proportional balance is disrupted, likely contributing to disease pathogenesis. The immunomodulatory PAEC cluster is drastically expanded in PAH and even more in PH-PF compared to donor PAs. This aligns with our and other’s findings regarding enhanced interaction of PAECs with immune cells and their mediators in PH^50^ and the accumulation of immune cells within the vessel wall^50,51^. Interestingly, in the murine model of hypoxia-induced PH, the immunomodulatory cluster was undetectable, while only vascular tone modulatory and vascular plasticity modulatory PAECs were present. This aligns with previous reports of hypoxia-induced immune suppression^52^ and substantiates not only disease-specific, but also species- and model-specific differences, reflecting increased complexity of human PH compared to purely hypoxia-induced PH in mice.

Importantly, our data do not suggest that the shift in PAEC subpopulations during PH is driven by changes in cell cycle, apoptosis, hyperproliferation, or senescence, but more likely by dynamic transcriptional reprogramming. In addition, none of the identified populations showed transcriptional signatures consistent with EndoMT. These findings suggest that in established human (end-stage) disease, vascular remodeling may be maintained through stable maladaptive cell states rather than ongoing cellular proliferation. Of note, we here analyzed PAs of small-to-mid size caliber (2–5 mm diameter), whereas intimal hyperplasia and proliferative remodeling in PH are most prominent in smaller distal arterioles (50–100 µm)^13^. Furthermore, as the analyzed samples were derived from end-stage human disease, it is likely that the major remodeling processes had already occurred, and the endothelial populations captured here may therefore reflect stabilized pathological states, rather than actively proliferating or transitioning PAECs. Consistently, not only the proportions of PAEC subtypes varied across different PH entities, but they also exhibited distinct transcriptional fingerprints. In PH-PF, genes related to angiogenesis (*TIMP3*, *TINAGL1, CCN1*), cell migration (*SPARC*, *FOXP1, BMP4, EDN1*) and cytoskeletal organization (*MYRIP*, *ACTB*) were dysregulated, pointing towards a shift from immunomodulatory to a pro-fibrotic functional profile. Among the genes strongly upregulated in PH-PF, phosphodiesterase 4B (PDE4B) is notable, as its selective inhibition has been reported to strengthen endothelial junctions, reduce adhesion protein expression^53^ and significantly slow fibrosis progression^54,55^. In PAH, by contrast, the immunomodulatory cluster was characterized by enrichment of genes involved in lipid and cholesterol metabolism, such as *APOE* and *APOC1*, and cellular migration. This suggests, that while PAECs of both PH entities engage migratory programs, lipid dysregulation may represent a defining feature of PAH-associated endothelial dysfunction.

A second commonality between PH-PF and PAH was the reduction of vascular plasticity modulatory PAECs. This reduction could either reflect endothelial damage due to sustained vascular pressure or constitute an early driver of disease progression by impairing mechanosensory and cell-cell signaling cascades. In PH-PF, this decline was accompanied by further suppression of antigen-presentation and cytotoxicity genes (e.g., *HLA-A, HLA-C*, and *HLA-E)*, hinting at a compromised ability to regulate inflammation. Notably, the upregulation of *LEF1*, an age-associated transcription factor linked to idiopathic pulmonary fibrosis (IPF)^56^ alongside *LTBP1*, a known activator of transforming growth factor *(*TGF)-β signaling^57^, points toward a transcriptional program that may directly contribute to fibrosis. In contrast, PAH vascular plasticity modulatory PAECs significantly upregulated *EDN1* and *SPP1*, two known factors promoting vasoconstriction and vascular remodeling^58,59^. These findings not only corroborate prior observations of altered endothelin-signaling in PAH-PAECs^32^, but refine them by localizing *EDN1* upregulation in PAH to specific subsets of PAECs (immunomodulatory and vascular plasticity modulatory), thereby offering a more accurate view of in situ endothelial dysfunction.

Lastly, the vascular tone modulatory cluster revealed divergent trajectories between the two disease states. In PAH, vascular tone modulatory PAECs were leaning toward a decreasing trend and displayed a strong transcriptional signature of disrupted lipid metabolism, reinforcing the concept of PAH-related metabolic dysfunction, particularly involving accumulation and impaired processing of lipids^60,61^. Moreover, the top regulated genes indicate attempts of endothelial regeneration, with increased expression of *PLK2* and *RSPO3*, which are both implicated in regulation of blood vessel development, stability and angiogenesis^62,63^, while expression of *EMP1*, a gene involved in regulating endothelial barrier properties^64^ and cellular proliferation^65^ was reduced. In contrast, the PH-PF transcriptomic profile of vascular tone-modulatory PAECs highlighted a pattern of increased cell-cell communication, ECM remodeling/adhesion, and immune responses, with increased expression of *CCL5*, *HMCN1* and *ITGAV*, all of which have been implicated in fibrotic remodeling processes in IPF^66–68^.

Taken together, these patterns indicate that while both PH-PF and PAH involve endothelial maladaptation, they diverge in the balance of fibrotic, metabolic, and vasoregulatory programs. The loss of vascular plasticity modulatory PAECs may increase endothelial mechanosensitivity and potentially elevate pulmonary pressures, thereby promoting vascular pathology through distinct but partially overlapping pathways. Divergent endothelial-mediated molecular pathomechanisms of PH-PF and PAH are supported by ligand–receptor interaction analysis, which highlights PAECs as distinct, multi-directional signaling hubs in the different disease entities. PAH-PAECs amplified communication networks are connected to the resolution of inflammation and vessel remodeling and to the maintenance of barrier integrity. In contrast, PH-PF PAECs intensified signals driving inflammation and ECM remodeling. This suggests that PH-PF is characterized by a persistent pro-inflammatory and pro-fibrotic PAEC state, while PAH-PAECs potentially engage compensatory or adaptive programs aimed at regulating inflammation, preserving barrier function and modulating vascular remodeling. However, these inferred interactions should be interpreted as hypothesis-generating and supportive rather than as direct experimental evidence, which is beyond the scope of this publication.

These novel insights have direct clinical implications: similar clinical phenotypes—elevated pulmonary pressures—can arise from fundamentally divergent endothelial pathomechanisms. Therefore, therapies designed for PAH, including vasodilators, may not address the underlying pro-fibrotic endothelial phenotype in PH-PF. Furthermore, genes such as *PDE4B* or *EDN1*, which are strongly upregulated in only one disease entity (PH-PF and PAH, respectively), also appear in the combined PH signature, underscoring the risk of overinterpreting seemingly shared molecular patterns. This may further explain ineffective interventions using most of the PAH medications in patients with PH-PF^69,70^. Thus, these findings highlight the need for precision medicine approaches and underscore the importance of stratifying PH subtypes when identifying and evaluating targeted treatments.

Collectively, our findings provide a comprehensive map of endothelial heterogeneity and disease-specific reprogramming in different PH-entities and a corresponding animal model. PAEC subpopulations may not merely be markers of cellular diversity but active drivers of disease progression through changes in abundance, transcriptional reprogramming, and adaptive intercellular signaling. While these changes foster a pro-fibrotic, pro-inflammatory endothelium in PH-PF, that destabilizes the ECM and disrupts vascular homeostasis, the PAH endothelial landscape is characterized by metabolic dysfunction with partial preservation of homeostatic and barrier-protective responses. These entity-specific endothelial transcriptomic signatures underscore the therapeutic relevance of endothelial diversity and advocate for refined patient stratification. By integrating single-cell transcriptomics, spatial mapping, and ligand–receptor analyses, our study highlights endothelial plasticity as a central determinant of vascular adaptation and maladaptation, providing a framework for disease-specific therapeutic targeting and paving the way toward precision medicine approaches in pulmonary vascular disease.

## Methods

### Study material

Human lung samples were collected at the Department of Thoracic Surgery, Medical University of Vienna, Vienna, Austria (lungs from patients with PH, and down-sized donor lungs), and University of Pennsylvania, Philadelphia, Pennsylvania, USA (non-utilized donor lungs)^37,38^. Lung tissue samples from donors and PH patients were processed using comparable procurement and handling protocols, and no systematic differences in ischemia time were reported. The scRNA-seq cohort comprised *n* = 5 donors, *n* = 3 PAH patients, and *n* = 3 PH-PF patients. The scRNA-seq cohort size was limited due to the restricted availability of isolated pulmonary arterial tissue obtained during lung transplantation. Independent patient cohorts were used for immunofluorescence validation experiments, with sample numbers reported per marker combination (overview scans: *n* = 2 per condition; isolated PA multiplex staining: *n* = 2; lung tissue multiplex staining: *n* = 3; initial dual-marker stainings per endothelial subpopulation: *n* = 3). Collection of tissue and clinical data was approved by the institutional ethics committee boards (Medical University of Vienna, Vienna, Austria, ethics numbers: EK 976/2010, EK 1417/2022 and EK 35-515 ex 22/23; Medical University of Graz EK 1291/2025 and EK 35-515 ex 22_23; University of Pennsylvania, Philadelphia, Pennsylvania, USA, ethics number: PROPEL 806345). Informed consent was provided to patients in accordance with the Declaration of Helsinki. All ethical regulations relevant to human research participants were followed. Demographical and clinical data of scRNAseq patient samples are shown in Table 1.

For animal PA data analysis, our publicly accessible superseries GSE228644 has been applied. The experimental set-up and sample acquisition have been described elsewhere^37^. In brief, male wild-type mice were housed for 3 weeks under normobaric hypoxia (10 O_2_) or normoxia (21% O_2_) conditions. The secondary and tertiary PAs were collected. Mechanical and enzymatic processing was performed as described for human PAs^37^. Acquisition of mouse samples were conducted in accordance with all relevant ethical regulations for animal use.

### PA isolation and PCLS preparation

All samples were processed within the timeframe of 6 months using a standardized procedure to avoid batch effects due to handling or processing of lung tissue. PAs were isolated from lungs of transplant patients and separately homogenized for 10x barcode labeling and library construction. Our scRNA-seq dataset includes PA samples from controls (*n* = 5), PH-PF (*n* = 3), and PAH (*n* = 3) patients^38^.

Human and murine PAs were isolated and processed for single-cell 10x Genomics capture according to the protocol described in Crnkovic et al.^37^, with additional details provided below. PAs with a diameter of 2–5 mm were identified under a stereomicroscope based on their close anatomical association with airways. Surrounding lung tissue was gradually removed to expose approximately 1–2 cm of the vessel, which was then excised as a single intact segment, including side branches, and processed for downstream analyses^51^.

PCLS cuts were procured by filling fresh lung pieces with 2.5% low melting agarose. A puncher was used to acquire 8 mm cylinders, which were then cut with the VF-210 (Precisionary instruments), into 300 µm thick slices that were subsequently used for tissue clearing^71^.

### Tissue clearing and 3D immunofluorescence imaging

PCLS were fixed, washed and incubated with FLASH^72^ reagent 1 (4% wt/vol SDS, 200 mM borate) for 16 h at 42 °C. Following treatment with blocking solution (10% wt/vol FBS, 0.02% sodium azide, 1% wt/vol BSA and 5% vol/vol DMSO in PBT, 0.2% vol/vol Triton X-100 in PBS), samples were incubated with primary antibodies (Table 2) overnight and with the corresponding secondary antibodies also overnight at 4 °C. Finally, samples were dehydrated in four steps with 100%, 75%, 50% and 30% solution of methanol (diluted in PBS) and then cleared with four increasing concentrations of BABB solution (33.3% vol/vol benzyl alcohol, 66.7% vol/vol benzyl benzoate). After tissue clearing, the imaging chamber was mounted using a glass bottom dish (GWST-3522, Willco Wells), the sample was placed in the middle of the glass bottom chamber and three silicon spacers were applied on the edge of the bottom of the chamber. To avoid sample movement during scanning, a round cover glass was applied with a small pressure on top of the sample. Finally, the chamber was filled with BABB solution to enable refractive image matching and subsequent in-depth imaging of the specimen. Images were obtained with the Leica TCS-SP8 Lightning confocal microscope (Leica) and processed using LAS-X Software for 3D rendering.

### Multicolor fluorescence imaging

Human lung samples were fixed with 4% buffered formaldehyde, dehydrated, and paraffin-embedded. Sections (2.5 µm) were deparaffinized and subjected to heat-induced antigen retrieval in retrieval solution pH = 6 (Perkin-Elmer), followed by blocking with antibody blocking solution (Perkin-Elmer) for 20 min at room temperature. Primary antibodies (Table 2) diluted in antibody diluent (Perkin-Elmer) were sequentially incubated overnight at 4 °C. Between the incubation steps, slides were washed with TBS-T buffer (20 mM Tris Base, 150 mM NaCl, 0.1% Tween20 in PBS, pH = 7.4). Antigen detection with the Opal kit (Perkin-Elmer) was performed according to the manufacturer’s instructions. In brief, tissue sections were incubated with Opal Polymer HRP mouse/rabbit/rat secondary antibody and developed with Opal fluorescent substrates. Antigen detection with secondary antibodies was performed by incubating the primary antibodies overnight at 4 °C, followed by incubation with corresponding, fluorescent-labeled secondary antibodies for 2 h at room temperature. Nuclear counterstaining was performed using 1 mg/ml DAPI solution (Thermo Scientific). Images were obtained with the Leica TCS-SP8 Lightning confocal microscope (Leica) and processed using LAS-X Software or with the Olympus VS200 and processed with QuPath.

### Statistics and reproducibility

#### Pre-processing

Pre-processing of the raw scRNA data was performed with 10x Cell Ranger. Raw sequencing files were demultiplexed and aligned against the human reference genome GRCh38. Data processing was carried out using the R-based package Seurat 5^73^.

Each sample (Donor *n* = 5, PAH *n* = 3, PH-PF *n* = 3) was processed individually and subsequently concatenated into a combined dataset for joint analysis. To account for potential batch effects arising from differences in sample acquisition and processing time, we applied established integration methods. Canonical correlation analysis (CCA)–based integration was used for gene expression–focused analyses, while Harmony was applied for trajectory inference. The performance of both integration approaches was quantitatively evaluated using the Local Inverse Simpson Index (LISI) (10.5281/zenodo.17793613 Table 39), demonstrating effective mixing of samples while preserving biological structure and cell type identity.

#### Quality control and initialization

The quality control was performed by filtering out low-quality cells, empty droplets, and multiplets. For this purpose, filtering was applied during alignment to ensure that empty cells are excluded. Furthermore, we filtered out cells with mitochondrial genes above 10% or cells with gene reads above 4000 or below 200. This ensures that almost no multiplets are influencing the analysis. Variable genes were identified with the Seurat FindVariableFeatures method with “vst” selection method with the number of variable features as top variable features set to 2000. The Seurat ScaleData function was used to scale and center expression values in the data set for dimensional reduction. Principal component analysis (PCA) for dimensional reduction was performed with the Seurat command RunPCA based on the variable genes previously identified. Cell clustering was achieved by, firstly, constructing a shared nearest neighbor graph based on the Euclidean distance in Principal component analysis using Seurat command FindNeighbors and, secondly, applying modularity optimization technique Louvain algorithm, implemented in the Seurat command FindClusters (resolution = 2). Further dimensionality reduction was performed using uniform manifold approximation and projection for dimensional reduction (UMAP) algorithm based on the top 20 significant principal components (PCs). Dimensionality reduction and clustering based on major cell population markers (Table 1) led to the identification of the immune, mesenchymal and EC compartments (Supplementary Fig. 1A). Based on canonical cell type markers listed in the Zenodo dataset 10.5281/zenodo.17793613 Table 2, the compartments were further subdivided into 15 clusters in all three lung phenotype groups (Supplementary Fig. 1B). Epithelial cell abundance was used as a substitute indicator of possible parenchymal contamination (Supplementary Fig. 13). For each sample, the fraction of epithelial cells was quantified and treated as a conservative upper-bound estimate of potential parenchymal carryover. Associations between epithelial fraction and endothelial subcluster composition were assessed on a per-sample basis. To account for potential batch, we applied established batch correction methods, including canonical correlation analysis (CCA) and Harmony.

Clusters were evaluated for potential endothelial-to-mesenchymal transition (EndoMT)-like characteristics based on their transcription of EndoMT associated genes (10.5281/zenodo.17793613 Table 39) utilizing Seurat’s AddModuleScore function^73^.

Trajectory inference of pulmonary arterial endothelial cells (PAECs) was performed using Monocle3 on a Harmony integrated UMAP embedding. PCA was computed on scaled gene expression data and batch-corrected using Harmony with sample identity as a covariate, followed by UMAP dimensionality reduction. Monocle3 was used to learn a principal graph capturing the global structure of the PAEC population. A root node was manually specified at the bottom left region of the UMAP based on marker expression and spatial positioning. Pseudotime was calculated as the geodesic distance along the inferred graph from this root, with increasing values indicating progression along the proposed trajectory.

In order to recognize our EC subpopulations in other compartments (AO, CA) or organisms (mouse) we used the top 30 genes for each clusters’ differential expression analysis between all PAEC samples and calculated a correlation score utilizing Seurats AddModuleScore function. Cluster-specific DEGs were identified by performing the MAST^74^ test via the Seurat package (version 5.0) using the function FindAllMarkers with Bonferroni correction and a threshold of adjusted *p* value of 0.05, log2 fold change of ±0.5, a minimum of 20% expressing cells in the target population and a maximum of 20% in remaining cells. DEGs between donor and PH samples were ascertained in the same way, but with the FindMarkers function instead. Disease-specific (Donor versus PAH, Donor versus PH-PF) fingerprint genes were computed with the same parameters, except for the 20% thresholds. The R-based package “CellChat”^75^ was used to determine ligand-receptor interactions, which utilizes a curated databank based on KEGG and performs calculations with Wilcoxon rank sum test. To improve readability, CellChat was set to only show the top 20% interactions. Enrichment analysis was performed with “Enrichr”^76^ using Fisher’s exact test, followed by Benjamini-Hochberg correction with an adjusted *p* value threshold of 0.05. Cell-cycle phase scoring was performed via inferring specific scores based on a list of G2M- and S-specific genes, described in Tirosh et al.^77^ and subtracting the aggregated expression of a randomized control gene set. Statistical population analysis was performed on a per-sample basis. Percentages of cells assigned to each endothelial archetype and the median and interquartile range (IQR) were calculated across samples for each compartment (AO/CA/PA), diagnosis (Donor/PAH/PHPF), or cell cycle status (S/G2M/G1). Total cell numbers and cluster representation per sample, demonstrating that no individual sample disproportionately influenced clustering, are provided in Supplementary Fig. 13. This per-sample approach ensures equal weighting of biological replicates and prevents disproportionate influence of samples with large cell numbers, with wider IQRs reflecting true inter-sample biological heterogeneity rather than technical pooling effects. Statistical comparisons of endothelial cluster distributions were performed on a per-sample basis using two-sided non-parametric tests. Differences between more than two groups were assessed using the Kruskal–Wallis test, followed by pairwise Wilcoxon rank-sum tests with Holm correction where applicable. Effect sizes were calculated as epsilon squared (*ε*²) for Kruskal–Wallis tests and as rank-biserial correlation (*r*) for Wilcoxon tests using the *rstatix* package. Exact *p* values are reported. Full statistical details are provided in the corresponding supplementary data. Of note, surfactant protein C (*SFTPC*) was excluded from the analysis and visualization since its expression pattern in certain samples was deemed stemming from ambient RNA and is therefore treated as a technical artifact. It was not excluded from the zenodo tables (10.5281/zenodo.17793613) that contain log fold changes and *p* values for interested parties. Additional visualization packages were used, namely: scCustomize^78^ for better feature plot readability and Scillus^79^ for heat map generation. We utilized the marker set from Tirosh et al. to check for cell cycle progression and the SenMayo Gene set for apoptosis and scenescence.^80,81^

### Use of artificial intelligence tools

Artificial intelligence (AI) based models were utilized during manuscript preparation for linguistic refinement. No AI tools were used for data generation, data analysis or interpretation of results. All outputs were critically reviewed and verified by the authors.

### Reporting summary

Further information on research design is available in the Nature Portfolio Reporting Summary linked to this article.

## Supplementary information

Supplementary Information

Reporting Summary

Transparent Peer Review file

## Data Availability

All human and mouse pulmonary artery single-cell RNA sequencing (scRNA-seq) data are available in the Gene Expression Omnibus (GEO)^82^ under the superseries accession GSE228644. Publicly available datasets GSE155468 (aortic data) and GSE131778 (coronary artery data) were obtained from GEO as provided by the original authors. Supplementary data can be accessed via Zenodo^83^: 10.5281/zenodo.17793613. All data can be accessed through the repository accession numbers and DOI link provided above. All other data supporting the findings of this study are available from the corresponding author upon reasonable request.

## Code availability

Custom code used for data processing, analysis, and visualization is available via Zenodo at 10.5281/zenodo.17793613. The repository includes R scripts and a directly usable “.rds” file required to reproduce the analyses presented in this study. Analyses were performed using R (version 4.4.1) and primarily relied on the Seurat package (version 5.0) for single-cell data processing and analysis. Additional packages used include Harmony for batch correction, Monocle3 for trajectory inference, CellChat for ligand–receptor interaction analysis, Enrichr for enrichment analysis, and rstatix for statistical testing. Visualization was supported by scCustomize and Scillus. Package versions and relevant parameters are documented within the provided code.
