# Supplementary material for: Transcriptional landscape of pulmonary artery endothelium reveals subpopulation- and disease-specific remodeling signatures
Source: Commun Biol. 2026 May 11;9:998. doi: 10.1038/s42003-026-10204-0 (PMC13385408; doi:10.1038/s42003-026-10204-0)
Supplement: Supplementary file 1 — Supplementary Information [file 42003_2026_10204_MOESM1_ESM.pdf]

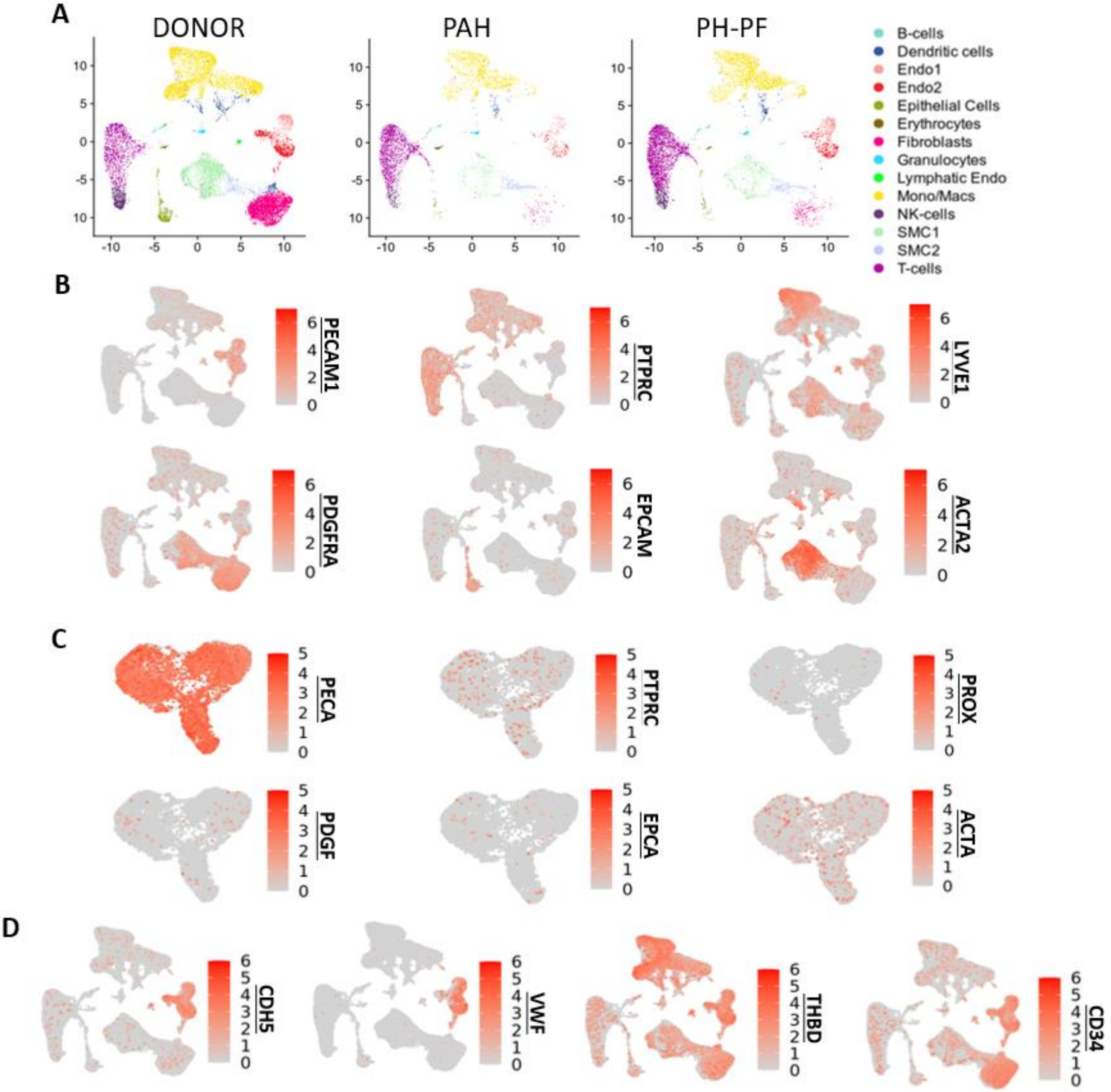

Supplementary Figure 1: Identification and subsetting of endothelial cells

(A) Uniform manifold approximation and projection (UMAP) plots depicting, immune, fibroblast-, lymphatic-, smooth muscle- and endothelial cells (ECs) in the full scRNA-seq dataset of human donor (n=5), pulmonary arterial hypertension (PAH, n=3) and pulmonary hypertension with pulmonary fibrosis (PH-PF, n =3) lungs . (B) Feature plots showing marker genes for ECs (PECAM1), immune cells (PTPRC), lymphatic ECs (LYVE1), fibroblasts (PDGFRA), epithelial cells (EPCAM1) and smooth muscle cells (ACTA2) in the full scRNA seq dataset of human donor, PAH and PH-PF lungs. (C) Feature plots of the endothelial population showing marker genes ECs (PECAM1), immune cells (PTPRC), lymphatic ECs (LYVE1), fibroblasts (PDGFRA), epithelial cells (EPCAM1) and smooth muscle cell (ACTA2). in the endothelial population of human donor, PAH and PH-PF lungs. (D) Feature plots showing EC marker genes (CDH5, VWF, THBD, CD34) in the full scRNA-seq dataset of human Ddonor, PAH and PH-PF lungs. Color gradients represent different levels of expression strength per cell; grey cells have expression below a cut off of 1e-09.

A

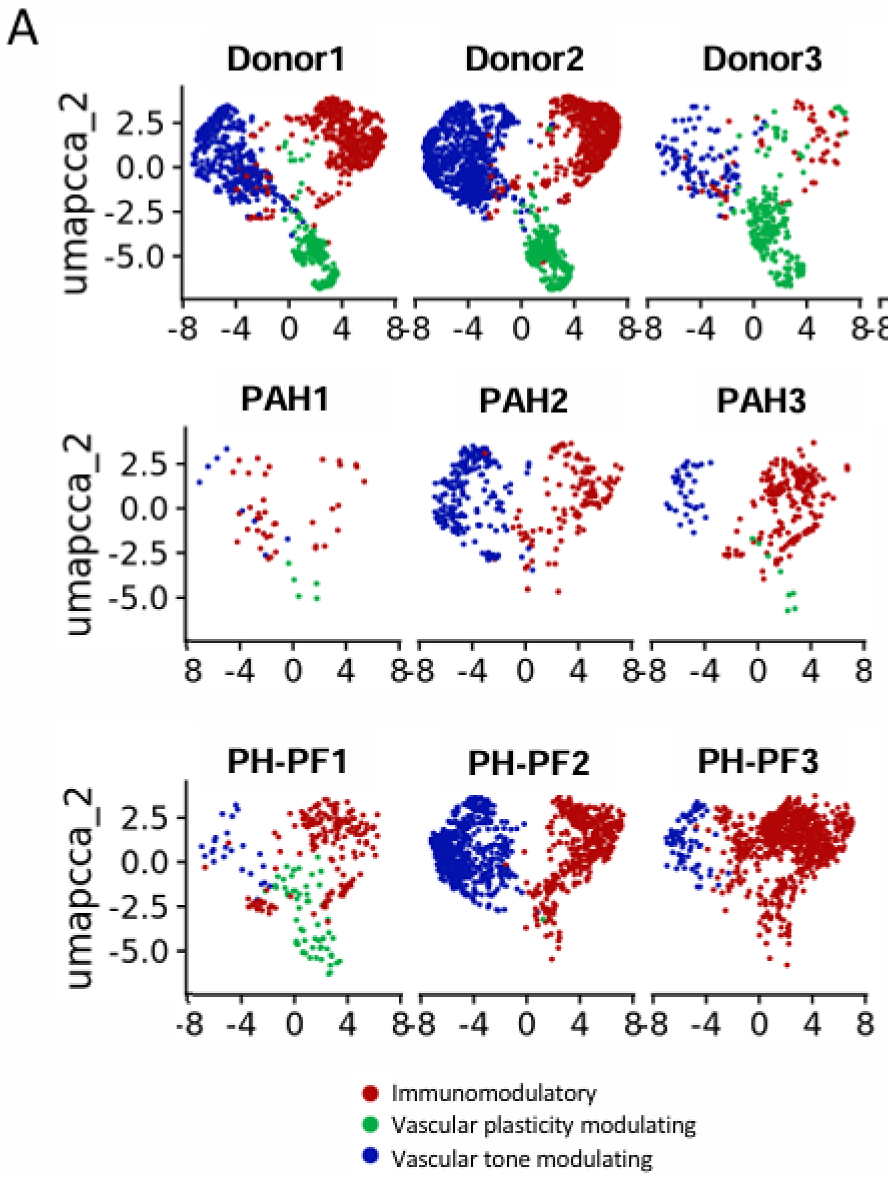

B

Sample	Count
Donor1	941
Donor2	1643
Donor3	381
Donor4	46
Donor5	968
PAH1	51
PAH2	311
PAH3	238
PH-PF1	263
PH-PF2	946
PH-PF3	846

Supplementary Figure 2: UMAP projections of modulatory endothelial subpopulations across individual donors and single PAH samples

(A) Uniform manifold projection (UMAP) embeddings for each individual donor (Donor1–Donor5) and each pulmonary hypertension (PH) sample (PAH1–PAH4, PH-PF1– PH-PF3). Cells are colored by functional annotation: immunomodulatory (red), vascular plasticity modulatory (green), and vascular tone modulatory (blue), across individual donors and PH samples. (B) Table of individual pulmonary arterial endothelial cell (PAEC) counts per sample.

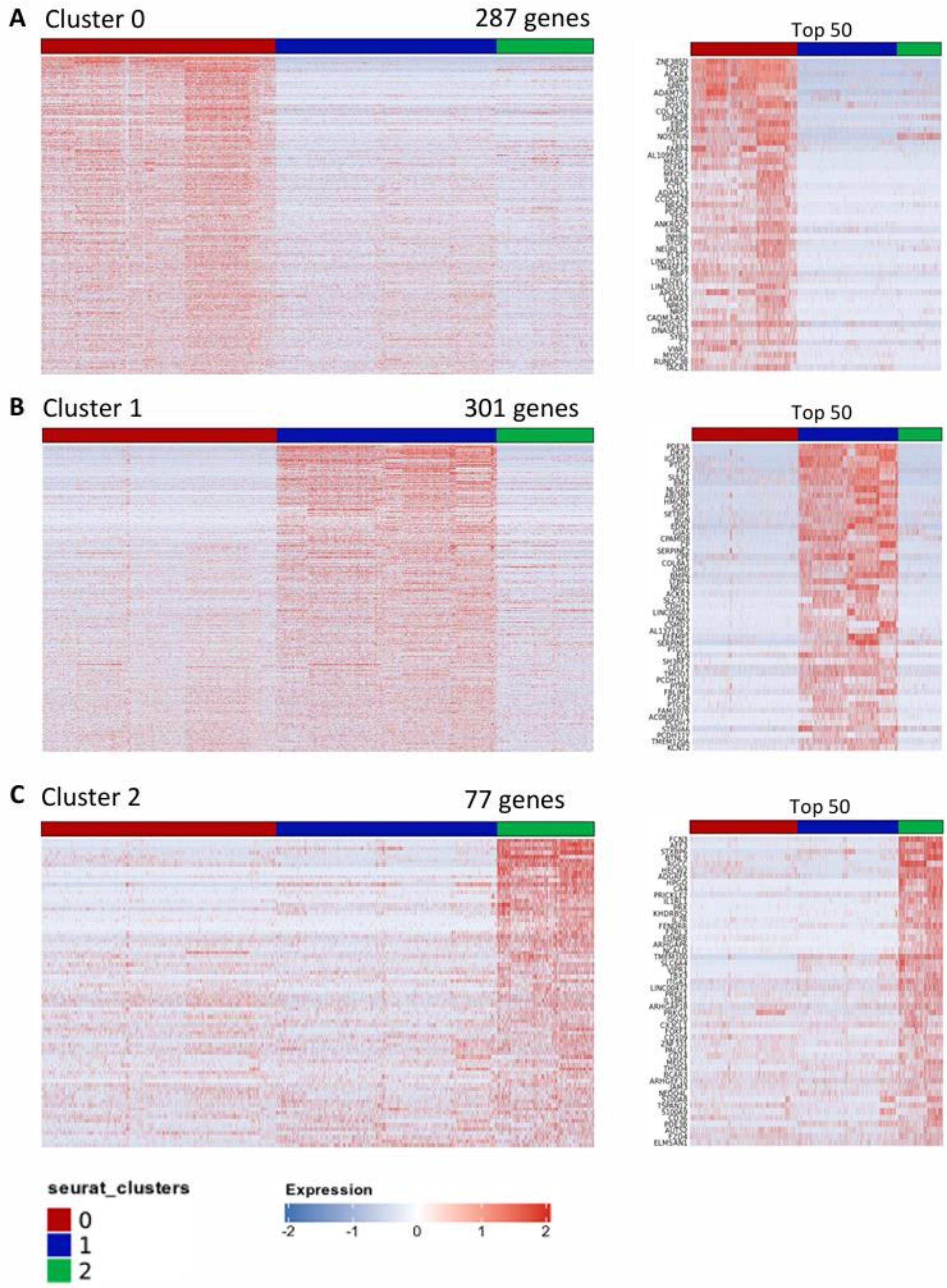

Supplementary Figure 3: Pulmonary artery endothelial cells show 3 distinct subtypes in functionality.

Heatmaps of the endothelial population extracted from the full scRNA-seq dataset of human donor (n=5), pulmonary arterial hypertension (PAH, n=3) and pulmonary hypertension with pulmonary fibrosis (PH-PF, n =3) lungs showing differential expression of genes specifically enriched in (A) cluster 0 (immunomodulatory), (B) cluster 1 (vascular tone modulatory) or (C) cluster 2 (vascular plasticity modulatory). Genes were selected by an adjusted p-value below 0.05, an average log fold change > 0.5, a minimum of 20% of cells expressing the gene within the target population and a maximum of 20% of cells expressing the gene outside of the target population.

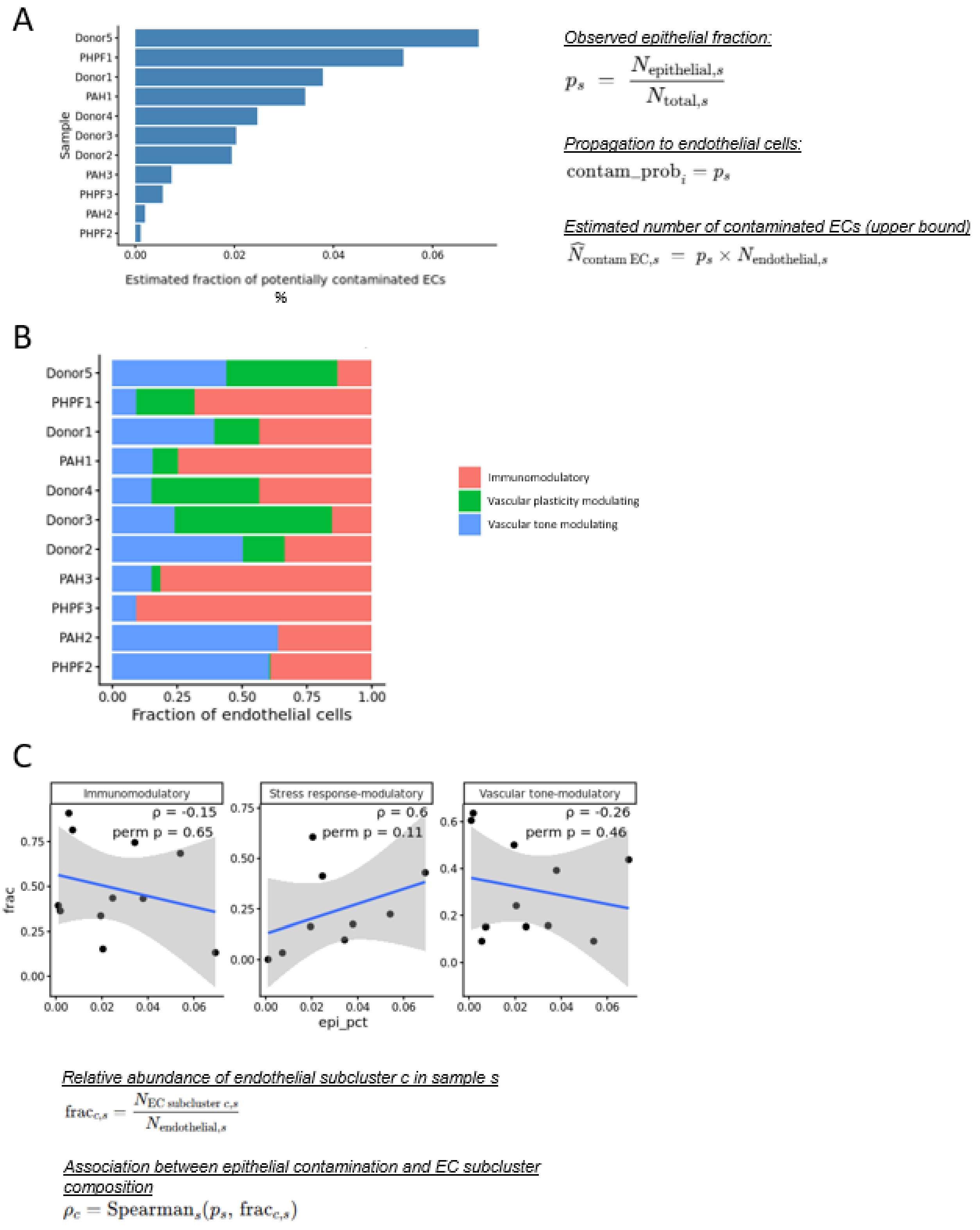

Supplementary Figure 4: Potential parenchymal contamination shows negligable impact on analysis .

(A) Bargraph and calculation formula of estimated fraction of potentially contaminated endothelial cells per sample; the dashed line indicates a 10% threshold. (B) Stacked bargraph of relative cell counts of functionally annotated endothelial subclusters per sample. (C) Association between epithelial contamination and endothelial subcluster composition, quantified as the fraction of endothelial cells belonging to each subcluster and assessed using Spearman rank correlation across samples as indicated in the formula. Shaded areas indicate 95% confidence intervals, and permutationbased p-values are shown.

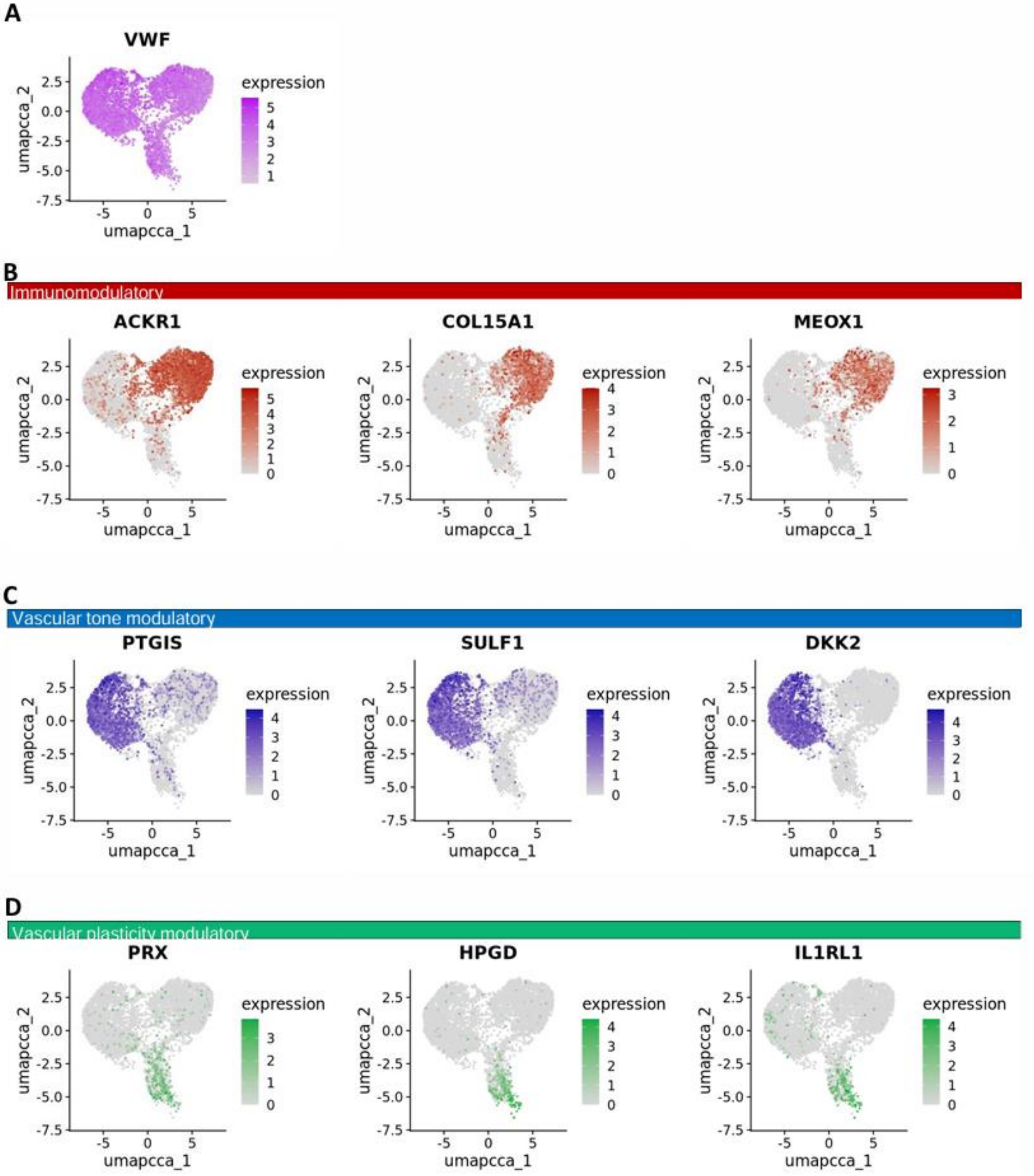

Supplementary Figure 5: UMAP visualization of a vWF-positive endothelial cell subset

(A) Feature plot showing vWF expressing endothelial cells (ECs) extracted from the endothelial population of the human scRNA-seq dataset of human donor (n=5), pulmonary arterial hypertension (PAH, n=3) and pulmonary hypertension with pulmonary fibrosis (PH-PF, n =3) lungs. (B) Feature plots of the selected marker genes for the immunomodulatory cluster (ACKR1, COL15A1, MEOX1) in the extracted EC population of human donor, PAH and PH-PF lungs. (C) Feature plots of the selected marker genes for the vascular tone modulatory cluster (PTGIS, SULF1, DKK2) in the extracted EC population of human donor, PAH and PH-PF lungs. (D) Feature plots of the selected marker genes for the vascular plasticity modulatory cluster (PRX, HPGD, IL1RL1) in the extracted EC population of human donor, PAH and PH-PF lungs. Color gradients represent different levels of expression strength per cell, grey cells have expression below a cutoff of 1e-09.

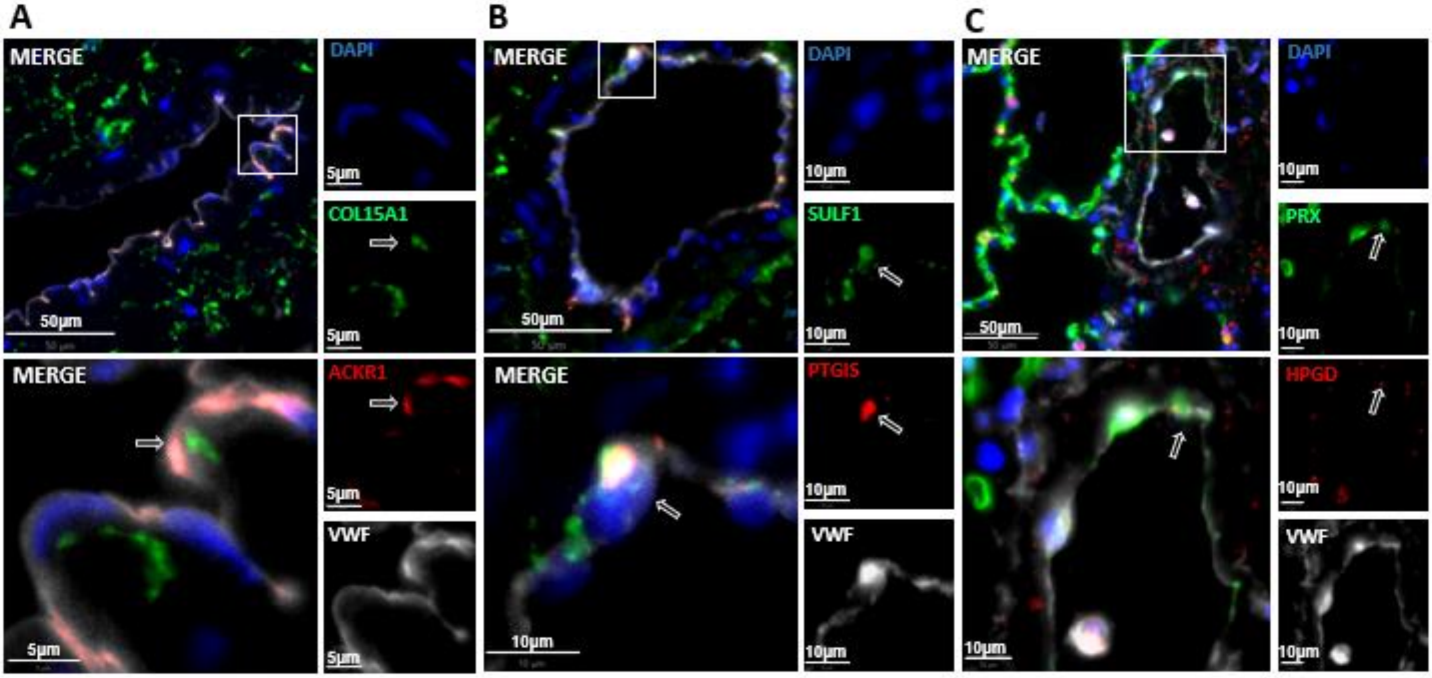

Supplementary Figure 6: Validation of endothelial subpopulation marker expression in pulmonary arteries by dual immunofluorescence staining

Representative immunofluorescence images of human formalin fixed paraffin embedded donor lung tissue sections (n=2). In each panel, the top left image shows a merged overview, with the remaining images displaying higher magnification views and corresponding single channel stainings of (A) COL15A1 (green) and ACKR1 (red), (B) SULF1 (green) and PTGIS (red) and (C) PRX (green) and HPGD (red). All panels include co-staining with vWF (grey) (endothelial marker) and DAPI (blue) (nuclei). Arrows indicate marker positive endothelial cells.

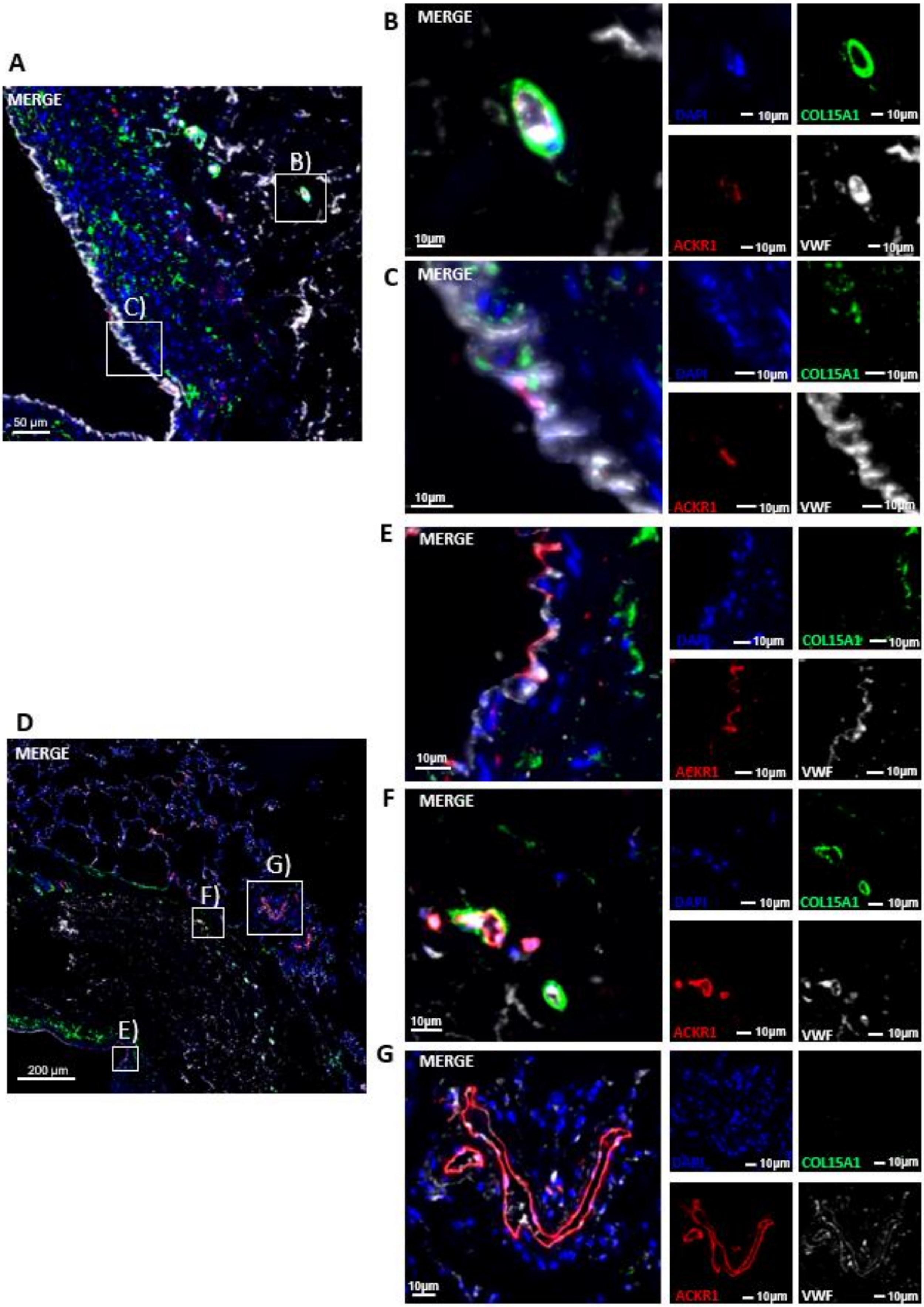

Supplementary Figure 7: In situ localization of COL15A1 and ACKR1 protein.

(A, D) Representative immunofluorescence images of human formalin fixed paraffin embedded donor lung tissue sections (n=3) stained against ACKR1 (red), COL15A1 (green) and VWF (grey). DAPI was used as nuclear counterstain. The white boxex represent the zoom areas specifically showing small peribronchial/perivascular vessels (BF), pulmonary arteries (CE) and pulmonary veins (G).

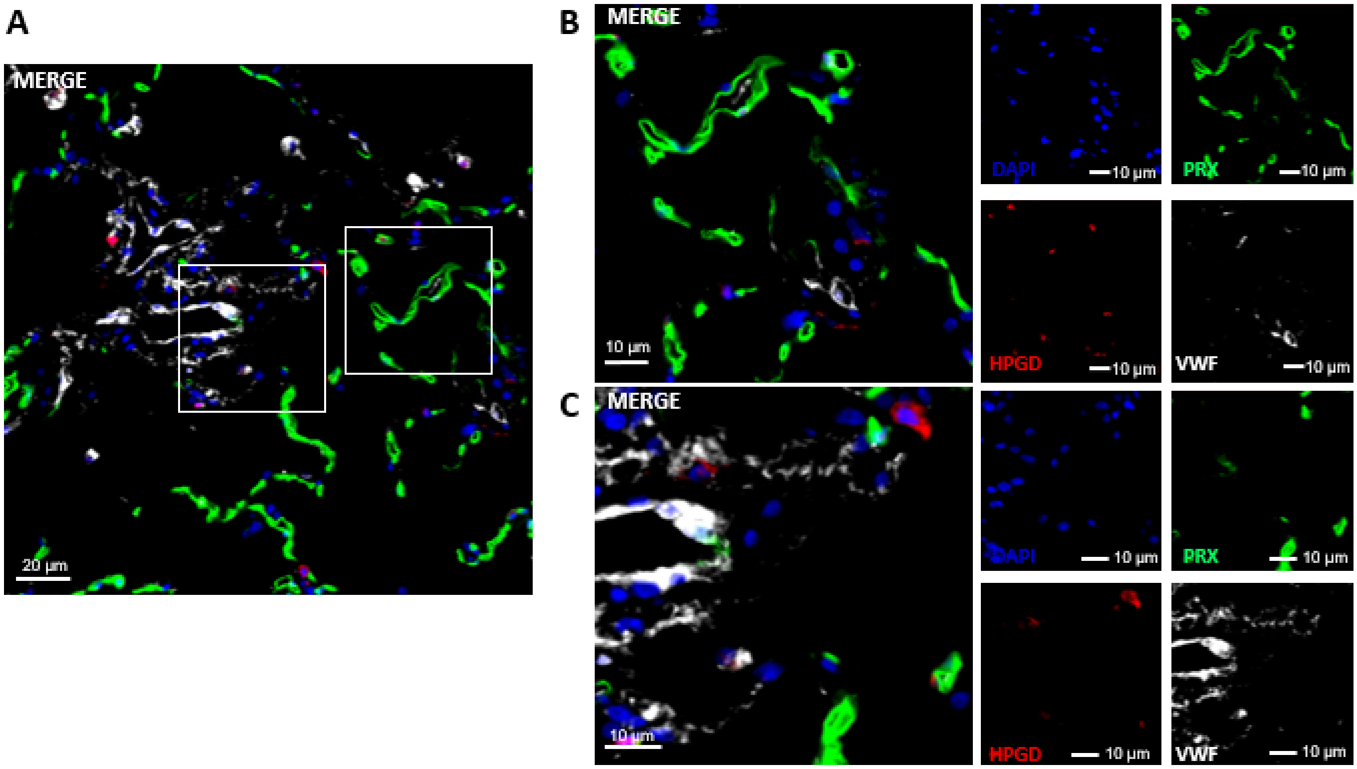

Supplementary Figure 8: In situ localization of PRX and HPGD protein.

(A) Representative immunofluorescence images of human formalin fixed paraffin embedded donor lung tissue sections (n=3) stained against PRX (green), HPGD (red) and VWF (grey). DAPI was used as nuclear counterstain. White boxes represents the zoom areas specifically showing pulmonary capillaries (B) and pulmonary arteries (C).

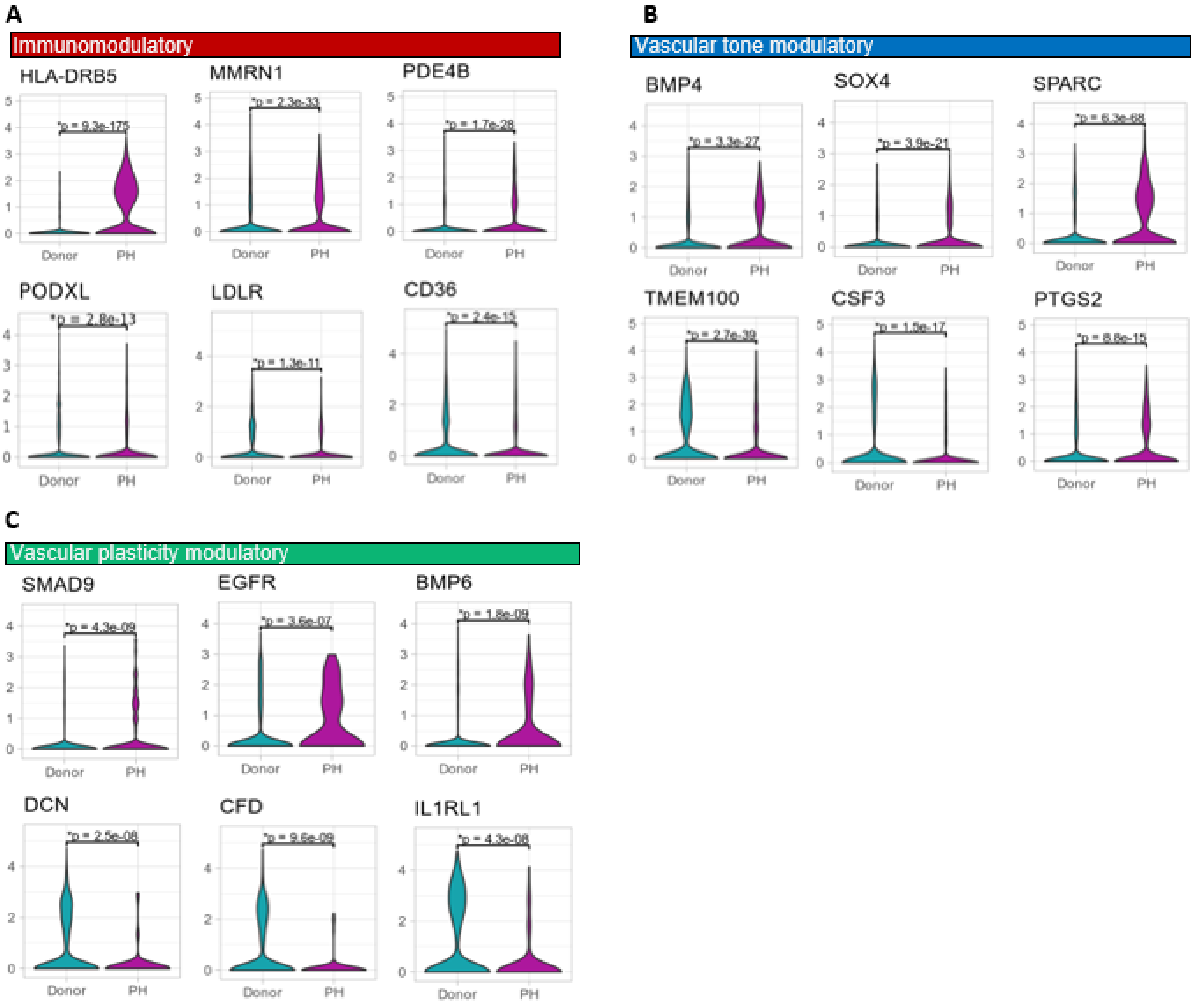

Supplementary Figure 9: Gene specific changes within pulmonary artery endothelial cell subpopulations

(A) Violin plots of selected genes of interest of the immunomodulatory cluster: HLA-DRB5 ($p = 9.3\times10^{-175}$), MMRN1($p=2.3\times10^{-33}$),PDE4B ($p = 1.7\times10^{-28}$), PODXL ($p = 2.8\times10^{-13}$), LDLR ($p = 1.3\times10^{-11}$) and CD36 ($p = 2.4\times10^{-15}$). (B) Violin plots of selected genes of interest of the vascular tone modulatory cluster: BMP4 ($p = 3.3\times10^{-27}$), PTGS2 ($p = 8.8\times10^{-15}$), SPARC ($p = 6.3\times10^{-68}$), CSF3 ($p = 1.5\times10^{-17}$), SOX4 ($p = 3.9\times10^{-21}$) and TMEM100 ($p = 2.7\times10^{-39}$). (C) Violin plots of selected genes of interest of the vascular plasticity modulatory cluster: SMAD9 ($p = 4.3\times10^{-9}$), EGFR ($p = 3.6\times10^{-7}$), BMP9 ($p = 1.8\times10^{-9}$), DCN ($p = 2.5\times10^{-8}$), CFD ($p = 9.6\times10^{-9}$) and IL1RL1 ($p = 4.3\times10^{-8}$). p-values were calculated with Wilcoxon rank sum test.

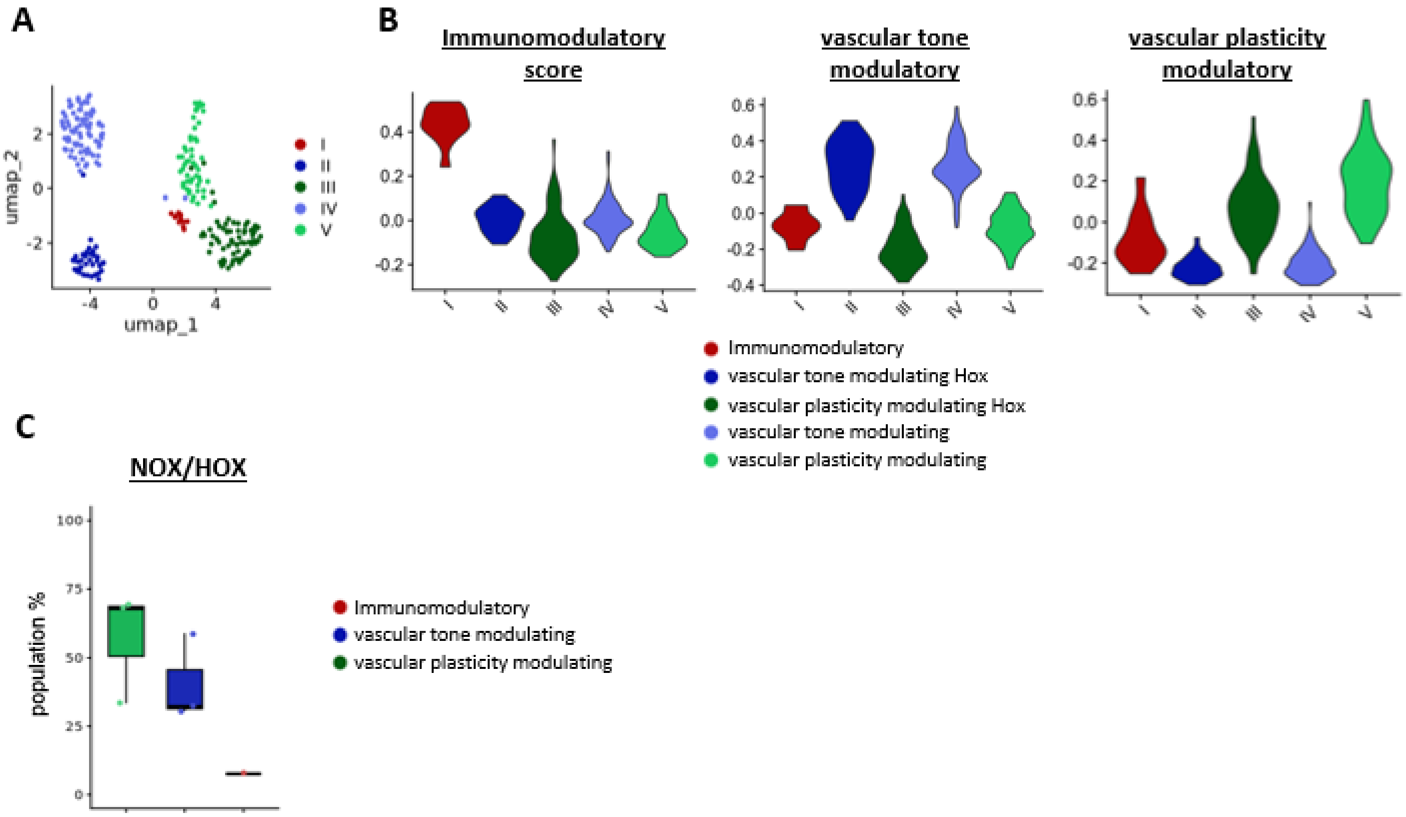

Supplementary Figure 10: Endothelial heterogeneity and distribution across different organisms.

(A) Uniform manifold approximation and projection (UMAP) plot depicting the subclustered endothelial cell population extracted from the pulmonary artery scRNA-seq dataset of normoxia and hypoxia treated mice (n=3). (B) Violin plots of endothelial cell (EC) subpopulation correlation scores calculated from the top 30 differentiating marker genes per cluster of pulmonary arteries (PA) of normoxia or hypoxia treated mice. (C) Box Plots depicting sub cluster EC population proportions in the PAs of normoxia and hypoxia treated mice. Proportions are shown as mediansand interquartile range.

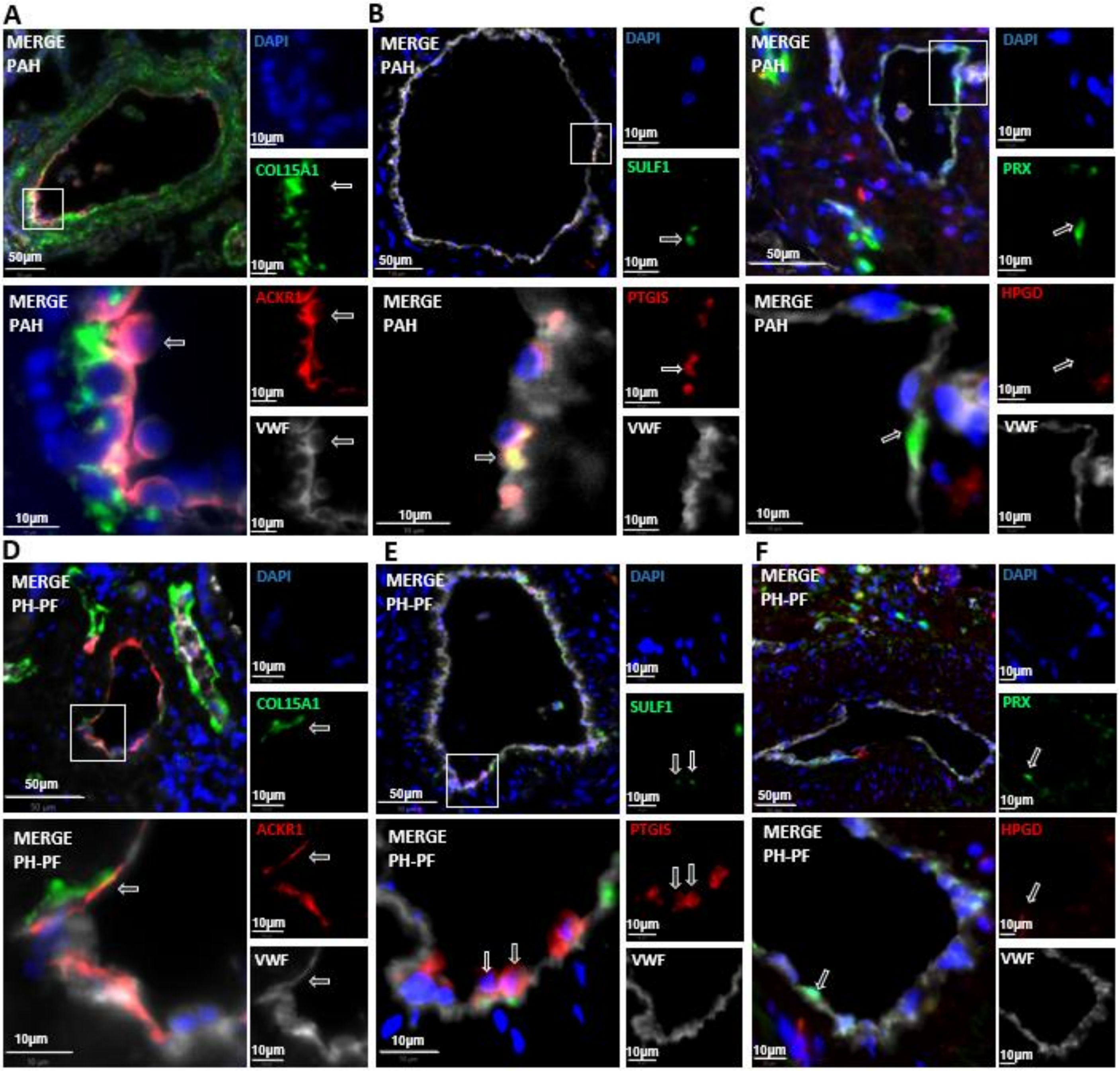

Supplementary Figure 11. Dual immunofluorescence staining confirms endothelial subpopulation marker expression in PAH and PH-PF pulmonary arteries

(A–C) Dual immunofluorescence staining of formalin-fixed paraffin-embedded (FFPE) lung tissue sections from patients with pulmonary arterial hypertension (PAH) demonstrating endothelial cells expressing markers of the immunomodulatory (COL15A1green /ACKR1 red), vascular tone–modulatory (SULF1 green /PTGIS red), and plasticity modulatory (PRX green /HPGD red) subpopulations. (D–F) Corresponding dual stainings performed on FFPE lung tissue sections from patients with pulmonary hypertension associated with pulmonary fibrosis (PH-PF). In each panel, merged overview images are shown together with higher magnification views (areas indicated in white boxes) and corresponding single channel stainings for DAPI (nuclei) and vWF (endothelial marker). Arrows indicate marker positive endothelial cells along the vascular intima.

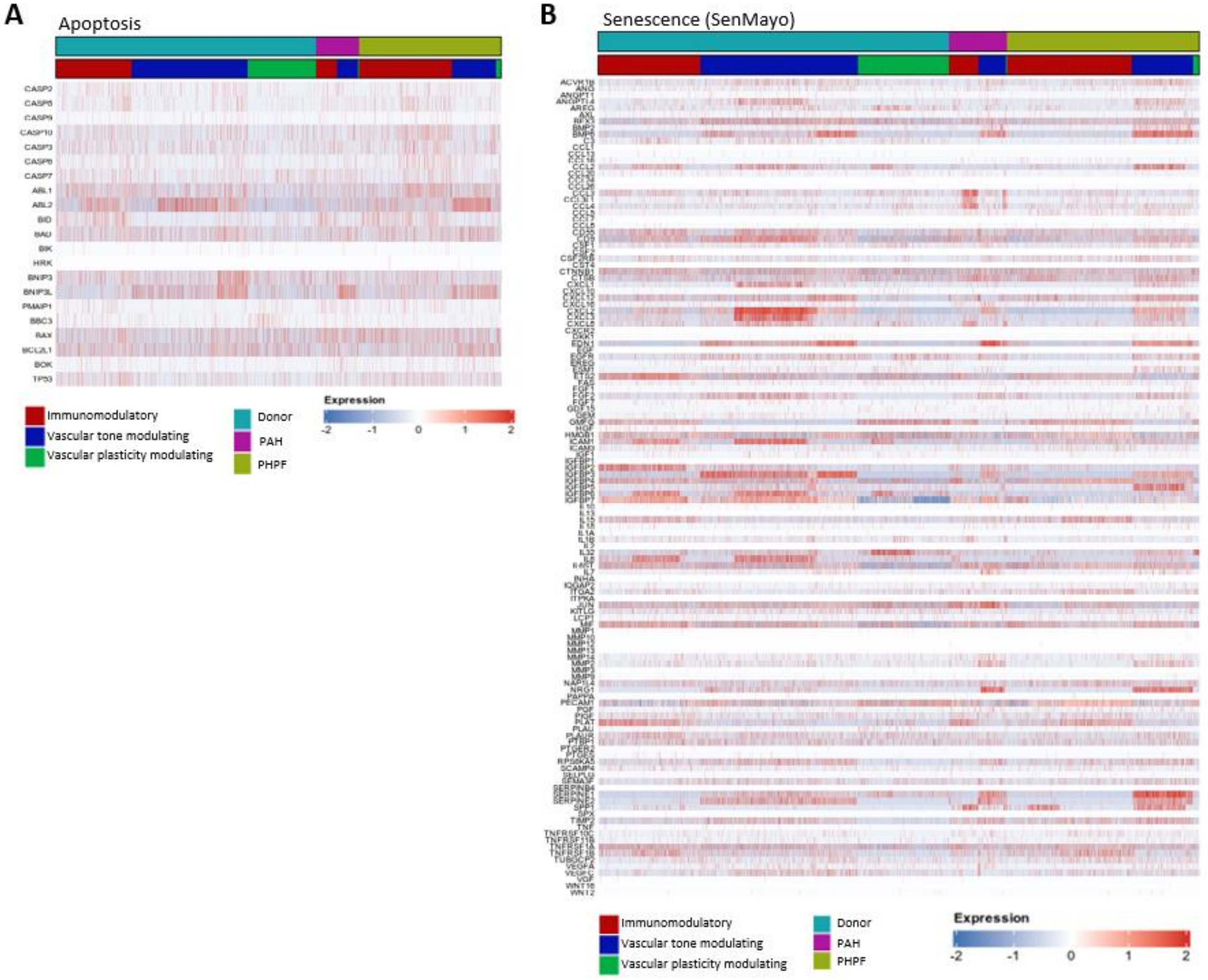

Supplementary Figure 12: PAEC subpopulations give no indication of regulatory changes derived from apoptosis or senescence.

(A) Heatmap of selected apoptosis genes expressed in the immunomodulatory, vascular tone modulatory, vascular plasticity modulatory endothelial subpopulations extracted from the full scRNA-seq dataset of human donor (n=5), pulmonary arterial hypertension (PAH, n=3) and pulmonary hypertension with pulmonary fibrosis (PH-PF, n =3) lungs. (B) Heatmap of SenMayo senescence genes expressed in the immunomodulatory, vascular tone modulatory and vascular plasticity modulatory endothelial subpopulations extracted from the full scRNA seq dataset of human donor, PAH and PH-PF lungs. The color gradient represents scaled gene expression relative to the depicted genes.

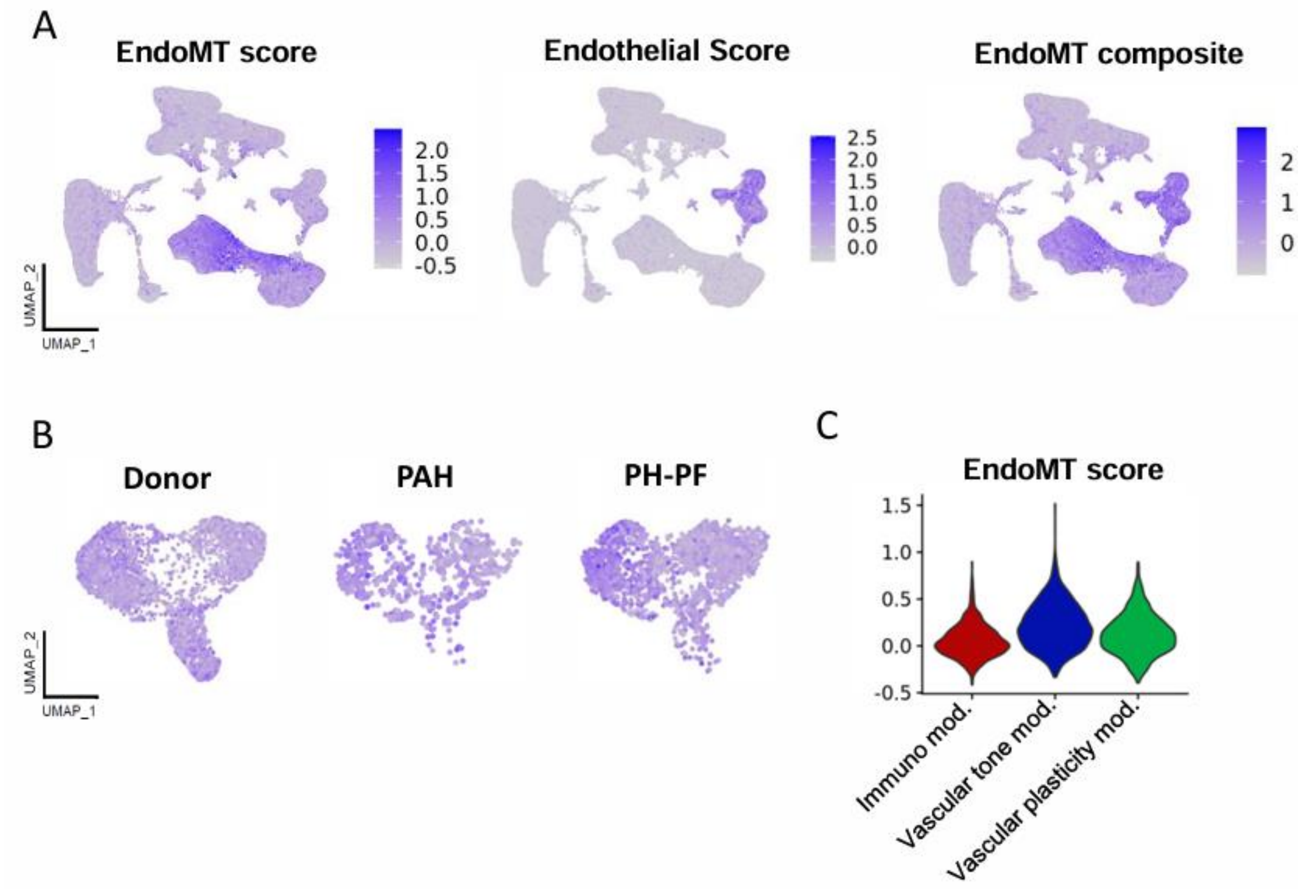

Supplementary Figure 13. Endothelial-to-mesenchymal transition (EndoMT) assessment identifies an ambiguous, donor-enriched endothelial subpopulation lacking EndoMT identity

(A) Module scores for mesenchymal/EndoMT-associated genes (VIM, TAGLN, ACTA2, CNN1, COL1A1, COL1A2, COL3A1, FN1, SPARC, SERPINE1, SNAI1, TWIST1, ZEB1, CTGF, SMAD3, CDH2), endothelial identity genes (PECAM1, VWF, KDR, CDH5, ESAM, RAMP2, EMCN), and a composite EndoMT score (sum of mesenchymal and endothelial module scores) projected onto the integrated uniform manifold projection (UMAP) of the full dataset. (B) UMAP of composite EndoMT scores stratified by disease condition (donor, PAH, PH-PF) within the endothelial cell (EC) subpopulations. (C) Violin plots of EndoMT module scores among the EC subpopulations.
