# Supplementary material for: Transcriptional landscape of pulmonary artery endothelium reveals subpopulation- and disease-specific remodeling signatures
Source: Commun Biol. 2026 May 11;9:998. doi: 10.1038/s42003-026-10204-0 (PMC13385408; doi:10.1038/s42003-026-10204-0)
Supplement: Supplementary file 2 — Reporting Summary [file 42003_2026_10204_MOESM2_ESM.pdf]

nature portfolio

Corresponding author(s): Grazyna Kwapiszewska
Thomas Lins

Last updated by author(s): Apr 14, 2026

Reporting Summary

Nature Portfolio wishes to improve the reproducibility of the work that we publish. This form provides structure for consistency and transparency in reporting. For further information on Nature Portfolio policies, see our Editorial Policies and the Editorial Policy Checklist.

Statistics

For all statistical analyses, confirm that the following items are present in the figure legend, table legend, main text, or Methods section.

n/a	Confirmed	
☐	☒	The exact sample size (*n*) for each experimental group/condition, given as a discrete number and unit of measurement
☐	☒	A statement on whether measurements were taken from distinct samples or whether the same sample was measured repeatedly
☐	☒	The statistical test(s) used AND whether they are one- or two-sided<br>*Only common tests should be described solely by name; describe more complex techniques in the Methods section.*
☐	☒	A description of all covariates tested
☐	☒	A description of any assumptions or corrections, such as tests of normality and adjustment for multiple comparisons
☐	☒	A full description of the statistical parameters including central tendency (e.g. means) or other basic estimates (e.g. regression coefficient) AND variation (e.g. standard deviation) or associated estimates of uncertainty (e.g. confidence intervals)
☐	☒	For null hypothesis testing, the test statistic (e.g. *F*, *t*, *r*) with confidence intervals, effect sizes, degrees of freedom and *P* value noted<br>*Give P values as exact values whenever suitable.*
☒	☐	For Bayesian analysis, information on the choice of priors and Markov chain Monte Carlo settings
☒	☐	For hierarchical and complex designs, identification of the appropriate level for tests and full reporting of outcomes
☐	☒	Estimates of effect sizes (e.g. Cohen's *d*, Pearson's *r*), indicating how they were calculated

Our web collection on statistics for biologists contains articles on many of the points above.

Software and code

Policy information about availability of computer code

Data collection	The utilized .rds file for Seurat and the R code are saved on Zenodo and can be accessed by reviewers here:<br>https://zenodo.org/records/17793613?<br>preview=1&token=eyJhbGciOiJIUzUxMiJ9.eyJpZCI6IjQ0ZTAzYzMwLTgxMTMtNDY4Yy04N2VlLTQ3OGYzNzYxNjBiNCIsImRhdGEiOnt9LCJyYW5kb<br>20iOiIzNjRmOGZlNzc0OTA5ZWRjMWQxZWM3MjJkODkxMDAxMyJ9.1fzDbTzLioegl7_No8XXtGgn1qJWwEOCzytSSdtIsSLhsifneBNmDATNnDRHg<br>OjwHsoJNBdMT-qfbnw6R4G0BQ<br>The Code will be published with the manuscript containing DOI number after manuscript publication.
Data analysis	Raw data alignment was done via cell ranger.<br>Data analysis and visualization was performed with R based packages: Seurat(with MAST), scCustom, enrichR, Monocle3, Scillus and ggplot2.<br>Image processing was done with LASX and QuPath.

For manuscripts utilizing custom algorithms or software that are central to the research but not yet described in published literature, software must be made available to editors and reviewers. We strongly encourage code deposition in a community repository (e.g. GitHub). See the Nature Portfolio guidelines for submitting code & software for further information.

Data

Policy information about availability of data

All manuscripts must include a data availability statement. This statement should provide the following information, where applicable:

- Accession codes, unique identifiers, or web links for publicly available datasets
- A description of any restrictions on data availability
- For clinical datasets or third party data, please ensure that the statement adheres to our policy

All data pertaining human and mouse PA scRNA is available in our public access superseries GSE228644. Public available dataset GSE155468 provides aortic data and GSE131778 includes coronary artery data as provided by the original authors. Additional data containing the supplemental tables can be accessed on figshare: https://doi.org/10.6084/m9.figshare.30581477. For open access purposes, the author has applied a CC BY public copyright license to any author-accepted article version arising from this submission.

Research involving human participants, their data, or biological material

Policy information about studies with human participants or human data. See also policy information about sex, gender (identity/presentation), and sexual orientation and race, ethnicity and racism.

Reporting on sex and gender	This study was conducted with no specific sex or gender based analysis.
Reporting on race, ethnicity, or other socially relevant groupings	This work does not observe differences in race, ethnicity or social groupings.
Population characteristics	Population characteristics (demographic and clinical) are reported in the manuscript.
Recruitment	This study utlizes explant lung samples from transplant patients suffering from pulmonary arterial hypertension.
Ethics oversight	Collection of tissue and clinical data was approved by the institutional ethics committee boards: Medical University of Vienna, Vienna, Austria, ethics numbers:EK 976/2010, EK 1417/2022 and EK 35-515 ex 22/23; Medical University of Graz EK 1291/2025 and EK 35-515 ex 22_23; University of Pennsylvania, Philadelphia, Pennsylvania, USA, ethics number: PROPEL 806345

Note that full information on the approval of the study protocol must also be provided in the manuscript.

Field-specific reporting

Please select the one below that is the best fit for your research. If you are not sure, read the appropriate sections before making your selection.

☒ Life sciences ☐ Behavioural & social sciences ☐ Ecological, evolutionary & environmental sciences

For a reference copy of the document with all sections, see nature.com/documents/nr-reporting-summary-flat.pdf

Life sciences study design

All studies must disclose on these points even when the disclosure is negative.

Sample size	Sample size was chosen based on previous publications utilizing scRNA methodology and maximized based on availability.
Data exclusions	Data was excluded during QC which is based on previous publications utilizing scRNA data and was adjusted for higher stringency.
Replication	In the study, all attempts at replication were successful. Each result described in the paper is based on at least two independent biological replicates.
Randomization	The study design did not allow for meaningfull randomization and was therefore not performed.
Blinding	Investigation was not conducted under blind conditions, since patient diagnosis was pertinent for statistical comparison

Reporting for specific materials, systems and methods

We require information from authors about some types of materials, experimental systems and methods used in many studies. Here, indicate whether each material, system or method listed is relevant to your study. If you are not sure if a list item applies to your research, read the appropriate section before selecting a response.

Materials & experimental systems

n/a	Involved in the study
☐	☒ Antibodies
☒	☐ Eukaryotic cell lines
☒	☐ Palaeontology and archaeology
☒	☐ Animals and other organisms
☒	☐ Clinical data
☒	☐ Dual use research of concern
☒	☐ Plants

Methods

n/a	Involved in the study
☒	☐ ChIP-seq
☒	☐ Flow cytometry
☒	☐ MRI-based neuroimaging

Antibodies

Antibodies used

Abcam ab32457 CD31; BioRad MCA547G CD34; Invitrogen PA5-21924 THBD; R&D AF938 VE-CAD; Agilent GA527 VWF; Invitrogen PA5-53667 COL15A1; Atlas Antibodies HPA017258 ACKR1; Atlas Antibodies HPA001868 PRX; Atlas Antibodies HPA004919 HPGD; Atlas Antibodies HPA014193 PTGIS; Abcam ab32763 SULF1;

Validation

Co-staining with classical markers of the same celltype, negative control, location according to human cell atlas

Plants

Seed stocks

Report on the source of all seed stocks or other plant material used. If applicable, state the seed stock centre and catalogue number. If plant specimens were collected from the field, describe the collection location, date and sampling procedures.

Novel plant genotypes

Describe the methods by which all novel plant genotypes were produced. This includes those generated by transgenic approaches, gene editing, chemical/radiation-based mutagenesis and hybridization. For transgenic lines, describe the transformation method, the number of independent lines analyzed and the generation upon which experiments were performed. For gene-edited lines, describe the editor used, the endogenous sequence targeted for editing, the targeting guide RNA sequence (if applicable) and how the editor was applied.

Authentication

Describe any authentication procedures for each seed stock used or novel genotype generated. Describe any experiments used to assess the effect of a mutation and, where applicable, how potential secondary effects (e.g. second site T-DNA insertions, mosiacism, off-target gene editing) were examined.
