# Supplementary material for: Transcriptional landscape of pulmonary artery endothelium reveals subpopulation- and disease-specific remodeling signatures
Source: Commun Biol. 2026 May 11;9:998. doi: 10.1038/s42003-026-10204-0 (PMC13385408; doi:10.1038/s42003-026-10204-0)
Supplement: Supplementary file 3 — Transparent Peer Review file [file 42003_2026_10204_MOESM3_ESM.pdf]

nature portfolio

Peer Review File

Transcriptional landscape of pulmonary artery endothelium reveals subpopulation- and disease-specific remodeling signatures

Corresponding Author: Dr Grazyna Kwapiszewska

This file contains all reviewer reports in order by version, followed by all author rebuttals in order by version.

Version 0:

Reviewer comments:

Reviewer #1

(Remarks to the Author)
General comment: In this study, Lins et al. employ a compartment-restricted scRNA-seq approach to investigate PAEC heterogeneity in control, PH-PF, and PAH patients. This approach is well justified, as PAECs are otherwise too underrepresented in lung (EC) datasets to allow meaningful analysis of their potential heterogeneity. The data analysis is overall well conducted, with the identification of three different PAEC subpopulations, consistent across arterial beds and species, with different proportions and gene expression profiles between PH-PF, PAH and control samples. However, one of the main claims of the paper suggesting mosaicism of ECs composing the intimal layer of PAs is not convincingly supported by the presented data. Other major points described below should also be addressed prior to publication.

Major comments:
1. The claim that the PA endothelium lining the intimal layer is constituted by mosaic EC subpopulations, as inferred from scRNA-seq data, lacks strong supporting evidence:
a. Only PAECs from cluster 1 display a clear arterial EC gene signature. Other PAEC populations may therefore originate from the vasa vasorum or contaminating parenchyma, as also acknowledged in the discussion section. Given the reported limited presence of vasa vasorum in mouse PAs, the lower proportion of cluster 2 PAECs in this sample type (Fig. 4F) would be consistent with cluster 2 PAECs being derived from the vasa vasorum rather than from the intimal layer of PAs. Moreover, the presence of an epithelial cell cluster in the dissected PAs (EPCAM+ cells in Supp. Fig. 1) also suggests a small contamination from the surrounding lung tissue.
b. PAECs from cluster 0 expressed PLVAP according to Fig. 1F and Fig. 3B. PLVAP is a marker of fenestrations, a feature that is usually not observed in arterial endothelium, suggesting ECs from another location than the PA intima.
c. The pseudotime trajectory shown in Fig. 3 follows a typical arterial–capillary–venous continuum and therefore does not specifically support the proposed PAEC mosaicism claim.
d. Markers used for validation are expressed across PAECs from different clusters, notably ACKR1, PTGIS, SULF1, and PRX. Multiplex immunostaining of markers from all three clusters on the same samples would be necessary to exclude co-expression and to validate cluster specificity of the stained PAECs.
e. In Fig. 2G–I, precision-cut lung slice staining of the different markers appears largely continuous and overlaps with VWF, rather than displaying a mosaic pattern. The authors describe the staining as “homogenously along PA walls”. This does not clearly support the proposed cellular heterogeneity.
f. The authors used VWF as a positive control for PAECs. Can they provide evidence for the absence of VWF expression in ECs from other blood vessels in lungs?
g. Immunostaining validation was performed on tissue sections containing very small arteries (down to ~150 µm in diameter; Fig. 2E), raising the question of whether these vessels correspond to those used for dissection. Crnkovic S et al. study referred to arteries <5 mm in diameter but no minimal diameter was specified. This reviewer would therefore recommend to perform immunostaining on the dissected PAs instead of tissue sections.
2. A major limitation of the study, which is not explicitly addressed, is the potential batch effect introduced by the addition of new samples (two controls and all three PH-PF samples) to a previously generated dataset. The authors should provide explicit evidence demonstrating that batch effects were assessed and corrected if necessary.
3. While the ligand–receptor interaction analysis is of interest, it lacks validation. In addition, ligands dysregulated in both PAH and PH-PF contexts may be particularly relevant and should be included in Fig. 9, at least for the top-ranked

candidates.
4. PAECs in PH have been characterized as hyperproliferative and apoptosis-resistant, yet the data presented here do not clearly support such phenotype. This should be further discussed. In addition, given previous reports of EndoMT in PH, the authors should comment on whether their dataset provides evidence for EndoMT.
5. A table summarizing the clinical characteristics of PH patients and controls used in this study is missing and should be included. At a minimum, this table should report age and sex for all subjects, and for PH patients additionally include mPAP, PVR, cardiac index, NYHA functional class, and therapies received. Moreover, it is unclear whether the samples used for immunostaining were derived from the same patients as those from whom pulmonary arteries were dissected, or from different individuals. This should be clarified.

Minor comments:
1. The rationale for labeling cluster 2 PAECs as “stress-response” is unclear. Classical cellular stress responses are typically associated with the induction of immediate early genes (e.g., FOS, JUN, EGR1), whereas alternative stress programs (e.g., more chronic) would require distinct transcriptional evidence, which is not clearly presented.
2. The number of PAECs obtained in silico, per patient should be reported.
3. Potential differences in ischemia time between control and diseased samples could affect gene expression data. The authors should clarify whether ischemia times were comparable and, if not, discuss this as a limitation.
4. Labels are missing in Fig. 7E.

Reviewer #2

(Remarks to the Author)
In the manuscript, Transcriptional landscape of pulmonary artery endothelium reveals 1 subpopulation- and 2 disease-specific remodeling signatures, the authors have concluded via single cell RNA sequencing analysis of endothelial cells from healthy donors, patients with pulmonary arterial hypertension and patients with pulmonary hypertension associated with pulmonary fibrosis, that there are three distinct subpopulations of endothelial cells which they term immunomodulatory, vascular tone modulatory, and stress response modulatory. They argue that these distinct endothelial subsets are heterogenous, and vary in abundance in different disease states and in accordance to the distinct need of the vascular bed from which they are found. They show that existence of these endothelial cell subsets is conserved across aortic cells, coronary artery cells, and murine pulmonary arteries, however abundance varies. They suggest that these findings may provide a biological basis for the lack of efficacy of certain types of pulmonary hypertension therapies in certain subsets of PH and provide a framework for future application of transcriptomics to therapeutic approaches for PH.

This study is novel, adds important transcriptomic information to the field of pulmonary vascular disease research, and provides extensive characterization of the described subsets of PAECs. Below are some comments that I feel would clarify and strengthen the manuscript prior to publication.

Major Comments

1. It seems that the use of grouping control donor tissue with diseased samples, both PAH and PF-PH, create challenges when interpreting the data, particularly given that in subsequent experiments these are shown to differ widely between groups. It is not clear why this approach was taken and what steps the authors have utilized or considered during analysis of this data. How could this have affected pseudo-time analysis if one subset of the group is driving these changes. There would also be a concern when comparisons are made between PA, Ao, and CA samples given that the PA samples represent 3 distinct groups. Similarly, when donor PAEC are compared to PH, as a combination of both PAH and PF-PH. The authors should address the decision to group samples for each particular experiment.

2. Are any other patient comorbidities known that could be relevant to the findings comparing PF-PH to PAH? Presence of hypertension, autoimmune conditions, infections, diabetes, smoking history, need for supplemental oxygen? In addition, RHC data, age, sex, BMI, etc.? If known, would recommend inclusion of this data. Particularly in the context of the last several paragraphs of the Discussion section, if there is significant variability in the severity of the PH between different subgroups, is it possible that some of the observed differences in transcriptomic profiles is related to progression of disease rather than subtype of disease?

3. The pie charts depict subcluster EC population proportions in AO, CA and PA. Proportions are shown as medians and interquartile range. Especially in PA cells, these are large interquartile ranges. Can the authors comment on this and what statistics were used for these analyses?

Immuno 30.2% (16.45% - 43.95%)
Stress Response 28.3% (9.75% - 46.85%)
Vascular tone 42.1% (21.9% - 62.3%)

Similarly, for Figure 7A, these are again high interquartile ranges. What statistics were used for these analyses? Can the authors present the individual percentages per patient for a better sense of the heterogeneity? While human tissue is a precious resource and this is a large n for a study such as this, a comment on the heterogeneity would be helpful, particularly in the context of applying different treatment strategies to different subtypes of PH based on these subsets which appear to be heterogenous across different patients.

Immuno 55.4% (31.9% - 78.9%)

Stress Response 6.58% (4.97% - 8.19%)
Vascular tone 39.7% (12.1% - 67.3%)

4. In Figure 3 (right), could more detail and clarity be provided to the UMAP on the right? The terminology “nodes (crossings), leaves (endpoints) and the calculated pseudotime (right)” is unclear and it is difficult to appreciate the numbers/colors of the circled nodes on the plot.

Minor Comments

1. In lines 63-67, the authors state “Despite the accumulating knowledge on transcriptional and functional changes of the endothelium in diseases involving the pulmonary vasculature 23,28–32 65, and growing interest in endothelial heterogeneity, PAECs have not been examined at high resolution, particularly in clinically distinct subtypes such as PAH and PH associated with pulmonary fibrosis (PH-PF).” I would consider the additions of citations of the following manuscripts of single cell RNA sequencing and ensure the prior statement remains correct:

• Liu B, Yi D, Li S, Ramirez K, Xia X, Cao Y, Zhao H, Tripathi A, Qiu S, Kala M, Rafikov R, Gu H, de Jesus Perez V, Lemay SE, Glembotski CC, Knox KS, Bonnet S, Kalinichenko VV, Zhao YY, Fallon MB, Boucherat O, Dai Z. Single-cell and Spatial Transcriptomics Identified Fatty Acid-binding Proteins Controlling Endothelial Glycolytic and Arterial Programming in Pulmonary Hypertension. bioRxiv [Preprint]. 2024 Apr 15:2024.02.11.579846. doi: 10.1101/2024.02.11.579846. Update in: Arterioscler Thromb Vasc Biol. 2025 Jul;45(7):1145-1165. doi: 10.1161/ATVBAHA.124.321173. PMID: 38370670; PMCID: PMC10871348.

• Tang B, Vadgama A, Redmann B, Hong J. Charting the cellular landscape of pulmonary arterial hypertension through single-cell omics. Respir Res. 2024 May 3;25(1):192. doi: 10.1186/s12931-024-02823-0. PMID: 38702687; PMCID: PMC11067161.

• Monteiro, João P et al. “MIR503HG Loss Promotes Endothelial-to-Mesenchymal Transition in Vascular Disease.” Circulation research vol. 128,8 (2021): 1173-1190. doi:10.1161/CIRCRESAHA.120.318124

2. Line 88-89 The cell markers and cell lineage description should align in this sentence for simplicity and understanding of the reader i.e. PTPRC, ACTA2, EPCAM1, PDGRA and LYVE1 (Supplemental Table 1, 2 – not finding any of the supplemental tables) for immune, smooth muscle, epithelial, fibroblast, and lymphatic cells respectively.

3. In Supplemental Figure 1C, how do you account for the ACTA2 expression in the endothelial population? Does this signify endothelial to mesenchymal transition or an expected expression of ACTA2 in endothelial cells?

4. Methodology:
a. While it is stated in the cited paper Crnkovic S., Valzano F. et.al. 2022, could the authors confirm in the text that small and medium pulmonary arteries were those that were <5mm?
b. For the Supplemental Figure 4 and line 138 how were pulmonary veins collected/processed? I could not find this methodology in the cited text.
c. How were precision-cut lung slices carried out? If the same methodology from this citation, please indicate this.
d. Can the authors add information about how the pseudotime analysis was carried out in their methodology? See major comments #4.

5. Additional Minor points:
a. In line 127, citation 25,34 not superscript.
b. In Figure 7E, red stain does not have a label.
c. In Figure 8A-C (right), what is the unit for the y axis?
d. In Supplemental Figure 2, it would be helpful to increase the height of the image in order to better see the genes on the Y axis.
e. Supplemental Figure 7A “stress response modulting”
f. Line 279-281, include reference to the Figure that demonstrates this point for clarity

Reviewer #3

(Remarks to the Author)
The MS by Lins et al. define the heterogeneity of pulmonary artery endothelial cells under basal condition and pulmonary vascular disease conditions. Employing isolated medium to small pulmonary arteries for scRNAseq, the authors revealed 3 EC subpopulations including immuno- vascular tone- and stress response modulatory. Both PAH and PH-PF altered PAEC subset compositions with increased immunomodulatory and decreased stress response modulatory ECs. PAH-PAECs exhibit lipid metabolic dysfunction and enhanced vasoregulatory signaling while PH-PF PAECs were generally defined by dysregulated angiogenesis and antigen presentation. The study integrates scRNAseq, spatial mapping and ligand-receptor analysis providing novel information about pulmonary arterial endothelial heterogeneity in the pathogenesis of PAH and PF-induced PH. Impressively, the study also compared the PA EC heterogeneity with human aortic arterial EC and carotid arterial ECs. This is a timely study paving the way toward precision medicine for therapy of pulmonary vascular diseases.

One major comment is the small number of patient samples. At least n=5 patients each disease (i.e. 5 PAH and 5 PF-PH) should be included for the study to enhance reproducibility. Other minor comments include 1) some references need to be

updated, e.g. ref26, all references should be rechecked. 2) Fig 5C should use text instead of color to label Donor and PH samples for better clarity.

Version 1:

Reviewer comments:

Reviewer #1

(Remarks to the Author)
This reviewer thanks the authors for their detailed point-by-point reply and additional experiments performed. Most of the concerns raised by this reviewer have been addressed. However, there are still remaining issues especially regarding the contribution of vasa vasorum ECs and the batch-correction strategy.

Major comments:
1. A non-negligible fraction of PAECs in the scRNA-seq dataset may derive from vasa vasorum residing within the walls of 2–5 mm pulmonary arteries. Although briefly mentioned in the discussion section, this possibility should be emphasized in the results, particularly since vasa vasorum ECs express both VWF and ACKR1 (Burns et al., Microvascular Research 2023; Thiriot et al., BMC Biology 2017), the latter being a marker of the immunomodulatory EC cluster identified by the authors. Additional images of isolated PAs stained for the study markers and VWF, as already performed, would help confirm or exclude a contribution from vasa vasorum ECs.
2. The authors appropriately applied CCA and Harmony for the batch correction during integration and for clustering. Could batch effects also be addressed for the differential gene expression analyses? CCA and Harmony are typically applied to a subset of genes for sample integration, as shown here, but are not appropriate for differential expression tests.

Minor comments:
1. In the updated Fig. 7B–C, PAH and PH-PF samples lacking “vascular plasticity modulatory” ECs (value = 0) should be included in the analysis.
2. How was arteriole identity confirmed for vessels shown in the new Figures 2D, 7D, and 7E? The PH vessels in Fig. 7 do not display clear remodeling features.
3. Intimal hyperplasia is most prominent in small-diameter arterioles in PH. Given the larger PA diameter analyzed here (2-5mm diameter), this may explain the absence of apoptosis-resistant, hyperproliferative, or EndoMT signatures in the PAEC scRNA-seq data. That point could be further discussed in the discussion section of the manuscript.

Reviewer #2

(Remarks to the Author)
In the manuscript, Transcriptional landscape of pulmonary artery endothelium reveals 1 subpopulation- and 2 disease-specific remodeling signatures, the authors have concluded via single cell RNA sequencing analysis of endothelial cells from healthy donors, patients with pulmonary arterial hypertension and patients with pulmonary hypertension associated with pulmonary fibrosis, that there are three distinct subpopulations of endothelial cells which they term immunomodulatory, vascular tone modulatory, and stress response modulatory which vary across disease states and may be related to disease response to certain therapies.

This study is novel, adds important transcriptomic information to the field of pulmonary vascular disease research, and provides extensive characterization of the described subsets of PAECs. The authors have made extensive revisions and provided a written response to prior comments. They have satisfied my concerns and I have no further comments.

Reviewer #3

(Remarks to the Author)
The authors have addressed all my comments.

Version 2:

Reviewer comments:

Reviewer #1

(Remarks to the Author)
This reviewer thanks the authors for their thorough revision and congratulates them on this excellent work. All comments have been adequately addressed.

Open Access This Peer Review File is licensed under a Creative Commons Attribution 4.0 International License, which permits use, sharing, adaptation, distribution and reproduction in any medium or format, as long as you give appropriate credit to the original author(s) and the source, provide a link to the Creative Commons license, and indicate if changes were made.
In cases where reviewers are anonymous, credit should be given to 'Anonymous Referee' and the source.
The images or other third party material in this Peer Review File are included in the article's Creative Commons license, unless indicated otherwise in a credit line to the material. If material is not included in the article's Creative Commons license and your intended use is not permitted by statutory regulation or exceeds the permitted use, you will need to obtain permission directly from the copyright holder.
To view a copy of this license, visit https://creativecommons.org/licenses/by/4.0/

Reviewer1:

Major Comments:

1) The claim that the PA endothelium lining the intimal layer is constituted by mosaic EC subpopulations, as inferred from scRNA-seq data, lacks strong supporting evidence:

a) Only PAECs from cluster 1 display a clear arterial EC gene signature. Other PAEC populations may therefore originate from the vasa vasorum or contaminating parenchyma, as also acknowledged in the discussion section. Given the reported limited presence of vasa vasorum in mouse PAs, the lower proportion of cluster 2 PAECs in this sample type (Fig. 4F) would be consistent with cluster 2 PAECs being derived from the vasa vasorum rather than from the intimal layer of PAs. Moreover, the presence of an epithelial cell cluster in the dissected PAs (EPCAM+ cells in Supp. Fig. 1) also suggests a small contamination from the surrounding lung tissue.

We thank the reviewer for raising this important concern. We agree that, given the nature of sample preparation, contributions from surrounding parenchyma or the vasa vasorum cannot be fully excluded. To address this, we performed additional analyses to assess whether such contamination could account for the observed endothelial heterogeneity.

Because epithelial cells should not be present in dissected PAs under ideal preparation conditions, we used the epithelial cell population as a quantitative indicator of parenchymal contamination. For each sample, the fraction of epithelial cells was calculated and treated as a conservative upper-bound estimate of potential parenchymal endothelial contamination. We then tested whether endothelial subcluster composition covaried with epithelial burden across samples. No endothelial subpopulation was associated with epithelial contamination, and samples with higher epithelial fractions did not show systematic shifts in endothelial subcluster abundance. This data is now included in the revised manuscript and in the new Supplemental Fig. 4A–C.

In parallel, IF staining of tissue and isolated PAs confirmed the presence of marker-positive cells corresponding to all identified endothelial subpopulations within the PA intima (Figure 2 DE; Figure7 D-G; Supplemental Figure 6 A-C; Supplemental Figure 7 AC,DE; Supplemental Figure 8 AC; Supplemental Figure 11 A-F), arguing against a specific association with parenchyma or the vasa vasorum. Consistent with this interpretation, similar endothelial expression profiles as identified in the PA were observed in non-pulmonary arterial beds (aorta and coronary artery), supporting the conclusion that these populations represent authentic arterial endothelial states rather than tissue contamination.

Taken together, these orthogonal analyses indicate that the identified endothelial subclusters reflect distinct endothelial populations of the PA intima and are not driven by parenchymal contamination or vasa vasorum–derived cells.

b) PAECs from cluster 0 expressed PLVAP according to Fig. 1F and Fig. 3B. PLVAP is a marker of fenestrations, a feature that is usually not observed in arterial endothelium, suggesting ECs from another location than the PA intima.

We thank the reviewer for mentioning this and agree, that PLVAP is normally regarded as marker of fenestration. However, expression of PLVAP in our dataset aligns with expression data in other publicly available pulmonary datasets[1] indicating PLVAP expression in arterial, venous and peribronchial/systemic ECs of the lung (see figure below). Furthermore, PVLAP has not only been reported as central regulator of vascular permeability, but also to be important during inflammatory challenges potentially exhibiting functions beyond endothelial fenestration[2]. We have included these information and related references into the discussion of the revised manuscript.

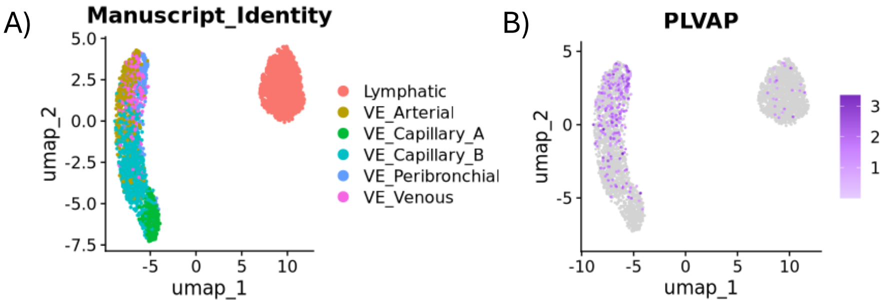

(A) UMAP representation of the endothelial cell subset derived from GSE136831, annotated according to the original authors' cell-type nomenclature.

(B) UMAP showing PLVAP expression levels across endothelial cells.

c) The pseudotime trajectory shown in Fig. 3 follows a typical arterial–capillary–venous continuum and therefore does not specifically support the proposed PAEC mosaicism claim.

We agree that the trajectory does not specifically support the proposed PAEC mosaicism claim and can both be interpreted as contextualizing PAEC functional states along a continuous gradient and the classic arterial–capillary–venous continuum. Therefore, to strengthen our interpretation of PA endothelial mosaicism, we have included additional multiplex immunofluorescence in the revised manuscript as suggested below. The inclusion of this data confirms the presence of all three endothelial subpopulation markers within the intima of pulmonary arteries (Figure 2DE, Figure 7D–G) and provides additional support for the interpretation of PAEC heterogeneity as distinct functional states.

d) Markers used for validation are expressed across PAECs from different clusters, notably ACKR1, PTGIS, SULF1, and PRX. Multiplex immunostaining of markers from all three clusters on the same samples would be necessary to exclude co-expression and to validate cluster specificity of the stained PAECs.

We fully agree with the value of such multiplex stainings and have accordingly now provided simultaneous immunofluorescence staining of ACKR1, PTGIS, and PRX on lung tissue sections and isolated PAs (FFPE). These stainings demonstrate marker expression of all three subpopulations with largely exclusive signal and minimal overlap in endothelial cells. The corresponding images are shown in Figure 2DE and Figure 7D–G of the revised manuscript.

e) In Fig. 2G–I, precision-cut lung slice staining of the different markers appears largely continuous and overlaps with VWF, rather than displaying a mosaic pattern. The authors describe the staining as "homogenously along PA walls". This does not clearly support the proposed cellular heterogeneity.

We apologize for the confusing wording in the original manuscript. We have rephrased our statement in the revised manuscript for clarity, and added multiplex IF staining to confirm cellular heterogeneity and mosaicism (as described in detail in the previous response and in the revised manuscript, and Figure 2DE and Figure 7D-G. Original Figure 2G–I (now Figure 2F-H) presents contextual evidence of marker expression within the three-dimensional pulmonary arterial vasculature, while mosaic organization is supported by the newly added multiplex and trajectory analyses.

f) The authors used VWF as a positive control for PAECs. Can they provide evidence for the absence of VWF expression in ECs from other blood vessels in lungs?

We thank the reviewer for raising this point and apologize for any lack of clarity in our initial description. We agree that VWF is not exclusively expressed in PAECs but is also present in ECs of the pulmonary microvasculature and venous compartment.

Importantly, VWF expression distinguishes macrovascular ECs from capillary ECs in the lung, where VWF expression is either low or absent[3,4] Therefore, we used VWF as an internal control to confirm that all three identified endothelial subpopulations are present within the PA (macrovascular) compartment, rather than to claim specificity for PAECs over all other lung ECs. This was particularly relevant given that one of the identified PAEC subpopulations exhibits transcriptional features that partially overlap with capillary-associated gene signatures. This approach has been clarified and described in more detail in the Material and Methods, and Results section of the revised manuscript.

g) Immunostaining validation was performed on tissue sections containing very small arteries (down to ~150 µm in diameter; Fig. 2E), raising the question of whether these vessels correspond to those used for dissection. Crnkovic S et al. study referred to arteries <5 mm in diameter but no minimal diameter was specified. This reviewer would therefore recommend to perform immunostaining on the dissected PAs instead of tissue sections.

We thank the reviewer for this important comment. To ensure comparability between our scRNA-seq dataset and protein staining, we have included additional immunofluorescence images of isolated, formalin-fixed and paraffin-embedded PAs.

Please see the new Figures 2DE and 7D-G, and the original single population marker pictures have now been moved to the supplement (Supplemental Figure 6, Supplemental Figure 11). These arteries were isolated using the same procedure as those used for scRNA-seq library preparation and were in a size range of 2–5 mm in diameter. Vessel identification was performed under a stereomicroscope based on anatomical proximity to airways and excision of the PA as a single intact segment and following removal of surrounding lung tissue. A detailed description of the isolation process is provided in the revised Materials and Methods section.

2). A major limitation of the study, which is not explicitly addressed, is the potential batch effect introduced by the addition of new samples (two controls and all three PH-PF samples) to a previously generated dataset. The authors should provide explicit evidence demonstrating that batch effects were assessed and corrected if necessary

We thank the reviewer for highlighting this important point. We have now improved the description of our approach in the methods section. All samples were processed using the same experimental protocol and sequencing chemistry (10x genomics) within a defined time frame (earliest to last lung was 6 months), limiting technical variability across datasets. For downstream analysis, raw data from all samples were processed using a uniform pipeline with identical filtering and normalization parameters. To account for potential batch effects introduced by integrating newly generated samples with the existing dataset, we applied established batch correction methods, including canonical correlation analysis (CCA) and Harmony. The performance of these integration approaches was quantitatively evaluated using the Local Inverse Simpson Index (LISI), demonstrating effective mixing of samples while preserving biological structure (Supplemental Table 33).

Consistent with these quantitative results, comparison of unintegrated and CCA-integrated UMAP embeddings shows that cell types cluster by identity rather than by sample origin or acquisition time, with no evidence of dominant batch-driven separation (as shown in the figure below). Importantly, integration preserved condition-specific and cell type–specific patterns, indicating that batch correction mitigated technical variation without overcorrecting biological signals.

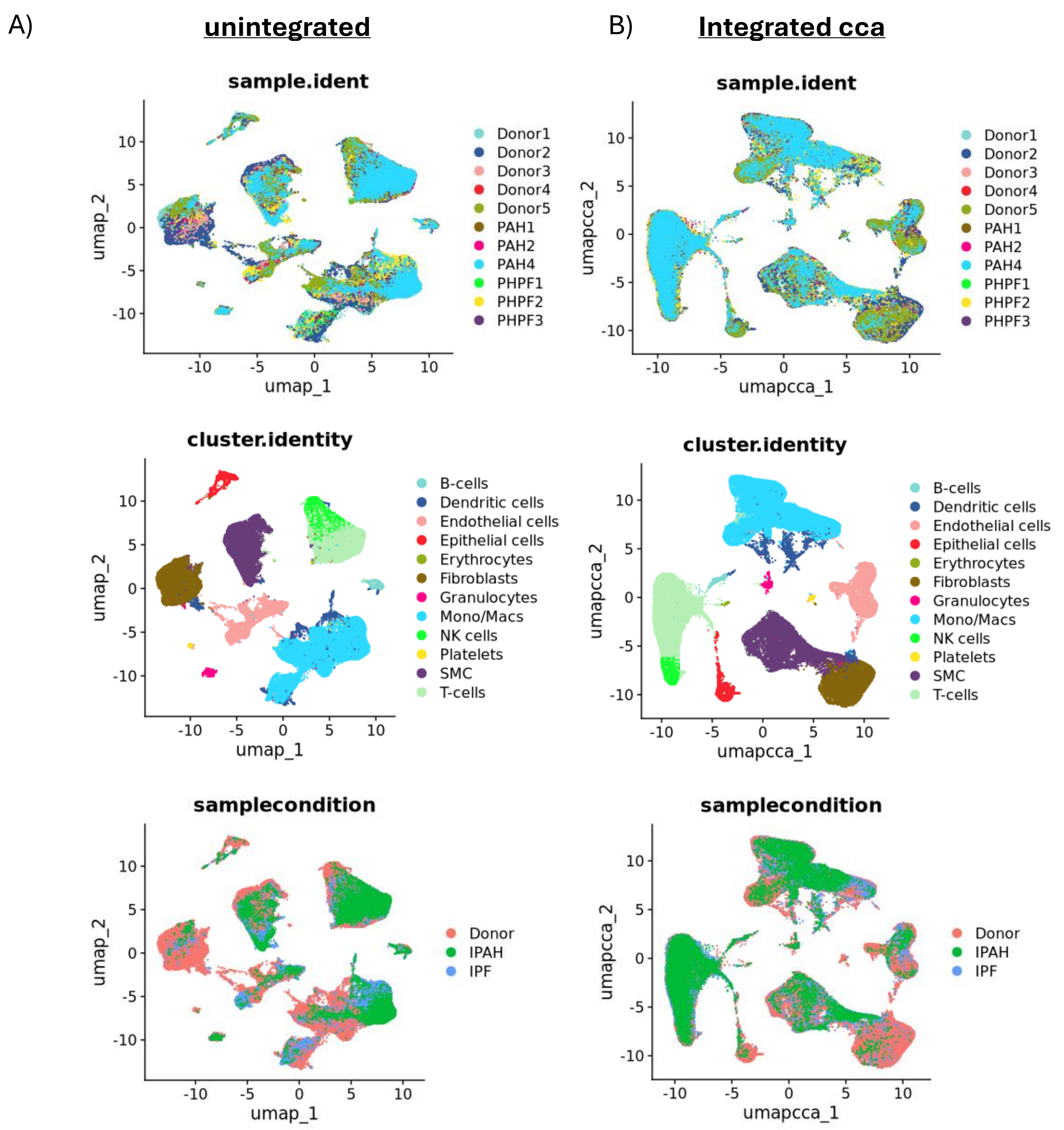

(A) UMAP representation of the full dataset generated from the PCA embedding of the unintegrated data. Panels display sample identity (top), annotated cell types (middle), and disease condition (bottom).

(B) UMAP representation of the full dataset generated from the PCA embedding of the CCA integrated assay. Panels display sample identity (top), annotated cell types (middle), and disease condition (bottom).

3)While the ligand–receptor interaction analysis is of interest, it lacks validation. In addition, ligands dysregulated in both PAH and PH-PF contexts may be particularly relevant and should be included in Fig. 9, at least for the top-ranked candidates.

We thank the reviewer for this important feedback. Ligand–receptor interactions were inferred using established computational frameworks that integrate curated interaction databases and gene expression patterns. While these approaches have been widely used and benchmarked in prior studies, they provide predictive rather than experimental validation and should therefore be interpreted as hypothesis-supporting or -generating. In addition, we performed a detailed evaluation of ligands with heightened interaction probability in the revised analysis.

In response to the reviewer's suggestion, we included ligands and receptors that are shared between PAH and PH-PF and exhibit heightened interaction probability in the revised figures (Figure 9 A,B). To further assess the functional relevance of the inferred communication networks, we integrated CellChat and NicheNet analyses (Figure below). Disease-specific ligand–receptor interactions identified by CellChat (A-C) were not enriched among functionally active ligands predicted by NicheNet (D), and PF- and PAH-specific ligands showed activity scores indistinguishable from the remaining candidate ligands evaluated by NicheNet. These findings highlight an important limitation of ligand–receptor inference approaches in predicting downstream transcriptional effects. Despite distinct communication architectures, endothelial transcriptional responses converged across diseases, suggesting that different upstream signalling networks may lead to a shared vascular pathological state.

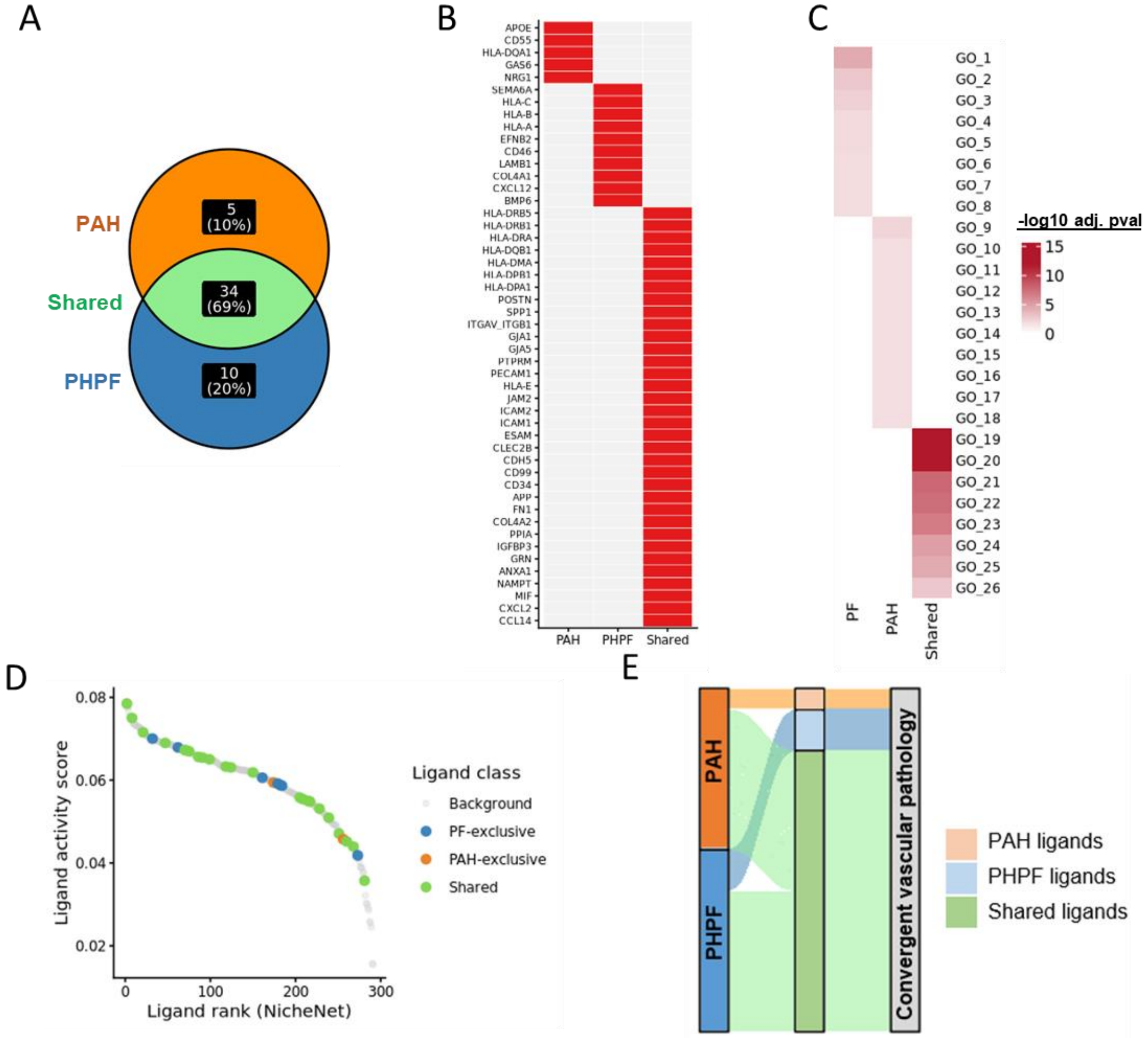

(A–C) CellChat analysis identifying ligands with increased interaction probability toward endothelial cells in pulmonary arterial hypertension (PAH) and pulmonary hypertension associated with pulmonary fibrosis (PH-PF). (A) Venn diagram showing PAH-specific, PH-PF-specific, and shared ligands. (B) Visualization of ligand classes highlighting disease-exclusive and shared signaling inputs. (C) Gene ontology enrichment of CellChat-identified ligands reveals distinct upstream signaling pathways in PAH (signaling-driven, receptor- and lipid-centric vascular remodelling) and PH-PF (immune-driven, cytokine-rich, matrix-building pathology), alongside overlapping functional themes (endothelial activation and immune-matrix communication).

(D–E) NicheNet analysis assessing the functional relevance of CellChat-derived ligands based on their predicted ability to regulate disease-associated endothelial gene expression. (D) Ligand activity ranking demonstrates that disease-specific and shared CellChat ligands are distributed across the NicheNet activity spectrum relative to background ligands. (E) Sankey diagram illustrating convergence of distinct PAH- and PH-PF-associated ligands onto overlapping downstream transcriptional programs, indicating functional convergence despite possible divergent upstream communication architectures.

4) PAECs in PH have been characterized as hyperproliferative and apoptosis-resistant, yet the data presented here do not clearly support such phenotype. This should be further discussed. In addition, given previous reports of EndoMT in PH, the authors should comment on whether their dataset provides evidence for EndoMT.

Based on the reviewers valuable comment, we expanded our analysis and investigated EndoMT based on a set of well-approved EndoMT marker genes[5–7] (Supplemental Table 34) in our scRNAseq dataset. Our data does not point towards any separate population specifically undergoing EndoMT which we clarified in the revised manuscript (Supplemental Figure 13). We have additionally expanded on our discussion dealing with the lack of hyperproliferation, apoptosis -resistance and EndoMT in our data set. Notably, the analyzed samples were derived from end-stage disease, which may explain the absence of pronounced hyperproliferative and apoptosis resistant phenotypes at this stage of disease progression[8].

5) A table summarizing the clinical characteristics of PH patients and controls used in this study is missing and should be included. At a minimum, this table should report age and sex for all subjects, and for PH patients additionally include mPAP, PVR, cardiac index, NYHA functional class, and therapies received. Moreover, it is unclear whether the samples used for immunostaining were derived from the same patients as those from whom pulmonary arteries were dissected, or from different individuals. This should be clarified.

A table showing demographic and all available clinical data has been added to the revised manuscript (Table 1), as suggested by the reviewer. Importantly, no significant differences between the PAH and PH-PF cohort were detected, except for the mean pulmonary arterial pressure (mPAP). mPAP of PAH patients was in the range of 68 to 77 mmHg, while PH-PF patients had a mPAP ranging from 27 to 38 mmH, reflecting the fact that vascular remodelling in PAH is the primary cause of disease and not a consequence of another underlying lung condition, such as pulmonary fibrosis. This data has been included and discussed in the revised manuscript (Table 1).

Tissue sections used for immunofluorescence stainings were not derived from the same patients as were used for the scRNA sequencing, which further extends our cohort size and thus substantiates our findings. This approach has been clarified in the manuscript.

Minor comments:

1) *The rationale for labeling cluster 2 PAECs as “stress-response” is unclear. Classical cellular stress responses are typically associated with the induction of immediate early genes (e.g., FOS, JUN, EGR1), whereas alternative stress programs (e.g., more chronic) would require distinct transcriptional evidence, which is not clearly presented.*
We agree that stress response is not an ideal name. After careful revision of the data, we have therefore renamed the cluster to “vascular plasticity modulating”. Vascular plasticity is the ability of blood vessels to remodel their structure and function in response

to environmental, developmental, or injury-related stimuli, such as angiogenesis. This description keeps in line with the message we wanted to convey while avoiding ambiguity. The new naming was chosen based on the gene expression signature as seen in Figures 1H and K. This subset is characterized by coordinated regulation of cell-cycle priming (RGCC, CCND1), cytoskeletal remodelling (Rho/Rac regulators), signal integration (EGFR, FZD4), and junctional modulation (JAM3, CD36) consistent with a plasticity-associated, modulatory endothelial state.

2) The number of PAECs obtained in silico, per patient should be reported.

We have added the PAEC cell counts to the new Supplementary Figure 2. It shows UMAP plots of all samples separately including their total cell counts.

3) Potential differences in ischemia time between control and diseased samples could affect gene expression data. The authors should clarify whether ischemia times were comparable and, if not, discuss this as a limitation.

We agree that this information should be included in our manuscript. The revised manuscript now includes a statement explaining that lung tissue samples from control and PH donors were processed using comparable procurement and handling protocols, and no systematic differences in ischemia time were reported.

4) Labels are missing in Fig. 7E.

Thank you for bringing this to our attention, the labels have now been added in the revised figure.

Reviewer2:

Major Comments:

1) It seems that the use of grouping control donor tissue with diseased samples, both PAH and PF-PH, create challenges when interpreting the data, particularly given that in subsequent experiments these are shown to differ widely between groups. It is not clear why this approach was taken and what steps the authors have utilized or considered during analysis of this data. How could this have affected pseudo-time analysis if one subset of the group is driving these changes. There would also be a concern when comparisons are made between PA, Ao, and CA samples given that the PA samples represent 3 distinct groups. Similarly, when donor PAEC are compared to PH, as a combination of both PAH and PF-PH. The authors should address the decision to group samples for each particular experiment.

We thank the reviewer for raising this important point and agree that sample grouping requires careful justification, particularly given the observed differences between disease states in later analyses. In our analyses, we did not observe disease-specific segregation of ECs into distinct clusters. Instead, cells from control, PAH, and PF-PH samples consistently occupied overlapping transcriptional states. On this basis, overlapping clusters were treated as representing the same endothelial subpopulations, allowing us to first characterize their shared functional features independent of disease origin and thereby increase statistical power (Fig. 1F–K).

To ensure that this integration did not bias clustering or downstream analyses, the new Supplemental Figure 2 demonstrates that no individual sample disproportionately influenced cluster structure. In addition, we included local inverse Simpson index calculations (Supplemental Table 33), confirming balanced sample representation and integration within optimal parameters. Under these conditions, the pseudo time trajectory and baseline gene-of-interest analyses were stable within clusters and not driven by a single disease or sample subset.

The combined analysis of pulmonary artery (PA), aorta (AO), and coronary artery (CA) samples was designed to provide an unbiased assessment of shared and distinct endothelial transcriptional programs across arterial vascular beds. To this end, full datasets from each compartment were first concatenated and jointly integrated prior to any cell type–specific subsetting. ECs were only identified and analyzed after this integration step, ensuring that clustering and alignment were not influenced by pre-defined tissue labels or vascular origin.

This strategy allowed transcriptional similarities and differences between arterial beds to emerge in a data-driven manner. Had ECs from AO or CA exhibited distinct expression programs unrelated to the PA-derived clusters, they would have segregated independently during integration. Instead, ECs from all three vascular compartments

mapped onto the same transcriptional states, supporting the interpretation that these clusters represent conserved arterial endothelial archetypes.

Importantly, this integrated cross compartment analysis was performed independently of the prior PA only analysis and remained unbiased through Fig. 4D, at which point module scores were applied solely to aid cluster recognition and functional annotation rather than to influence clustering or integration.

Finally, to further exclude potential bias introduced by joint integration, we repeated the analysis independently for the PA and CA datasets, including dataset integration, EC subsetting, reclustering, and module score–based cluster identification. This separate analysis yielded comparable endothelial subpopulation structures and marker profiles, indicating that the identified clusters are not artifacts of combined dataset processing but represent reproducible endothelial transcriptional states across vascular beds (see figure below).

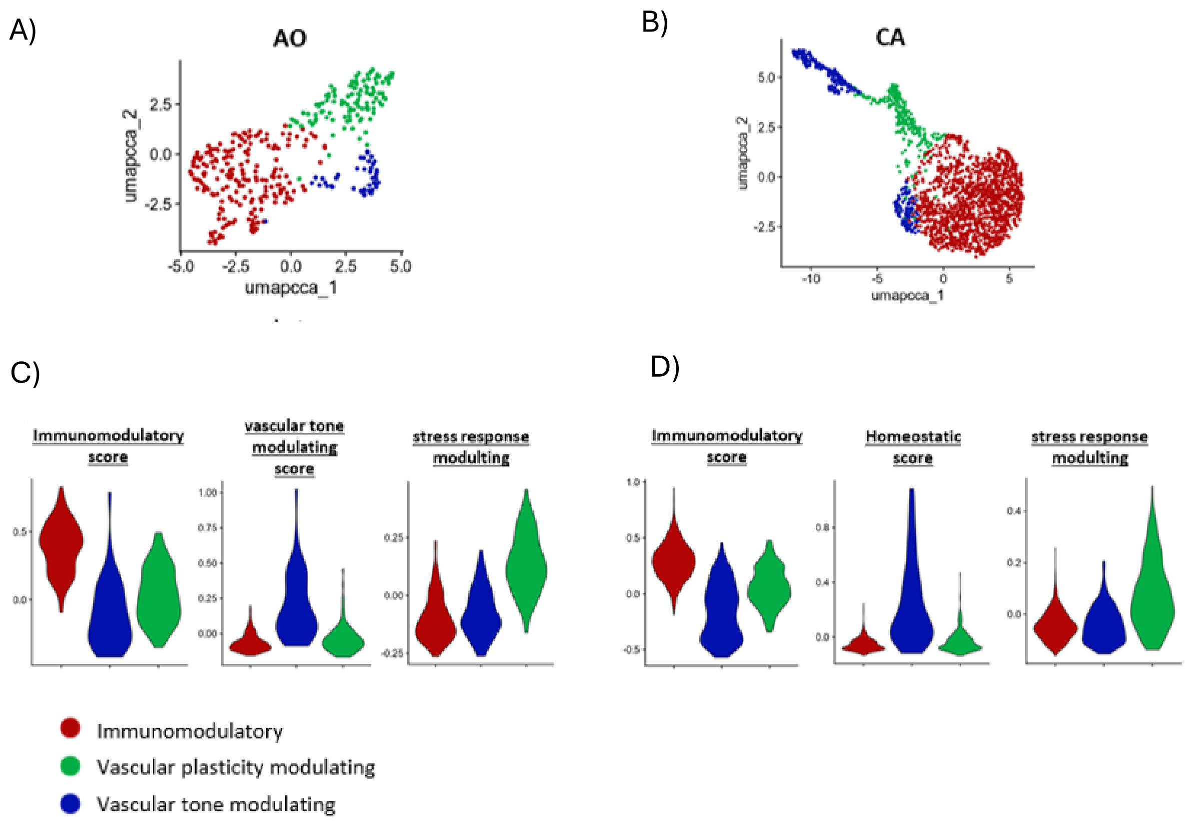

(A) UMAP of the endothelial cell subset from GSE155468 following independent clustering. Color coding was assigned based on module score results shown in panel (C).

(B) UMAP of the endothelial cell subset from GSE131778 following independent clustering. Color coding was assigned based on module score results shown in panel (D).

(C) Seurat module score analysis of previously defined endothelial subpopulation gene signatures (immunomodulatory, vascular plasticity–modulating, and stress response–modulating) in GSE155468, visualized as violin plots.

(D) Seurat module score analysis of previously defined endothelial subpopulation gene signatures (immunomodulatory, homeostatic, and stress response–modulating) in GSE131778, visualized as violin plots.

2) Are any other patient comorbidities known that could be relevant to the findings comparing PF-PH to PAH? Presence of hypertension, autoimmune conditions, infections, diabetes, smoking history, need for supplemental oxygen? In addition, RHC data, age, sex, BMI, etc.? If known, would recommend inclusion of this data. Particularly in the context of the last several paragraphs of the Discussion section, if there is significant variability in the severity of the PH between different subgroups, is it possible that some of the observed differences in transcriptomic profiles is related to progression of disease rather than subtype of disease?

This concern aligns with that raised by Reviewer 1. We agree that inclusion of patient data strengthens the manuscript and have therefore added a table summarizing the demographic and available clinical characteristics of the scRNA-seq samples used (Table 1). Overall, the samples were homogeneous, with no significant differences observed between the PAH and PH-PF group, with the exception of mean pulmonary arterial pressure (mPAP). As expected, mPAP values were markedly higher in PAH patients (68–77 mmHg) compared with PH-PF patients (27–38 mmHg), reflecting the fact that pulmonary vascular remodeling is the primary driver of disease in PAH, rather than a secondary consequence of an underlying lung pathology such as pulmonary fibrosis.

3)The pie charts depict subcluster EC population proportions in AO, CA and PA. Proportions are shown as medians and interquartile range. Especially in PA cells, these are large interquartile ranges. Can the authors comment on this and what statistics were used for theseanalyses?

Immuno 30.2% (16.45% - 43.95%)
Stress Response 28.3% (9.75% - 46.85%)
Vascular tone 42.1% (21.9% - 62.3%)

Similarly, for Figure 7A, these are again high interquartile ranges. What statistics were used for these analyses? Can the authors present the individual percentages per patient for a better sense of the heterogeneity? While human tissue is a precious resource and this is a large n for a study such as this, a comment on the heterogeneity would be helpful, particularly in the context of applying different treatment strategies to different subtypes of PH based on these subsets which appear to be heterogenous across different patients.

Immuno 55.4% (31.9% - 78.9%)
Stress Response 6.58% (4.97% - 8.19%)
Vascular tone 39.7% (12.1% - 67.3%)

We thank the reviewer for this constructive criticism and have replaced all Piecharts with Boxplots that show median and IQR ranges with the sample specific cell % values

overlayed. In addition, we expanded the Figure legend description explaining the statistical approach.

Statistical analysis was performed on a per-sample basis. Percentages of cells assigned to each endothelial archetype were computed, and the median and interquartile range (IQR) were calculated across samples for each compartment (AO/CA/PA), diagnosis (Donor/PAH/PHPF), or cell cycle status (S/G2M/G1). This per-sample approach ensures equal weighting of biological replicates and prevents disproportionate influence of samples with large cell numbers, with wider IQRs reflecting true inter-sample biological heterogeneity rather than technical pooling effects.

4) In Figure 3 (right), could more detail and clarity be provided to the UMAP on the right? The terminology "nodes (crossings), leaves (endpoints) and the calculated pseudotime (right)" is unclear and it is difficult to appreciate the numbers/colors of the circled nodes on the plot.

PCA was computed on scaled gene expression data and batch-corrected using Harmony with sample identity as a covariate, followed by UMAP dimensionality reduction. Monocle3 was used to learn a principal graph capturing the global structure of the PAEC population. A root node was manually specified at the bottom-left region of the UMAP based on marker expression and spatial positioning. Pseudotime was calculated as the geodesic distance along the inferred graph from this root, with increasing values indicating progression along the proposed trajectory.

Based on this reviewer's concern, we have expanded the figure legend and added better explanation of the methodology involved in trajectory analysis.

Minor comments:

1) In lines 63-67, the authors state "Despite the accumulating knowledge on transcriptional and functional changes of the endothelium in diseases involving the pulmonary vasculature 23,28–32 65, and growing interest in endothelial heterogeneity, PAECs have not been examined at high resolution, particularly in clinically distinct subtypes such as PAH and PH associated with pulmonary fibrosis (PH-PF)." I would consider the additions of citations of the following manuscripts of single cell RNA sequencing and ensure the prior statement remains correct.

We thank the reviewer for bringing these important publications to our attention and have added these citations now to the introduction of our revised manuscript. We carefully took the findings of these publications into account and can confidently say, that the statements of our manuscript still remain correct.

2) Line 88-89 The cell markers and cell lineage description should align in this sentence for simplicity and understanding of the reader i.e. PTPRC, ACTA2, EPCAM1, PDGRA and

LYVE1 (Supplemental Table 1, 2 – not finding any of the supplemental tables) for immune, smooth muscle, epithelial, fibroblast, and lymphatic cells respectively.

We thank this reviewer for this suggestion and adapted the gene order in the suggested fashion. We apologize for the availability issues of Supplemental tables. This was remedied with the support of figshare and the links to Supplemental Tables are functional now. For convenience, please find the figshare link below and an additional zenodo link.

https://figshare.com/s/b987505589a97c95d8cd

https://zenodo.org/records/17793613?preview=1&token=eyJhbGciOiJIUzUxMiJ9.eyJpZCI6IjQ0ZTAzYzMwLTgxMTMtNDY4Yy04N2VlLTQ3OGYzNzYxNjBiNCIsImRhdGEiOnt9LCJyYW5kb20iOiIzNjRmOGZlNzc0OTA5ZWRjMWQxZWM3MjJkODkxMDAxMyJ9.1fzDbTzLioegl7_No8XXtGgn1qJWwEOCzytSSdtIsSLhsifneBNmDATNnDRHgOjwHsoJNBdMT-qfbnw6R4G0BQ

3) In Supplemental Figure 1C, how do you account for the ACTA2 expression in the endothelial population? Does this signify endothelial to mesenchymal transition or an expected expression of ACTA2.

Thank you for bringing this to our attention. We have, adjusted the colour palettes and scale range to better depict that ACTA2 expression is well within expected limits. In the UMAPs below one can appreciate the difference in ACTA2 expression between SMC and other cell types, including EC.

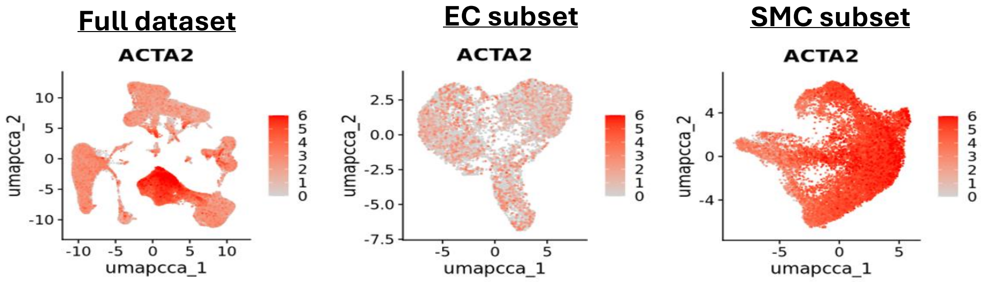

(A) UMAP of the full scRNA-seq dataset showing ACTA2 expression levels.

(B) UMAP of the endothelial cell (EC) subset showing ACTA2 expression.

(C) UMAP of the smooth muscle cell (SMC) subset showing ACTA2 expression.

In addition, we have conducted analysis of endothelial to mesenchymal transition, but could not identify any significant increase of EndoMT in any of the subpopulations or disease states. This new data is included in the revised manuscript (Supplemental Figure 13).

4) a. While it is stated in the cited paper Crnkovic S., Valzano F. et.al. 2022, could the authors confirm in the text that small and medium pulmonary arteries were those that were <5mm?

We have improved the manuscript to explicitly state vessel sizes and added an additional citation depicting the exact vessel sizes and their procurement method for interested parties[9].

b. For the Supplemental Figure 4 and line 138 how were pulmonary veins collected/processed? I could not find this methodology in the cited text.

While we specifically isolated PAs for scRNA we did not isolate veins for scRNAseq processing. In the images of fixed paraffin embedded tissue we showed the efficacy of our chosen markers in different vascular beds, including veins. We apologize for any unclarity in our manuscript.

c. How were precision-cut lung slices carried out? If the same methodology from this citation, please indicate this.

We have now adapted the manuscript to describe PCLS procurement and treatment. PCLS slides were procured by filling fresh lung pieces with 2.5% low melting agarose. A puncher was used to acquire 8 mm cylinders which were then cut using a VF210 (precisonary instruments), into 300 Subsequently PCLS were fixed in 4% buffered formaldehyde. The PCLs pieces underwent antigen retrieval via FLASH[10] and were then utilized for staining purposes similar to standard tissue slides. PCLS were additional cleared with a Benzyl benzoate/benzyl alcohol (BABB) solution and mounted on a glass slide with imaging spacers, preventing distortion and squeezing of tissue. This description has been added to the material and methods section of the revised manuscript.

d. Can the authors add information about how the pseudotime analysis was carried out in their methodology? See major comments.

Based on the reviewer's concern we have added a more in-depth description of pseudo time in the revised manuscript text and respective figure legend.

Additional Minor points:

a. In line 127, citation 25,34 not superscript.

b. In Figure 7E, red stain does not have a label.

c. In Figure 8A-C (right), what is the unit for the y axis?

d. In Supplemental Figure 2, it would be helpful to increase the height of the image in order to better see the genes on the Y axis.

e. Supplemental Figure 7A "stress response modulting"

f. Line 279-281, include reference to the Figure that demonstrates this point for clarity

We greatly appreciate the detailed evaluation of our manuscript and have adjusted all of the above-mentioned points within our revised manuscript. However, we were unable to stretch the graphs of Supplemental Figure 2 (revised Supplemental Figure 3) to accommodate up to 301 genes. Therefore, we kept the original heatmaps to show the results of our filtering strategy but also added an additional gene annotated heatmap of the top 50 genes per cluster. A full list of the involved genes can be accessed in Supplemental Tables 3,5 and 7.

Reviewer3:

One major comment is the small number of patient samples. At least n=5 patients each disease (i.e. 5 PAH and 5 PF-PH) should be included for the study to enhance reproducibility.

We thank the reviewer for raising this important point. We fully agree that increasing patient numbers would strengthen reproducibility. However, the availability of well-characterized human pulmonary arterial tissue from PAH and PF-PH patients is limited. Despite the limited number of patient samples, we observed highly consistent and homogeneous transcriptional patterns across the dataset, with the same endothelial subpopulations and marker profiles reproducibly detected across individuals. Importantly, key findings were further validated at the protein level by immunofluorescence stainings performed on independent lung tissue samples, providing multi-level confirmation of our main conclusions.

Other minor comments include 1) some references need to be updated, e.g. ref26, all references should be rechecked. 2) Fig 5C should use text instead of color to label Donor and PH samples for better clarity.

We are grateful for the careful consideration of our manuscript. We adjusted both points according to the reviewer's suggestion.

1. Adams, T. S. *et al.* Single-cell RNA-seq reveals ectopic and aberrant lung-resident cell populations in idiopathic pulmonary fibrosis. *Sci Adv* **6**, eaba1983 (2020).

2. Denzer, L., Muranyi, W., Schroten, H. & Schwerk, C. The role of PLVAP in endothelial cells. *Cell Tissue Res* **392**, 393–412 (2023).

3. Ochoa, C. D., Wu, S. & Stevens, T. New developments in lung endothelial heterogeneity: Von Willebrand factor, P-selectin, and the Weibel-Palade body. *Semin Thromb Hemost* **36**, 301–308 (2010).

4. Maeda, S. *et al.* Analysis of Intrapulmonary Vessels and Epithelial-Endothelial Interactions in the Human Developing Lung. *Lab Invest* **82**, 293–301 (2002).

5. Slenders, L. *et al.* Endothelial-to-mesenchymal transition gene signature derived from single-cell transcriptomics of human atherosclerotic tissue associates with stable plaque histological characteristics. *Vascul Pharmacol* **159**, 107498 (2025).

6. Piera-Velazquez, S. & Jimenez, S. A. Endothelial to Mesenchymal Transition: Role in Physiology and in the Pathogenesis of Human Diseases. *Physiol Rev* **99**, 1281–1324 (2019).

7. Tombor, L. S. *et al.* Single cell sequencing reveals endothelial plasticity with transient mesenchymal activation after myocardial infarction. *Nat Commun* **12**, 681 (2021).

8. Zhang, Q. *et al.* Endothelial Heterogeneity in the Response to Autophagy Drives Small Vessel Muscularization in Pulmonary Hypertension. *Circulation* **150**, 466–487 (2024).

9. Marsh, L. M. *et al.* The inflammatory cell landscape in the lungs of patients with idiopathic pulmonary arterial hypertension. *European Respiratory Journal* **51**, (2018).

10. Messal, H. A. *et al.* Antigen retrieval and clearing for whole-organ immunofluorescence by FLASH. *Nat Protoc* **16**, 239–262 (2021).

Reviewer1:

Major Comments:

1.) A non-negligible fraction of PAECs in the scRNA-seq dataset may derive from vasa vasorum residing within the walls of 2–5 mm pulmonary arteries. Although briefly mentioned in the discussion section, this possibility should be emphasized in the results, particularly since vasa vasorum ECs express both VWF and ACKR1 (Burns et al., Microvascular Research 2023; Thiriot et al., BMC Biology 2017), the latter being a marker of the immunomodulatory EC cluster identified by the authors. Additional images of isolated PAs stained for the study markers and VWF, as already performed, would help confirm or exclude a contribution from vasa vasorum ECs.

We thank the reviewer for highlighting the potential contribution of vasa vasorum endothelial cells to the PAEC population in our scRNA-seq dataset. As noted in the discussion, we agree that endothelial cells derived from the vasa vasorum located within the walls of large pulmonary arteries may contribute to the isolated PAEC population

To further address this point, we have included additional low-magnification images of stained pulmonary arteries (Additional Fig. 1A) and stitched views of entire vessels (Additional Fig. 1B), to visualize the spatial distribution of endothelial marker expression within the vessel wall. These images allow visualization of both the luminal endothelial layer and the surrounding vascular wall structures, including potential vasa vasorum. We added a description to the results and expanded the discussion accordingly with added citations.

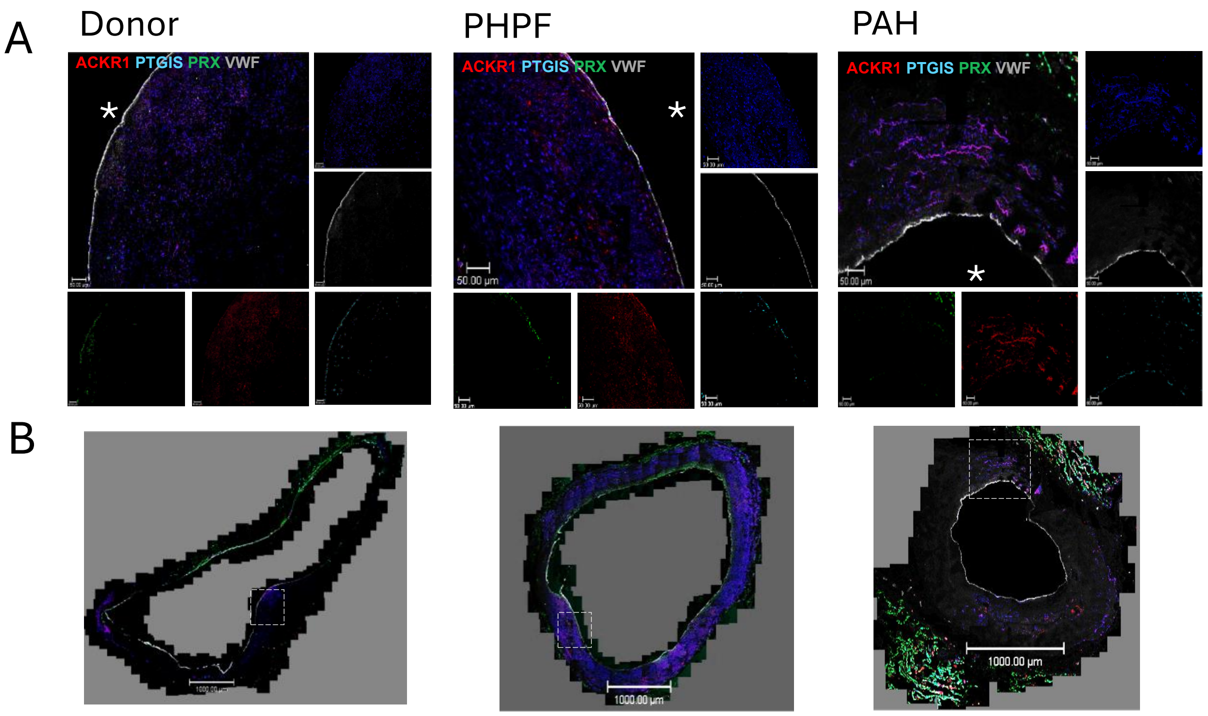

Additional Figure1: Immunofluorescent imaging of medium to small caliber arteries
(A) Representative images of the intimal endothelial layer from Donor, PAH, and PH-PF pulmonary arteries, shown together with the surrounding vessel wall to illustrate the spatial relationship between the luminal endothelium and underlying vascular structures.

(B) Stitched low-magnification images of entire pulmonary artery segments from Donor, PAH, and PH-PF samples, providing an overview of endothelial marker distribution across the full vessel circumference and wall architecture.

2.) The authors appropriately applied CCA and Harmony for the batch correction during integration and for clustering. Could batch effects also be addressed for the differential gene expression analyses? CCA and Harmony are typically applied to a subset of genes for sample integration, as shown here, but are not appropriate for differential expression tests.

We thank the reviewer for their comment regarding the handling of batch effects during differential gene expression (DEG) analysis.

In our study, batch correction using CCA and Harmony was performed during the dataset integration and clustering stages to enable identification of shared endothelial cell populations across samples. As noted by the reviewer, these approaches are generally applied to a subset of highly variable genes and are designed to align datasets in low dimensional space for cell identity inference rather than for statistical testing of gene expression differences[1,2].

Importantly, it is not generally recommended to perform differential expression testing on batch corrected expression matrices. Integration methods such as CCA, Harmony, or other latent space alignment approaches modify the gene expression structure to remove batch driven variation and therefore can distort the original gene level counts required for valid statistical inference. As a result, current best practices recommend performing differential expression testing on the original (non-integrated) expression data while accounting for potential batch effects within the statistical model when appropriate[3]

To address the reviewer's suggestion, we performed the differential expression analysis using the MAST framework while incorporating batch as a latent variable in the model (via the latent.vars parameter in Seurat's FindAllMarkers). The analysis was otherwise conducted using the same parameters and post processing filters used in the original manuscript:

- adjusted p-value < 0.05
- cluster expression percentage (pct.1) ≥ 0.2
- expression in other clusters (pct.2) ≤ 0.2
- avg_log2FC ≥ 0.5

The analysis yielded highly consistent results with the markers presented in the manuscript, with the same cluster specific gene expression patterns observed across the three endothelial artery subclusters (Additional Fig.2 A–C). Inclusion of batch as a latent variable in the MAST model resulted in minor differences in the composition of the DEG lists. Importantly, the core cluster defining markers and biological pathways remained unchanged across analyses, indicating that batch effects were not driving the reported endothelial subpopulation signatures.

Thus, accounting for batch as a latent variable did not materially change the biological conclusions or marker gene sets reported in the study.

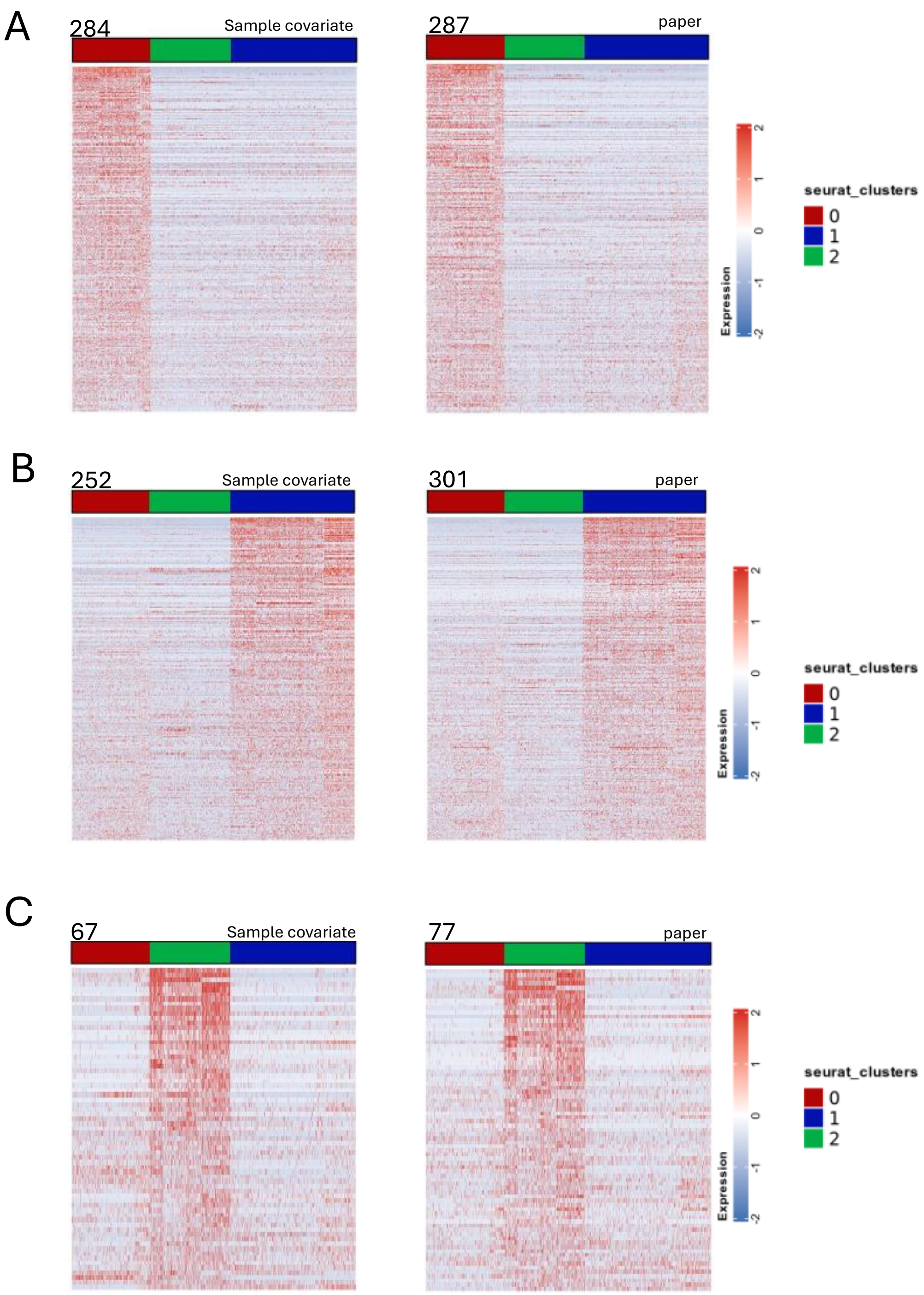

Additional Figure 2: Differential expression test comparison

(A) Heatmaps comparing cluster 0 (immunomodulatory PAECs) were DEG is performed with added sample covariate calculation (left) and as presented in the paper (right)

(B) Heatmaps comparing cluster 1 (vascular tone modulatory PAECs) were DEG is performed with added sample covariate calculation (left) and as presented in the paper (right)

(C) Heatmaps comparing cluster 2 (vascular plasticity modulatory PAECs) were DEG is performed with added sample covariate calculation (left) and as presented in the paper (right)

Minor Comments:

1).In the updated Fig. 7B–C, PAH and PH-PF samples lacking "vascular plasticity modulatory" ECs (value = 0) should be included in the analysis.

We thank the reviewer for this suggestion.

We have updated the analysis and revised Figures 7B–C and Supplemental Table 19 to include samples with zero counts for this endothelial subpopulation when calculating the percentage of cells per sample.

2.) How was arteriole identity confirmed for vessels shown in the new Figures 2D, 7D, and 7E? The PH vessels in Fig. 7 do not display clear remodeling features.

We thank the reviewer for raising this point regarding arteriole identification and the apparent degree of remodelling in the vessels shown in Figures 2D, 7D, and 7E.

Arteriole identity was determined using anatomical location, vessel morphology, and tissue autofluorescence patterns. Specifically, pulmonary arterioles were identified based on their proximity to bronchi/bronchioles[4] and characteristic vessel wall structure[5], which are well established criteria in lung histology for distinguishing pulmonary arteries from veins. In the pulmonary circulation, arteries typically run in parallel with the bronchial tree and are therefore readily identifiable based on their anatomical relationship to bronchi or bronchioles in tissue sections.

In addition, intrinsic tissue autofluorescence of elastin within the internal elastic lamina and surrounding connective tissue provided a consistent landmark that helped delineate arterial structures in the multiplex immunofluorescence images. Elastin rich vascular structures in the lung are well known to exhibit strong autofluorescence[6], which can be useful for identifying arterial walls but can also complicate fluorescence signal separation during multiplex imaging.

For the representative images shown in Figures 2D, 7D, and 7E, we selected regions in which signal compensation for the antibody channels was optimal, enabling clearer visualization of the endothelial markers (ACKR1, PTGIS, and PRX). In some areas of the pulmonary hypertension (PH) tissues, particularly those adjacent to bronchi or with increased extracellular matrix deposition, strong autofluorescence from elastin and surrounding structures facilitated identification of arterial vessels but complicated fluorescence compensation, reducing the ability to accurately visualize antibody derived signals. Therefore, while other regions displayed more pronounced vascular remodeling, they exhibited elevated autofluorescence that interfered with reliable signal separation in the multiplex imaging workflow.

To clarify this point, we have included additional uncompensated images (Additional Fig. 3A) demonstrating how autofluorescence and anatomical context were used to identify pulmonary arterioles in the tissue sections. Representative vessels with more extensive remodeling were also observed in the PH samples (examples shown in Additional Fig. 3B–3C), although these regions were not used in the primary figures due to the aforementioned imaging limitations.

Taken together, arteriole identity in our study was determined based on histological location relative to bronchi, vessel morphology, and autofluorescence defined elastic structures, and not solely on the presence of remodeling features. The vessels shown in Figures 2D, 7D, and 7E are therefore representative pulmonary arterioles even though they do not display the most severe remodeling observed in the PH tissues.

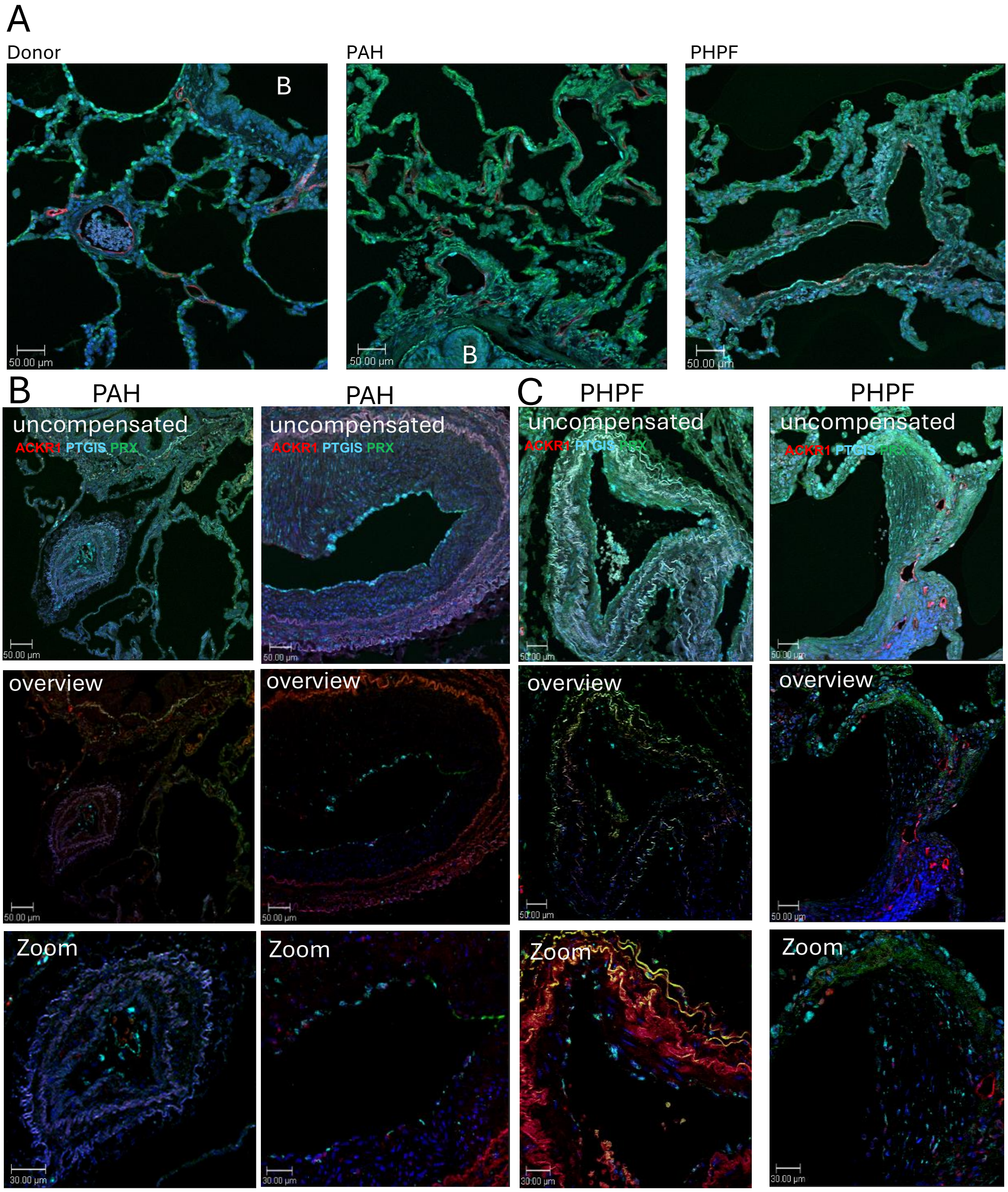

Additional Figure 3: Uncompensated vessel stainings

(A) Images corresponding to those shown in the manuscript displayed without fluorescence compensation to visualize surrounding tissue architecture and intrinsic autofluorescence used to identify arterial structures (ACKR1, red; PTGIS, blue; PRX, green; nuclei, DAPI).

(B,C) Representative pulmonary arterial vessels from PAH tissue (B) and PH-PF tissue (C) demonstrating vascular remodeling. In these regions, strong intrinsic autofluorescence from elastin-rich vascular structures aids vessel identification but interferes with fluorescence compensation, limiting clean separation of antibody-derived signals

3.) Intimal hyperplasia is most prominent in small-diameter arterioles in PH. Given the larger PA diameter analyzed here (2-5mm diameter), this may explain the absence of apoptosis-resistant, hyperproliferative, or EndoMT signatures in the PAEC scRNA-seq data. That point could be further discussed in the discussion section of the manuscript.

We thank the reviewer for this suggestion. We have expanded the discussion in accordance with the reviewer's suggestion to clarify the vessel size analysed and its implications for the observed endothelial signatures. In addition, because the samples were derived from end stage human disease, it is likely that major remodeling events had already occurred. We therefore clarify that the endothelial states captured in our scRNA seq dataset likely reflect stabilized pathological cell states rather than actively proliferating or transitioning endothelial populations.

1. Stuart, T. *et al.* Comprehensive integration of single-cell data. *Cell* **177**, 1888-1902.e21 (2019).

2. Korsunsky, I. *et al.* Fast, sensitive, and accurate integration of single cell data with Harmony. *Nat Methods* **16**, 1289–1296 (2019).

3. Luecken, M. D. & Theis, F. J. Current best practices in single-cell RNA-seq analysis: a tutorial. *Mol Syst Biol* **15**, MSB188746 (2019).

4. Hsia, C. C. W., Hyde, D. M., Ochs, M. & Weibel, E. R. An Official Research Policy Statement of the American Thoracic Society/European Respiratory Society: Standards for Quantitative Assessment of Lung Structure. *Am J Respir Crit Care Med* **181**, 394–418 (2010).

5. Townsley, M. I. Structure and composition of pulmonary arteries, capillaries and veins. *Compr Physiol* **2**, 675–709 (2012).

6. Croce, A. C. & Bottiroli, G. Autofluorescence Spectroscopy and Imaging: A Tool for Biomedical Research and Diagnosis. *Eur J Histochem* **58**, 2461 (2014).
